# Facile Synthesis of NH-Free 5-(Hetero)Aryl-Pyrrole-2-Carboxylates by Catalytic C–H Borylation and Suzuki Coupling

**DOI:** 10.3390/molecules25092106

**Published:** 2020-04-30

**Authors:** Saba Kanwal, Noor-ul- Ann, Saman Fatima, Abdul-Hamid Emwas, Meshari Alazmi, Xin Gao, Maha Ibrar, Rahman Shah Zaib Saleem, Ghayoor Abbas Chotana

**Affiliations:** 1 Department of Chemistry and Chemical Engineering, Syed Babar Ali School of Science & Engineering (SBASSE), Lahore University of Management Sciences (LUMS), Lahore 54792, Pakistan; kanwalsa91@gmail.com (S.K.); noorulannjml01@gmail.com (N.-u.-A.); saman_fatima@live.com (S.F.); mahaibrar@gmail.com (M.I.); rahman.saleem@lums.edu.pk (R.S.Z.S.); 2 Core Labs, King Abdullah University of Science and Technology (KAUST), Thuwal 23955-6900, Saudi Arabia; abdelhamid.emwas@kaust.edu.sa; 3 Computer, Electrical and Mathematical Sciences and Engineering (CEMSE) Division, Computational Bioscience Research Center (CBRC), King Abdullah University of Science and Technology (KAUST), Thuwal 23955-6900, Saudi Arabia; meshari.alazmi@kaust.edu.sa (M.A.); xin.gao@kaust.edu.sa (X.G.); 4 College of Computer Science and Engineering, University of Ha’il, P.O. Box 2440, Ha’il 81481, Saudi Arabia

**Keywords:** borylation, Suzuki coupling, NH-Free, 5-aryl pyrrole-2-carboxylates, iridium-catalyzed, heteroaryl substituted pyrroles, 2,3′-bipyrrole

## Abstract

A convenient two-step preparation of NH-free 5-aryl-pyrrole-2-carboxylates is described. The synthetic route consists of catalytic borylation of commercially available pyrrole-2-carboxylate ester followed by Suzuki coupling without going through pyrrole N–H protection and deprotection steps. The resulting 5-aryl substituted pyrrole-2-carboxylates were synthesized in good- to excellent yields. This synthetic route can tolerate a variety of functional groups including those with acidic protons on the aryl bromide coupling partner. This methodology is also applicable for cross-coupling with heteroaryl bromides to yield pyrrole-thiophene, pyrrole-pyridine, and 2,3’-bi-pyrrole based bi-heteroaryls.

## 1. Introduction

**Figure 1:**
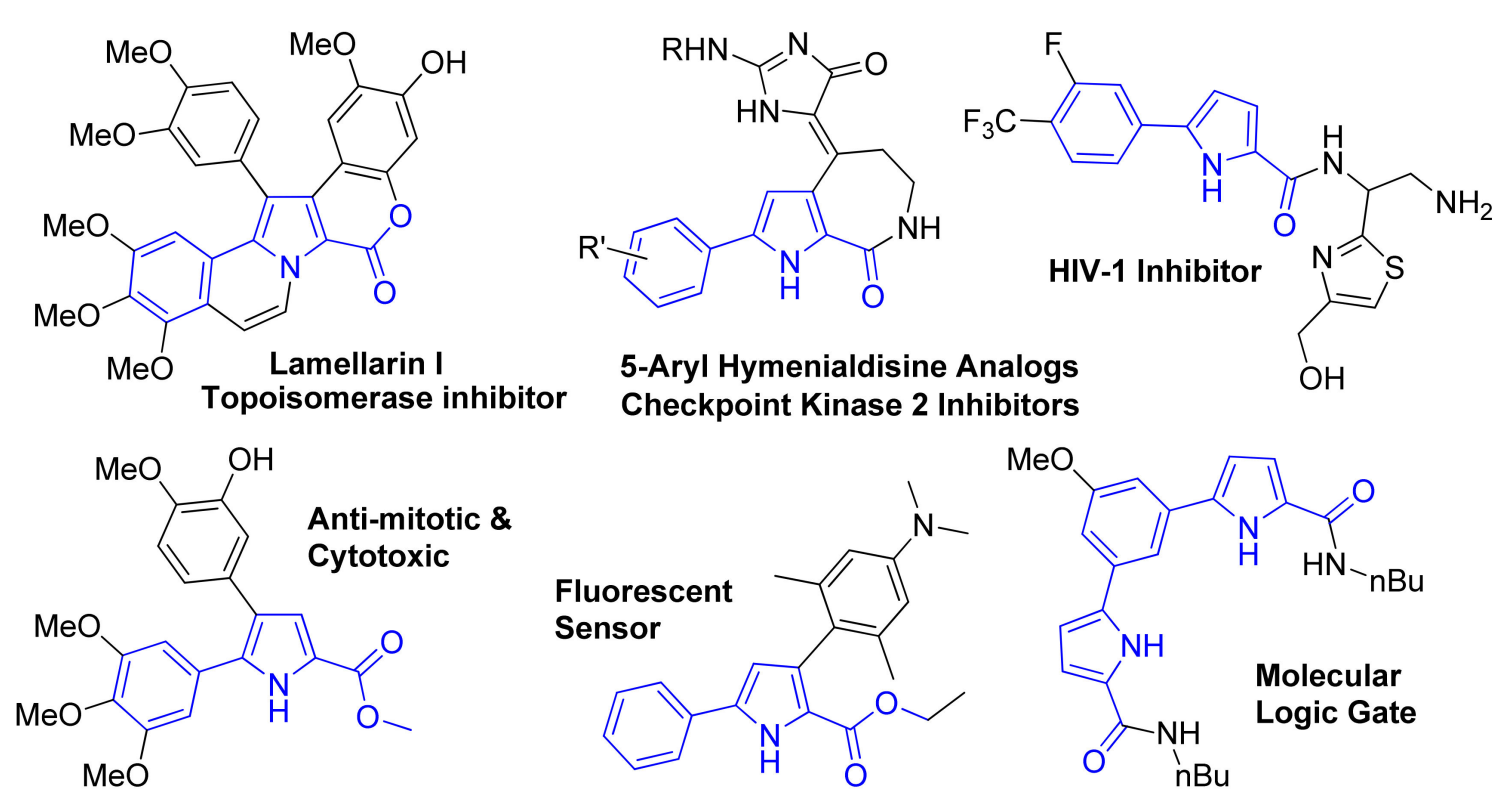
Selected examples of 5-arylpyrrole-2-carboxylate based natural products, biologically active compounds, and organic materials.

5-Aryl 1*H*-Pyrrole-2-carboxylate esters constitute an important class of pyrrole derivatives [1,2]. This structural motif is present in several natural products and their analogs such as Lamellarins [3,4,5,6,7] (topoisomerase I inhibitor, MDR reversal agent, and anti-HIV agent), and arylated hymenialdisine [8,9] (ChK2 inhibitor), as well as in several other biologically active compounds with anti-HIV [10,11,12,13,14], antibacterial [15], antimitotic [16], and cytotoxic [17] activities (Figure 1). 5-Aryl 1*H*-Pyrrole-2-carboxylate esters and their derivatives have also found applications, for example, as organic fluorescent materials [18,19], anion receptors/molecular logic gates [20], and as building blocks for metal organic frameworks [21] and helical asymmetric architectures [22,23]. As a consequence of their widespread applications, there has been burgeoning interest in developing new and efficient methodologies for quick access to this structural motif.

**Figure 2:**
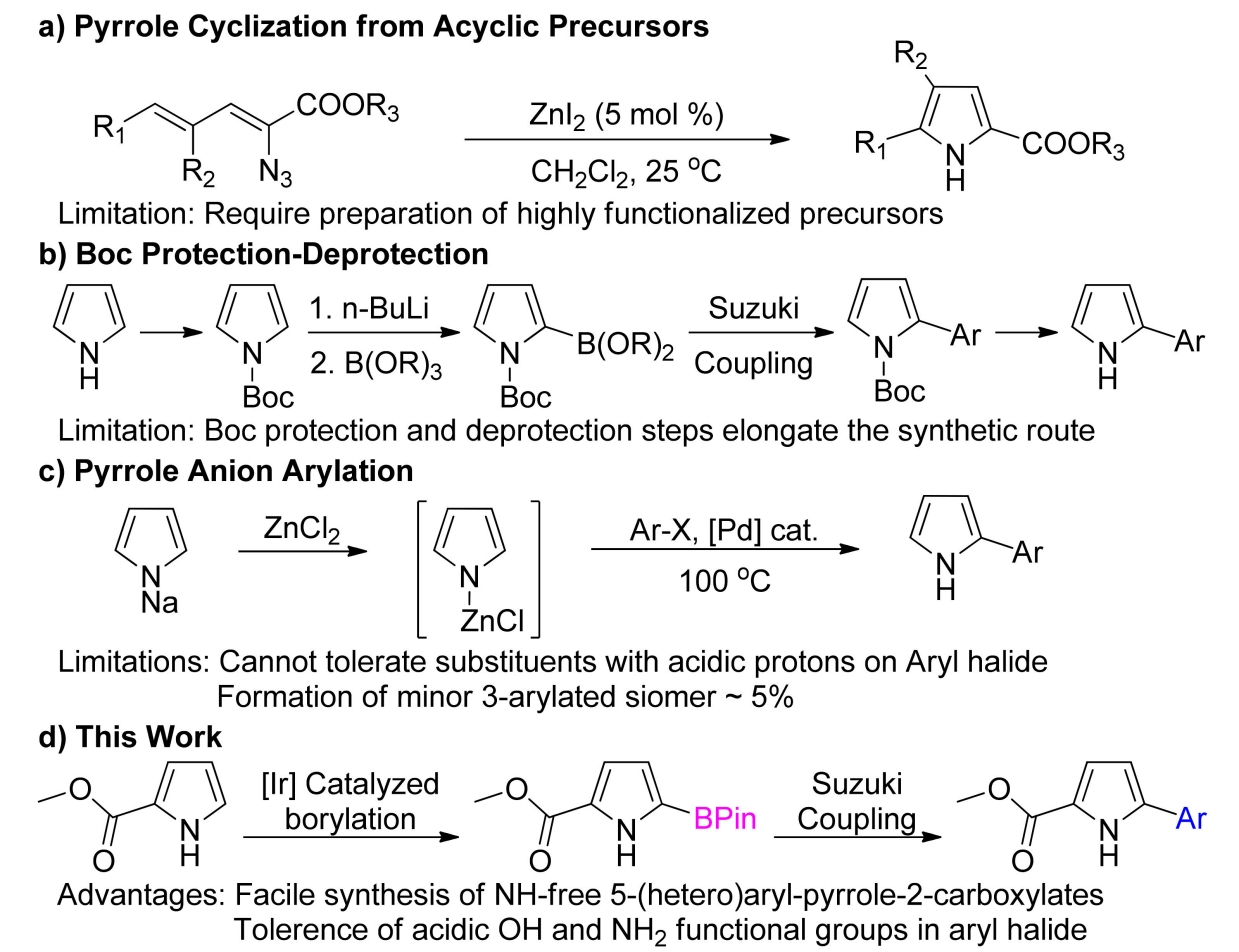
Various routes for the synthesis of aryl substituted pyrroles.

Traditional approaches to access 5-aryl pyrrole-2-carboxylates consist of long protracted routes involving construction of pyrrole ring from acyclic precursors [24,25,26]. During the last decade, several new methodologies have also been developed for the pyrrole cyclization reaction including multicomponent reactions [27], cycloadditions [28,29,30,31,32,33,34], Michael additions [35], isomerization [36], rearrangement [37], and photocatalysis [38,39]. A major disadvantage of these cyclization reactions is the preparation of highly functionalized precursors (Figure 2).

With the advent of transition metal-catalyzed reactions [40,41,42,43,44], derivatization of preformed pyrrole ring has grown as an alternate strategy for the synthesis of arylated pyrroles. However, preparation of pyrrole-based organometallic reagents employing halogen-metal exchange requires Boc-protection of the acidic N-H proton (Figure 2) [45]. To circumvent the preparation of organometallic reagents, direct arylation reactions have evolved. Unfortunately, direct arylation reactions are generally limited to N-protected pyrroles [46,47,48,49,50,51], and have been reported to be incompatible for the installation of highly electron-rich aryl groups [52]. Moreover, due to very harsh reaction conditions limiting the functional group tolerance, such arylations are rendered incapable of preparing heteroaryl substituted pyrroles. Hence, there is a need to develop new short synthetic routes devoid of these limitations which can also facilitate access to unconventional scaffolds in search of novel medicinally active compounds and organic materials.

Transition metal-catalyzed Suzuki coupling reactions require much milder conditions as compared to direct arylation reactions thereby allowing a broad functional group compatibility. Pyrrole 2-carboxylate esters, which are readily commercially available, can potentially be an excellent starting point for the preparation of 5-arylpyrrole-2-carboxylates by electrophilic halogenation and subsequent Suzuki coupling. However, halogenation of pyrrole 2-carboxylate esters yields a 1:1 mixture of 4- and 5-functionalized pyrroles whose separation is cumbersome [53,54]. Isomerically pure 5-halo substituted pyrrole-2-carboxylate require tedious preparation and are generally synthesized in N-protected form [55,56]. Preparation of the corresponding 5-boronic ester derivative also requires N-protection [57] or blockage of the 3- and 4-positions [21,22,58]. This N-protection/deprotection and blocking elongates the synthetic route and also reduces atom economy. Development of a Suzuki coupling route for the synthesis of 5-arylpyrrole-2-carboxylates that obviates the protection-deprotection and blocking steps is highly desirable.

The groups of Smith-Maleczka and Hartwig-Miyaura have reported an iridium-catalyzed borylation reaction which can directly functionalize aromatic C−H bond to a boronic ester group [59,60,61,62]. This methodology has also been successfully utilized to prepare heteroarylboronic esters of pyrroles [63,64,65], indoles [66,67,68,69], thiophenes [70], pyridines [71,72,73], and other heteroaromatics [74]. This reaction can tolerate pyrrole N-H functional group and hence does not need N-protection for the synthesis of pyrroleboronic esters. N-H free pyrroles are easily borylated on the 2-position while N-protection can be used to direct borylation at the 3-position [75,76]. N-Boc protected 3-borylated pyrroles have been employed in Suzuki coupling to access 3-arylpyrroles [77]. On the other side, N−H unprotected pyrroleboronic esters have been much less utilized for Suzuki coupling [78]. Our group has been interested in exploring catalytic C–H borylation reactions for organic synthesis [72,79,80,81,82,83,84,85,86]. A recent report about failure of installation of highly electron-rich aromatic substituent on pyrrole by direct arylation [52] prompted us to investigate Suzuki coupling route for this purpose. Herein, we describe the application of iridium-catalyzed borylation−Suzuki coupling route for a concise two-step synthesis of 5-aryl pyrrole-2-carboxylates.

## 2. Results and Discussion

**Scheme 1:**
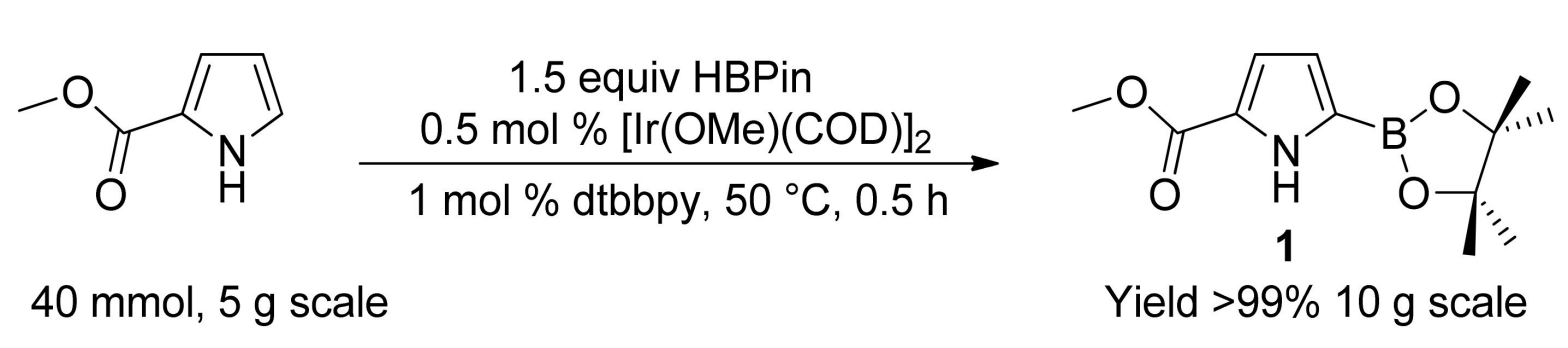
Iridium-catalyzed borylation of methyl 1-*H* pyrrole 2-carboxylate.

Methyl-1*H*-pyrrole-2-carboxylate was subjected to iridium-catalyzed borylation, by using a slightly modified literature protocol [87], to prepare methyl 5-(4,4,5,5-tetramethyl-1,3,2-dioxaborolan-2-yl)-1*H*-pyrrole-2-carboxylate (Scheme 1). Pinacol borane (H−BPin) was preferred over bis(pinacolato)diboron (B_2_Pin_2_) as the borylating agent because of its ability to solubilize pyrrole substrate in the absence of any solvent. The borylation reaction was scaled up to 40 mmol scale and the borylated pyrrole was isolated on 10-gram scale with >99% yield.

**Scheme 2:**
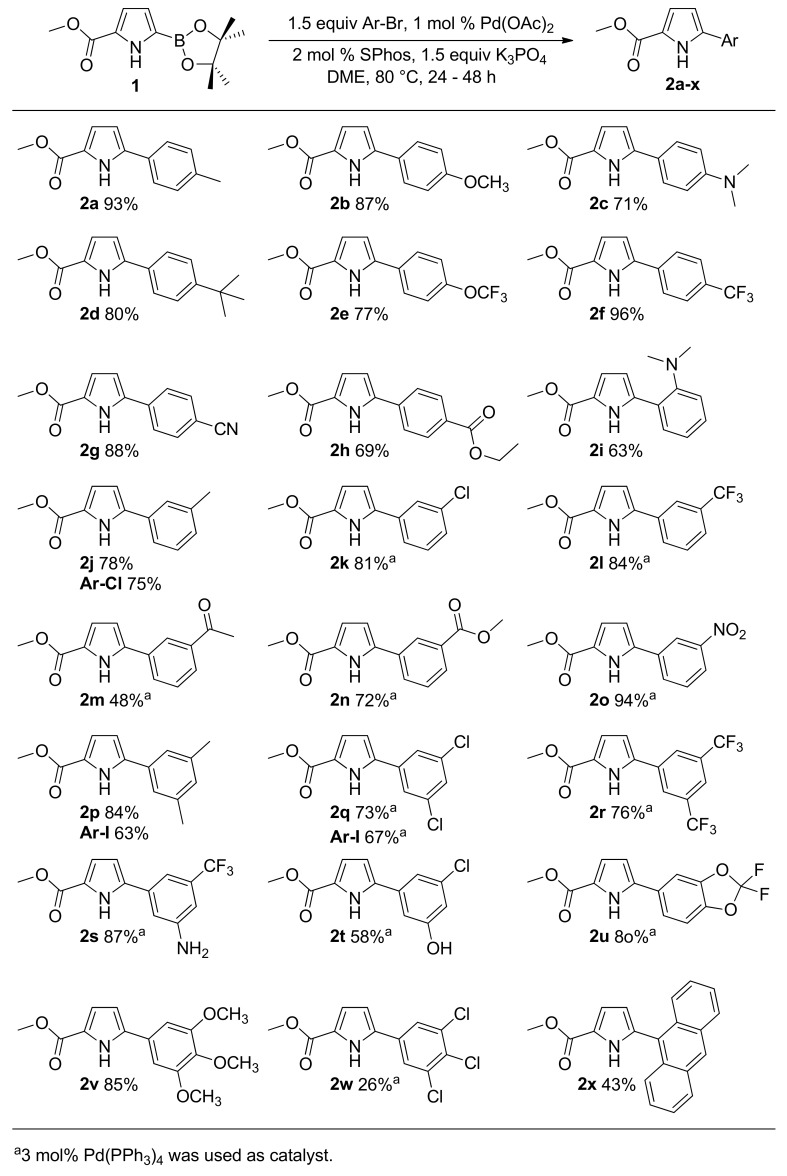
Suzuki coupling reactions of methyl 5-(4,4,5,5-tetramethyl-1,3,2-dioxaborolan-2-yl)-1*H*-pyrrole-2-carboxylate with various aryl bromides.

This N-H free borylated pyrrole has a long shelf life as no apparent decomposition was detected by GC-MS even after two years. The borylated pyrrole **1** was subsequently subjected to Suzuki coupling to synthesize 5-aryl substituted pyrrole-2-carboxylates. The pyrrole boronic ester easily underwent Suzuki-coupling with (hetero)aryl bromides using Buchwald’s Pd(OAc)_2_/SPhos catalyst system [88] as well as by the Pd(PPh_3_)_4_ catalyst (Scheme 2).

A variety of electron-rich and electron-deficient aryl bromides were utilized as coupling partners. Aryl bromides having *para* (entries **2a** – **2h**), *meta* (entries **2j** – **2o**), and *ortho* substituents (entries **2i** and **2x**) were successfully employed in Suzuki coupling and the corresponding 5-arylated pyrroles were isolated in good to excellent yields. Further, the reaction proceeded well with disubstituted (entries **2p** – **2u**), trisubstituted (entries **2v** and **2w**), and tetrasubstituted (entry **2x**) aryl bromides. Entry **2v** shows installation of highly electron-rich aromatic ring which was not possible via direct arylation [52]. Chloro-substituted aryl bromides (entries **2k**, **2q**, and **2w**) were selectively coupled at the C–Br bond. Aryl bromides with acidic protons (entries **2s** and **2t**) were also tolerated demonstrating the advantage of this route over pyrrole anion arylation protocol reported by Sadighi et al. [89]. Besides aryl bromides, aryl iodides (entries **2p** and **2q**) and aryl chlorides (**2j**) can also be utilized.

**Scheme 3:**
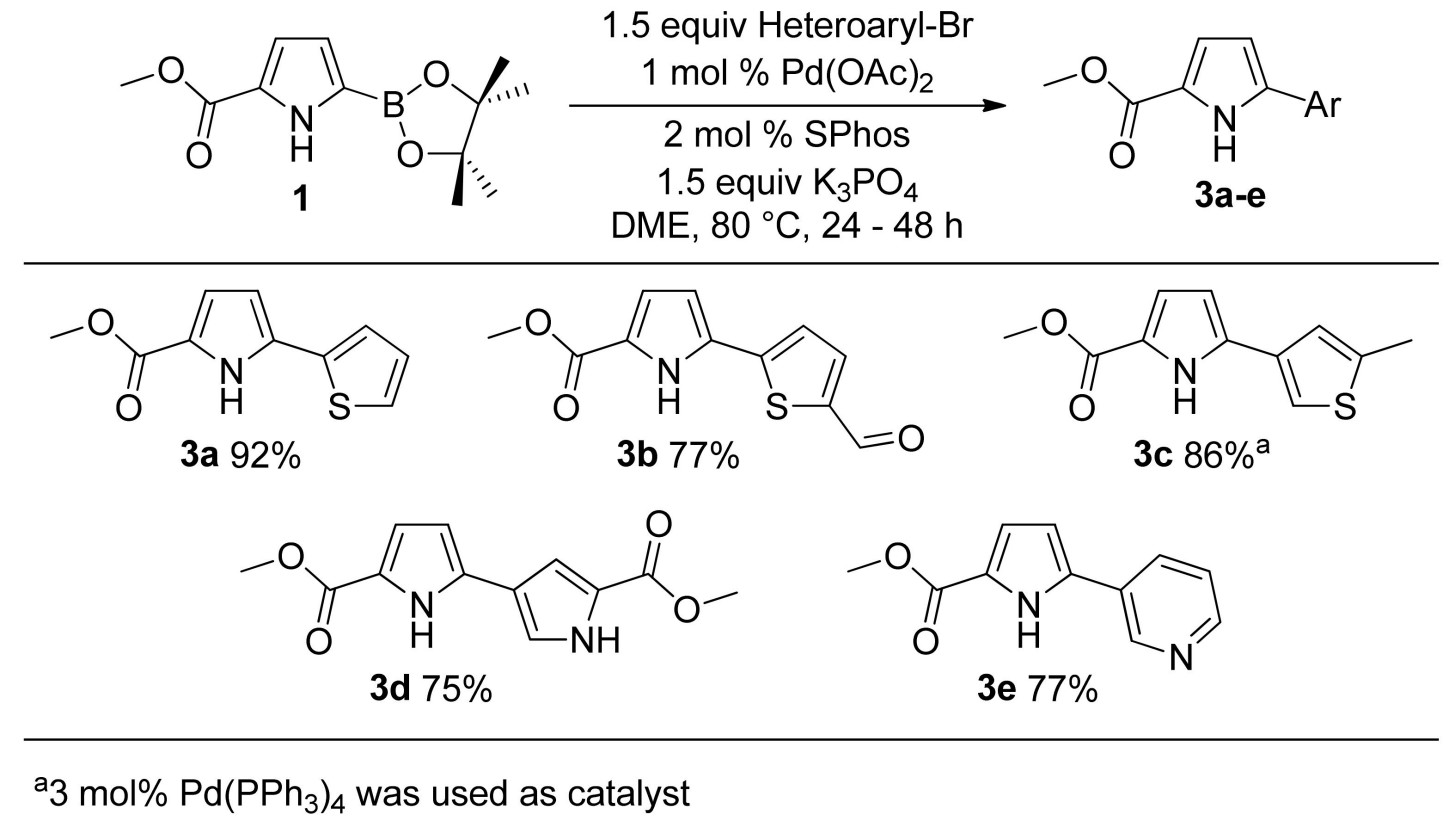
Suzuki couplings involving heteroaryl bromides.

Suzuki coupling with heteroaryl halides was also examined to synthesize 5-heteroaryl substituted pyrrole-2-carboxylates (Scheme 3). Heteroaryl bromides of thiophene (entries **3a**–**3c**) [34], pyrrole (**3d**) [90], and pyridine (**3e**) [38] all gave excellent isolated yields of corresponding bi-heteroaryl products. During the formation of **3d**, very small amounts (~5–7%) of two homocoupling products (originating by the homocouplings of boronic ester, and bromopyrrole, with themselves) were also observed by GC-MS. However, the cross-coupled product was formed as the major product and was isolated in 75% yield. This entry (**3d**) again signifies the advantage of the current route over direct pyrrole arylation protocols, which cannot be used to prepare such NH-free 2,3’-bi-pyrroles [91,92].

## 3. Materials and Methods

### 3.1. General Considerations and Starting Materials

All reactions were carried out under nitrogen atmosphere, without the use of glove box or Schlenk line. Chemicals and reagents were purchased from Sigma-Aldrich Corp^®^ (St. Louis, MO, USA), Combi-Blocks, Inc. (San Diego, CA, USA), and Strem Chemicals, Inc. (Newburyport, MA, USA), and were used without further purification unless otherwise noted. Ethyl acetate, n-hexane and dichloromethane were purchased from local suppliers and were distilled before use. Catalytic borylation and all the Suzuki cross-coupling reactions were carried out in inert atmosphere in 25 mL Schlenk flasks (0–4 mm Valve, 175 mm OAH) purchased from Chemglass Life Sciences. Analytical thin-layer chromatography (TLC) was carried out using 200 µm thick silica gel 60 matrix TLC Plates (Aluminum (Al) Silica, indicator F–254, EMD Millipore). Visualization was achieved under a UV lamp (254 nm and 365 nm). Column chromatography was carried out using SiliaFlash^®^ P60 (particle size: 40–63 µm, 230–400 mesh) purchased from SiliCycle Inc. All reported yields are for isolated materials. Reaction times and yields are not optimized. HBPin = pinacolborane; dtbbpy = 4,4′-di-tert-butyl-2,2′-bipyridyl; SPhos = 2-dicyclohexylphosphino-2′,6′-dimethoxybiphenyl.

Infrared spectra were recorded as neat using a Bruker Alpha-P IR instrument in the ATR geometry with a diamond ATR unit. Melting points were taken on Electrothermal IA9100 melting point apparatus and are uncorrected. Reactions were monitored by a GC–MS operating in EI mode. Column type: TR-5MS, 5% phenyl polysilphenylene-siloxane, 30 m × 0.25 mm ID × 0.25 µm. GC–MS method: injector 250 °C, oven 50 °C (1 min), 50 to 250 °C (20 °C min^–1^), 250 °C (10 min); carrier gas: He (1.5 mL min^–1^). Accurate mass determinations (HRMS) were obtained using an Orbitrap mass spectrometer.

^1^H NMR spectra (see Supplementary Materials) were recorded at 700.130 MHz and ^13^C NMR spectra were recorded at 176.048 MHz at ambient temperatures. The chemical shifts in ^1^H NMR spectra are reported using TMS as internal standard and were referenced with the residual proton resonances of the corresponding deuterated solvent (CDCl_3_: 7.26 ppm). The chemical shifts in the ^13^C NMR spectra are reported relative to TMS (δ = 0) or the central peak of CDCl_3_ (δ = 77.23) for calibration. The abbreviations used for the chemical shifts are as; s (singlet), d (doublet), t (triplet), q (quartet), dd (doublet of doublet), tt (triplet of triplet), tq (triplet of quartet), ttd (triplet of triplet of doublet), m (unresolved multiplet), and br (broad). All coupling constants are apparent *J* values measured at the indicated field strengths. In ^13^C NMR spectra of arylboronic ester, the carbon atom attached to the boron atom of BPin group is typically not observed due to broadening from and coupling with boron.

*Methyl 5-(4,4,5,5-tetramethyl-1,3,2-dioxaborolan-2-yl)-1H-pyrrole-2-carboxylate (**1**)* In a fume hood, an oven dried Schlenk flask equipped with magnetic stirring bar was filled with nitrogen and evacuated (three cycles). Under nitrogen atmosphere [Ir(OMe)(COD)]_2_ (133 mg, 0.2 mmol, 0.5 mol% Ir), 4,4′-di-*tert*-butyl-2,2′-bipyridine (107 mg, 0.40 mmol, 1 mol%), and pinacolborane (HBPin) (8.706 mL, 7.679 g, 60 mmol, 1.5 equiv) were added. Methyl-1*H*-pyrrole-2-carboxylate (5.0 g, 40 mmol, 1 equiv) was added under nitrogen atmosphere. The Schlenk flask was closed and the reaction mixture was heated at 50 °C in an oil bath for 0.5 h. The progress of reaction was monitored by GC-MS and TLC. Upon completion of reaction, the Schlenk flask was cooled to room temperature and exposed to air. The reaction mixture was taken out by dissolving in dichloromethane and the volatiles were removed under reduced pressure using rotary evaporator. The crude product was purified by column chromatography. Colorless solid; yield: 10.02 g (99.9%); mp 121–123 °C; *R_f_* = 0.45 (hexanes–CH_2_Cl_2_ 1:1). FT-IR (ATR): 3321, 2994, 2956, 1688, 1556, 1438, 1379, 1303, 1214, 1197, 1138, 1000, 852, 775, 693, 616, 528 cm^−1^. ^1^H NMR (700 MHz, CDCl_3_): δ = 9.48 (br s, 1 H), 6.91 (apparent t, *J* = 2.8 Hz, 1 H), 6.77 (apparent t, *J* = 2.8 Hz, 1 H), 3.86 (s, 3 H), 1.33 (s, 12 H, 4 CH_3_ of BPin). ^13^C NMR {^1^H} (176 MHz, CDCl_3_): δ = 161.2 (C=O), 126.5 (C), 120.6 (CH), 115.5 (CH), 84.2 (2 C), 51.6 (OCH_3_), 24.7 (4 CH_3_ of BPin). GC-MS (EI): *m*/z (%) = 251 (74) (M)^+^, 236 (21), 220 (13), 208 (86), 204 (23), 194 (38), 190 (12), 176 (100), 165 (21), 150 (42), 134 (18), 120 (23). HRMS (APCI-Orbitrap): *m*/*z* [M + H]^+^ calcd for C_12_H_19_BNO_4_: 252.14017; found: 252.13957.

### 3.2. Suzuki Coupling

#### 3.2.1. General Suzuki Procedure A Employing Pd(OAc)_2_ and 2-Dicyclohexylphosphino-2′,6′-dimethoxybiphenyl (SPhos)

In a fume hood, an oven dried Schlenk flask equipped with magnetic stirring bar was filled with nitrogen and evacuated (three cycles). Under nitrogen atmosphere palladium acetate Pd(OAc)_2_ (2.24 mg, 0.01 mmol, 1 mol%), 2-dicyclohexylphosphino-2′,6′-dimethoxybiphenyl (SPhos) (8.2 mg, 0.02 mmol, 2 mol%), aryl bromide (1.5 mmol, 1.5 equiv), methyl 5-(4,4,5,5-tetramethyl-1,3,2-dioxaborolan-2-yl)-1*H*-pyrrole-2-carboxylate (251 mg, 1 mmol, 1 equiv), potassium phosphate (K_3_PO_4_) (318 mg, 1.5 mmol, 1.5 equiv), and dimethoxyethane (DME) (1.5 mL) were added. Liquid substrates were added via micropipette under nitrogen atmosphere. The Schlenk flask was closed and the reaction mixture was heated at 60–80 °C in an oil bath. The progress of reaction was monitored by GC-MS and TLC. Upon completion of reaction, the Schlenk flask was cooled to room temperature and exposed to air. The reaction mixture was taken out by dissolving in dichloromethane and the volatiles were removed under reduced pressure using a rotary evaporator. The crude product was purified by column chromatography (silica gel; hexanes–CH_2_Cl_2_).

#### 3.2.2. General Suzuki Procedure B Employing Palladium Tetrakistriphenylphosphine Pd(PPh_3_)_4_

The general Suzuki Procedure A was employed using palladium tetrakistriphenylphosphine Pd(PPh_3_)_4_ (34.7 mg, 0.03 mmol, 3 mol%) as catalyst instead of Pd(OAc)_2_/SPhos.

Synthesis of 5-Aryl 1*H*-Pyrrole-2-Carboxylate Esters.

*Methyl 5-(p-tolyl)-1H-pyrrole-2-carboxylate (**2a**)* The general Suzuki procedure A was applied to methyl 5-(4,4,5,5-tetramethyl-1,3,2-dioxaborolan-2-yl)-1*H*-pyrrole-2-carboxylate (251 mg, 1 mmol, 1 equiv) and 4-bromotoluene (185 µL, 257 mg, 1.5 mmol, 1.5 equiv) for 48 h. Colorless solid; yield: 200 mg (93%); mp 168–170 °C; *R_f_* = 0.4 (hexanes–CH_2_Cl_2_ 1:3). FT-IR (ATR): 3315, 2944, 2852, 1677, 1470, 1437,1336, 1264, 1243, 1003, 786, 658 cm^−1^. ^1^H NMR (700 MHz, CDCl_3_): δ = 9.59 (br s, 1 H), 7.49 (d, *J* = 7.8 Hz, 2 H), 7.20 (d, *J* = 7.8 Hz, 2 H), 6.95 (apparent t, *J* = 2.8 Hz, 1 H), 6.50 (apparent t, *J* = 3.0 Hz, 1 H), 3.87 (s, 3 H), 2.37 (s, 3 H). ^13^C NMR {^1^H} (176 MHz, CDCl_3_): δ = 161.8 (C=O), 137.7 (C), 137.2 (C), 129.6 (2 CH), 128.5 (C), 124.7 (2 CH), 122.6 (C), 116.9 (CH), 107.6 (CH), 51.6 (OCH_3_), 21.2 (CH_3_). GC-MS (EI): *m*/z (%) = 215 (100) (M)^+^, 183 (95), 155 (43), 140 (11), 128 (13), 115 (9). HRMS (ESI-Orbitrap): *m*/*z* [M + H]^+^ calcd for C_13_H_14_NO_2_: 216.10191; found: 216.10195.

*Methyl 5-(4-methoxyphenyl)-1H-pyrrole-2-carboxylate (**2b**)* The general Suzuki procedure A was applied to methyl 5-(4,4,5,5-tetramethyl-1,3,2-dioxaborolan-2-yl)-1*H*-pyrrole-2-carboxylate (251 mg, 1 mmol, 1 equiv) and 4-bromoanisole (188 µL, 281 mg, 1.5 mmol, 1.5 equiv) for 36 h. Colorless solid; yield: 202 mg (87%); mp 151–152 °C, lit[39] 144–146 °C; *R_f_* = 0.45 (hexanes–CH_2_Cl_2_ 1:3). FT-IR (ATR): 3320, 3116, 3003, 2913, 2835, 1683, 1611, 1563, 1474, 1436, 1269, 1243, 1188, 1121, 1046, 1025, 938, 919, 874, 830, 792, 759, 659, 610 cm^−1^. ^1^H NMR (700 MHz, CDCl_3_): δ = 9.40 (br s, 1 H), 7.51 (d, *J* = 8.7 Hz, 2 H), 6.94 (m, 3 H), 6.44 (apparent t, *J* = 3.0, 1 H), 3.87 (s, 3 H), 3.84 (s, 3 H). ^13^C NMR {^1^H} (176 MHz, CDCl_3_): δ = 161.7 (C=O), 159.3 (C), 137.0 (C), 126.2 (2 CH), 124.2 (C), 122.4 (C), 117.0 (CH), 114.4 (2 CH), 107.1 (CH), 55.4 (OCH_3_), 51.5 (OCH_3_). GC-MS (EI): *m*/z (%) = 231 (73) (M)^+^, 199 (100), 184 (7), 171 (45), 156 (21), 145 (9), 141 (3), 128 (21). HRMS (ESI-Orbitrap): *m*/*z* [M + H]^+^ calcd for C_13_H_14_NO_3_: 232.09682; found: 232.09864.

*Methyl 5-(4-(dimethylamino)phenyl)-1H-pyrrole-2-carboxylate (**2c**)* The general Suzuki procedure A was applied to methyl 5-(4,4,5,5-tetramethyl-1,3,2-dioxaborolan-2-yl)-1*H*-pyrrole-2-carboxylate (251 mg, 1 mmol, 1 equiv) and 4-bromo-*N,N*-dimethylaniline (299 mg, 1.5 mmol, 1.5 equiv) for 48 h. Colorless solid; yield: 170 mg (71%); mp 175–176 °C; *R_f_* = 0.40 (hexanes–CH_2_Cl_2_ 1:3). FT-IR (ATR): 3327, 3269, 2945, 2926, 1675, 1612, 1557, 1474, 1421, 1147, 1067, 1041, 104, 813, 787 cm^−1^. ^1^H NMR (700 MHz, CDCl_3_): δ = 9.24 (br s, 1 H), 7.45 (d, *J* = 8.3 Hz, 2 H), 6.94 (s, 1 H), 6.74 (d, *J* = 8.3 Hz, 2 H), 6.39 (s, 1 H), 3.86 (s, 3 H), 2.99 (s, 6 H). ^13^C NMR {^1^H} (176 MHz, CDCl_3_): δ = 161.7 (C=O), 150.1 (C), 137.9 (C), 125.8 (2 CH), 121.6 (C), 119.5 (C), 117.1 (CH), 112.5 (2 CH), 106.2 (CH), 51.4 (OCH_3_), 40.4 (2 CH_3_). GC-MS (EI): *m*/z (%) = 244 (56) (M)^+^, 212 (100), 184 (45), 169 (15), 158 (7), 140 (7), 115 (3), 106 (3). HRMS (ESI-Orbitrap): *m*/*z* [M + H]^+^ calcd for C_14_H_17_N_2_O_2_: 245.12845; found: 245.12843.

*Methyl 5-(4-(tert-butyl)phenyl)-1H-pyrrole-2-carboxylate (**2d**)* The general Suzuki procedure A was applied to methyl 5-(4,4,5,5-tetramethyl-1,3,2-dioxaborolan-2-yl)-1*H*-pyrrole-2-carboxylate (251 mg, 1 mmol, 1 equiv) and 1-bromo-4-*tert*-butylbenzene (260 µL, 320 mg, 1.5 mmol, 1.5 equiv) for 36 h. Colorless solid; yield: 205 mg (80%); mp 152–153 °C, lit[39] 149–150 °C; *R_f_* = 0.40 (hexanes–CH_2_Cl_2_ 1:3). FT-IR (ATR): 3293, 3259, 2953, 2863, 1681, 1573, 1287, 1195, 1004, 825, 669 cm^−1^. ^1^H NMR (700 MHz, CDCl_3_): δ = 9.50 (br s, 1 H), 7.52 (d, *J* = 8.1 Hz, 2 H), 7.42 (d, *J* = 8.1 Hz, 2 H), 6.95 (s, 1 H), 6.51 (d, *J* = 2.6 Hz, 1 H), 3.87 (s, 3 H), 1.33 (s, 9 H). ^13^C NMR {^1^H} (176 MHz, CDCl_3_): δ = 161.8 (C=O), 150.9 (C), 137.1 (C), 128.5 (C), 125.9 (2 CH), 124.6 (2 CH), 122.6 (C), 116.9 (CH), 107.7 (CH), 51.6 (OCH_3_), 34.6 (C), 31.2 (3 CH_3_). GC-MS (EI): *m*/z (%) = 257 (32) (M)^+^, 242 (40), 225 (10), 210 (100), 182 (5), 170 (2), 167 (2), 155 (12), 141 (2), 127 (2), 115 (2). HRMS (ESI-Orbitrap): *m*/*z* [M + H]^+^ calcd for C_16_H_20_NO_2_: 258.14886; found: 258.14870.

*Methyl 5-(4-(trifluoromethoxy)phenyl)-1H-pyrrole-2-carboxylate (**2e**)* The general Suzuki procedure A was applied to methyl 5-(4,4,5,5-tetramethyl-1,3,2-dioxaborolan-2-yl)-1*H*-pyrrole-2-carboxylate (251 mg, 1 mmol, 1 equiv) and 1-bromo-4-(trifluoromethoxy) benzene (223 µL, 362 mg, 1.5 mmol, 1.5 equiv) for 48 h. Colorless solid; yield: 220 mg (77%); mp 165–166 °C; *R_f_* = 0.40 (hexanes–CH_2_Cl_2_ 1:3). FT-IR (ATR): 3314, 3030, 2957, 1687, 1562, 1473, 1439, 1208, 1190, 1149, 1050, 967, 850, 755, 732, 657 cm^−1^. ^1^H NMR (700 MHz, CDCl_3_): δ = 9.78 (br s, 1 H), 7.63 (d, *J* = 8.7 Hz, 2 H), 7.25 (d, *J* = 8.7 Hz, 2 H), 6.96 (apparent t, *J* = 3.3 Hz, 1 H), 6.53 (apparent t, *J* = 3.3 Hz, 1 H), 3.87 (s, 3 H). ^13^C NMR {^1^H} (176 MHz, CDCl_3_): δ = 161.8 (C=O), 148.6 (C), 135.7 (C), 130.2 (C), 126.3 (2 CH), 123.5 (C), 121.5 (2 CH), 120.4 (q, ^1^*J*_C-F_ = 258 Hz, OCF_3_), 117.0 (CH), 108.5 (CH), 51.7 (OCH_3_). GC-MS (EI): *m*/z (%) = 285 (43) (M)^+^, 253 (100), 225 (86), 199 (40), 184 (5), 156 (38), 139 (23), 133 (7), 128 (27), 101 (5). HRMS (ESI-Orbitrap): *m*/*z* [M + H]^+^ calcd for C_13_H_11_F_3_NO_3_: 286.06855; found: 286.06873.

*Methyl 5-(4-(trifluoromethyl)phenyl)-1H-pyrrole-2-carboxylate (**2f**)* The general Suzuki procedure A was applied to methyl 5-(4,4,5,5-tetramethyl-1,3,2-dioxaborolan-2-yl)-1*H*-pyrrole-2-carboxylate (251 mg, 1 mmol, 1 equiv) and 4-bromobenzotrifluoride (210 µL, 338 mg, 1.5 mmol, 1.5 equiv) for 48 h. Colorless solid; yield: 257 mg (96%); mp 198–199 °C, lit[39] 196–197 °C; *R_f_* = 0.20 (hexanes–CH_2_Cl_2_ 1:3). FT-IR (ATR): 3312, 1686, 1617, 1586, 1523, 1475, 1329, 1250, 1194, 1109, 1048, 1007, 801, 760, 692 cm^−1^. ^1^H NMR (700 MHz, CDCl_3_): δ = 9.70 (br s, 1 H), 7.70 (d, *J* = 8.1 Hz, 2 H), 7.65 (d, *J* = 8.1 Hz, 2 H), 6.98 (apparent t, *J* = 3.1 Hz, 1 H), 6.63 (apparent t, *J* = 3.1 Hz, 1 H), 3.89 (s, 3 H). ^13^C NMR {^1^H} (176 MHz, CDCl_3_): δ = 161.7 (C=O), 135.2 (C), 134.6 (C), 129.4 (q, ^2^*J*_C-F_ = 32.7 Hz, C), 126.0 (q, ^3^*J*_C-F_ = 3.1 Hz, 2 CH), 124.8 (2 CH), 124.1 (C), 124.0 (q, ^1^*J*_C-F_ = 271.6 Hz, CF_3_), 117.0 (CH), 109.3 (CH), 51.8 (OCH_3_). GC-MS (EI): *m*/z (%) = 269 (56) (M)^+^, 237 (100), 218 (6), 209 (40), 189 (10), 183 (26), 163 (2), 158 (2), 140 (47), 133 (6). HRMS (ESI-Orbitrap): *m*/*z* [M + H]^+^ calcd for C_13_H_11_F_3_NO_2_: 270.07364; found: 270.07360.

*Methyl 5-(4-cyanophenyl)-1H-pyrrole-2-carboxylate (**2g**)* The general Suzuki procedure A was applied to methyl 5-(4,4,5,5-tetramethyl-1,3,2-dioxaborolan-2-yl)-1*H*-pyrrole-2-carboxylate (251 mg, 1 mmol, 1 equiv) and 4-bromobenzonitrile (273 mg, 1.5 mmol, 1.5 equiv) for 48 h. Colorless solid; yield: 198 mg (88%); mp 256–257 °C; *R_f_* = 0.20 (hexanes–CH_2_Cl_2_ 1:3). FT-IR (ATR): 3306, 2955, 2219, 1688, 1606, 1573, 1437, 1337, 1319, 1283, 1188, 1069, 1007, 939, 806, 726 cm^−1^. ^1^H NMR (700 MHz, CDCl_3_): δ = 9.39 (br s, 1 H), 7.70 (d, *J* = 8.1 Hz, 2 H), 7.65 (d, *J* = 8.1 Hz, 2 H), 6.98 (s, 1 H), 6.67 (apparent t, *J* = 2.8 Hz, 1 H), 3.90 (s, 3 H). ^13^C NMR {^1^H} (176 MHz, CDCl_3_): δ = 161.4 (C=O), 135.3 (C), 134.2 (C), 132.9 (2 CH), 124.8 (2 CH), 124.7 (C), 118.7 (C), 116.9 (CH), 110.8 (C), 110.1 (CH), 51.9 (OCH_3_). GC-MS (EI): *m*/z (%) = 226 (85) (M)^+^, 194 (100), 166 (46), 139 (25), 113 (7), 88 (2). HRMS (ESI-Orbitrap): *m*/*z* [M + H]^+^ calcd for C_13_H_11_N_2_O_2_: 227.08150; found: 227.08171.

*Methyl 5-(4-(ethoxycarbonyl)phenyl)-1H-pyrrole-2-carboxylate (**2h**)* The general Suzuki procedure A was applied to methyl 5-(4,4,5,5-tetramethyl-1,3,2-dioxaborolan-2-yl)-1*H*-pyrrole-2-carboxylate (251 mg, 1 mmol, 1 equiv) and ethyl 4-bromobenzoate (245 µL, 344 mg, 1.5 mmol, 1.5 equiv) for 48 h. Colorless solid; yield: 188 mg (69%); mp 168–169 °C; *R_f_* = 0.1 (hexanes–CH_2_Cl_2_ 1:3). FT-IR (ATR): 3317, 2983, 2966, 1703, 1683, 1607, 1474, 1436, 1367, 1260, 1187, 1067, 939, 863, 770, 758, 656 cm^−1^. ^1^H NMR (700 MHz, CDCl_3_): δ = 9.77 (br s, 1 H), 8.07 (d, *J* = 8.2 Hz, 2 H), 7.66 (dd, *J* = 8.2, 1.9 Hz, 2 H), 6.98 (dd, *J* = 3.6, 2.5 Hz, 1 H), 6.65 (apparent t, *J* = 3.5 Hz, 1 H), 4.39 (q, *J* = 7.1 Hz, 2 H), 3.89 (s, 3 H), 1.41 (t, *J* = 7.1 Hz, 3 H). ^13^C NMR {^1^H} (176 MHz, CDCl_3_): δ = 166.2 (C=O), 161.7 (C=O), 135.7 (C), 135.3 (C), 130.3 (2 CH), 129.3 (C), 124.4 (2 CH), 124.0 (C), 117.0 (CH), 109.4 (CH), 61.1 (CH_2_), 51.8 (OCH_3_), 14.3 (CH_3_). GC-MS (EI): *m*/z (%) = 273 (66) (M)^+^, 241 (100), 228 (10), 213 (67), 196 (68), 185 (8), 168 (15), 158 (6), 140 (18), 114 (5), 113 (6). HRMS (ESI-Orbitrap): *m*/*z* [M + H]^+^ calcd for C_15_H_16_NO_4_: 274.10738; found: 274.10780.

*Methyl 5-(2-(dimethylamino)phenyl)-1H-pyrrole-2-carboxylate (**2i**)* The general Suzuki procedure A was applied to methyl 5-(4,4,5,5-tetramethyl-1,3,2-dioxaborolan-2-yl)-1*H*-pyrrole-2-carboxylate (251 mg, 1 mmol, 1 equiv) and 2-bromo-*N,N*-dimethylaniline (215 µL, 300.1 mg, 1.5 mmol, 1.5 equiv) for 48 h. Light yellow liquid; yield: 154 mg (63%); *R_f_* = 0.30 (hexanes–CH_2_Cl_2_ 1:3). FT-IR (ATR): 3301, 3117, 2984, 2947, 2832, 2790, 1694, 1499, 1384, 1328, 1284, 1183, 1105, 995, 796, 749, 657 cm^−1^. ^1^H NMR (700 MHz, CDCl_3_): δ = 11.75 (br s, 1 H), 7.62 (dd, *J* = 8.1, 0.8 Hz, 1 H), 7.24 (m, 2 H), 7.12 (m, 1 H), 6.93 (dd, *J* = 3.8, 2.7 Hz, 1 H), 6.57 (dd, *J* = 3.8, 2.5 Hz, 1 H), 3.87 (s, 3 H), 2.71 (s, 6 H). ^13^C NMR {^1^H} (176 MHz, CDCl_3_): δ = 161.6 (C=O), 150.4 (C), 135.9 (C), 128.1 (CH), 128.0 (CH), 125.1 (C), 124.4 (CH), 121.8 (C), 120.4 (CH), 115.7 (CH), 108.0 (CH), 51.4 (OCH_3_), 44.7 (2 CH_3_). GC-MS (EI): *m*/z (%) = 244 (86) (M)^+^, 212 (95), 184 (100), 168 (26), 144 (17), 131 (13), 115 (13). HRMS (ESI-Orbitrap): *m*/*z* [M + H]^+^ calcd for C_14_H_17_N_2_O_2_: 245.12845; found: 245.12799.

*Methyl 5-(m-tolyl)-1H-pyrrole-2-carboxylate (**2j**)* The general Suzuki procedure A was applied to methyl 5-(4,4,5,5-tetramethyl-1,3,2-dioxaborolan-2-yl)-1*H*-pyrrole-2-carboxylate (251 mg, 1 mmol, 1 equiv) and 3-bromotoluene (182 µL, 257 mg, 1.5 mmol, 1.5 equiv) for 48 h. Light yellow solid; yield: 168 mg (78%); mp 119–121 °C, lit[39] 118–119 °C; *R_f_* = 0.20 (hexanes–CH_2_Cl_2_ 1:3). FT-IR (ATR): 3326, 3017, 2950, 2918, 2850, 1686, 1597, 1498, 1471, 1438, 1335, 1271, 1195, 1099, 1072, 1004, 955, 872, 794 cm^−1^. ^1^H NMR (700 MHz, CDCl_3_): δ = 9.57 (br s, 1 H), 7.39 (m, 2 H), 7.28 (t, *J* = 7.4, 1 H), 7.11 (d, *J* = 7.2 Hz, 1 H), 6.95 (s, 1 H), 6.52 (s, 1 H), 3.86 (s, 3 H), 2.39 (s, 3 H). ^13^C NMR {^1^H} (176 MHz, CDCl_3_): δ = 161.8 (C=O), 138.6 (C), 137.1 (C), 131.2 (C), 128.9 (CH), 128.6 (CH), 125.5 (CH), 122.8 (C), 121.9 (CH), 116.9 (CH), 107.9 (CH), 51.6 (OCH_3_), 21.5 (CH_3_). GC-MS (EI): *m*/z (%) = 215 (100) (M)^+^, 183 (95), 155 (34), 140 (9), 129 (12), 115 (12), 77 (2). HRMS (ESI-Orbitrap): *m*/*z* [M + H]^+^ calcd for C_13_H_14_NO_2_: 216.10191; found: 216.10165.

*Methyl 5-(3-chlorophenyl)-1H-pyrrole-2-carboxylate (**2k**)* The general Suzuki procedure A was applied to methyl 5-(4,4,5,5-tetramethyl-1,3,2-dioxaborolan-2-yl)-1*H*-pyrrole-2-carboxylate (251 mg, 1 mmol, 1 equiv) and 1-bromo-3-chlorobenzene (176 µL, 287 mg, 1.5 mmol, 1.5 equiv) for 48 h. Colorless solid; yield: 190 mg (81%); mp 133–134 °C; *R_f_* = 0.20 (hexanes–CH_2_Cl_2_ 1:1). FT-IR (ATR): 3317, 2953, 2926, 2851, 1736, 1689, 1599, 1460, 1435, 1332, 1309, 1271, 1149, 1101, 992, 924, 871, 777, 690, 626 cm^−1^. ^1^H NMR (700 MHz, CDCl_3_): δ = 9.72 (br s, 1 H), 7.58 (s, 1 H), 7.47 (d, *J* = 7.7 Hz, 1 H), 7.33 (t, *J* = 7.8 Hz, 1 H), 7.27 (d, *J* = 7.8 Hz, 1 H), 6.96 (apparent t, *J* = 3.1 Hz, 1 H), 6.55 (apparent t, *J* = 3.2 Hz, 1 H), 3.88 (s, 3 H). ^13^C NMR {^1^H} (176 MHz, CDCl_3_): δ = 161.7 (C=O), 135.4 (C), 134.9 (C), 133.1 (C), 130.2 (CH), 127.6 (CH), 124.9 (CH), 123.6 (C), 122.9 (CH), 116.9 (CH), 108.7 (CH), 51.8 (OCH_3_). GC-MS (EI): *m*/z (%) = 235 (66) (M)^+^, 237 (20) (M+2)^+^, 205 (31), 203 (100), 175 (26), 149 (12), 140 (43), 114 (6). HRMS (ESI-Orbitrap): *m*/*z* [M + H]^+^ calcd for C_12_H_11_ClNO_2_: 236.04728; found: 236.04736.

*Methyl 5-(3-(trifluoromethyl)phenyl)-1H-pyrrole-2-carboxylate (**2l**)* The general Suzuki procedure A was applied to methyl 5-(4,4,5,5-tetramethyl-1,3,2-dioxaborolan-2-yl)-1*H*-pyrrole-2-carboxylate (251 mg, 1 mmol, 1 equiv) and 3-bromobenzotrifluoride (210 µL, 338 mg, 1.5 mmol, 1.5 equiv) for 48 h. Light green solid; yield: 226 mg (84%); mp 152–154 °C; *R_f_* = 0.20 (hexanes–CH_2_Cl_2_ 1:3). FT-IR (ATR): 3331, 2952, 2923, 1683, 1445, 1321, 1283, 1194, 998, 896, 786, 757, 689, 616 cm^−1^. ^1^H NMR (700 MHz, CDCl_3_): δ = 9.86 (br s, 1 H), 7.82 (s, 1 H), 7.78 (d, *J* = 7.4 Hz, 1 H), 7.55–7.51 (m, 2 H), 6.98 (dd, *J* = 3.5, 2.5 Hz, 1 H), 6.61 (apparent t, *J* = 3.2 Hz, 1 H), 3.87 (s, 3 H). ^13^C NMR {^1^H} (176 MHz, CDCl_3_): δ = 161.8 (C=O), 135.4 (C), 132.2 (C), 131.5 (q, ^2^*J*_C-F_ = 32.0 Hz, C), 129.5 (CH), 127.9 (CH), 124.2 (q, ^3^*J*_C-F_ = 3.5 Hz, CH), 124.0 (q, ^1^*J*_C-F_ = 272 Hz, CF_3_), 123.8 (C), 121.6 (q, ^3^*J*_C-F_ = 3.6 Hz, CH), 117.0 (CH), 109.0 (CH), 51.8 (OCH_3_). GC-MS (EI): *m*/z (%) = 269 (56) (M)^+^, 237 (100), 218 (7), 209 (42), 189 (12), 183 (27), 163 (3), 158 (3), 140 (54), 133 (5). HRMS (ESI-Orbitrap): *m*/*z* [M + H]^+^ calcd for C_13_H_11_F_3_NO_2_: 270.07364; found: 270.07340.

*Methyl 5-(4-acetylphenyl)-1H-pyrrole-2-carboxylate (**2m**)* The general Suzuki procedure B was applied to methyl 5-(4,4,5,5-tetramethyl-1,3,2-dioxaborolan-2-yl)-1*H*-pyrrole-2-carboxylate (251 mg, 1 mmol, 1 equiv) and 3′-bromoacetophenone (198 µL, 299 mg, 1.5 mmol, 1.5 equiv) for 48 h. Colorless solid; yield: 117 mg (48%); mp 147–148 °C; *R_f_* = 0.20 (hexanes–CH_2_Cl_2_ 1:1). FT-IR (ATR): 3333, 2960, 1681, 1605, 1588, 1439, 1355, 1280, 1241, 1186, 1151, 1069, 955, 925, 798 cm^−1^. ^1^H NMR (700 MHz, CDCl_3_): δ = 9.68 (br s, 1 H), 8.17 (s, 1 H), 7.87 (d, *J* = 7.7 Hz, 1 H), 7.79 (d, *J* = 7.7 Hz, 1 H), 7.51 (t, *J* = 7.7 Hz, 1 H), 6.98 (s, 1 H), 6.62 (apparent t, *J* = 2.9 Hz, 1 H), 3.88 (s, 3 H), 2.65 (s, 3 H). ^13^C NMR {^1^H} (176 MHz, CDCl_3_): δ = 197.8 (C=O of ketone), 161.6 (C=O), 137.7 (C), 135.7 (C), 131.9 (C), 129.3 (CH), 129.2 (CH), 127.6 (CH), 124.1 (CH), 123.6 (C), 116.9 (CH), 108.7 (CH), 51.7 (OCH_3_), 26.8 (CH_3_). GC-MS (EI): *m*/z (%) = 243 (84) (M)^+^, 211 (82), 196 (100), 168 (16), 157 (4), 140 (17), 127 (2), 114 (7). HRMS (ESI-Orbitrap): *m*/*z* [M + H]^+^ calcd for C_14_H_14_NO_3_: 244.09682; found: 244.09700.

*Methyl 5-(3-(methoxycarbonyl)phenyl)-1H-pyrrole-2-carboxylate (**2n**)* The general Suzuki procedure B was applied to methyl 5-(4,4,5,5-tetramethyl-1,3,2-dioxaborolan-2-yl)-1*H*-pyrrole-2-carboxylate (251 mg, 1 mmol, 1 equiv) and methyl 3-bromobenzoate (323 mg, 1.5 mmol, 1.5 equiv) for 48 h. Colorless solid; yield: 187 mg (72%); mp 161–162 °C; *R_f_* = 0.20 (hexanes–CH_2_Cl_2_ 1:1). FT-IR (ATR): 3353, 3023, 2959, 2851, 1716, 1681, 1473, 1343, 1301, 1280, 1188, 1108, 1055, 1008, 977, 901, 781, 725, 644 cm^−1^. ^1^H NMR (700 MHz, CDCl_3_): δ = 9.43 (br s, 1 H), 8.23 (s, 1 H), 7.96 (d, *J* = 7.7 Hz, 1 H), 7.76 (d, *J* = 7.7 Hz, 1 H), 7.49 (t, *J* = 7.7 Hz, 1 H), 6.97 (apparent t, *J* = 2.8 Hz, 1 H), 6.62 (apparent t, *J* = 3.1Hz, 1 H), 3.96 (s, 3 H), 3.89 (s, 3 H). ^13^C NMR {^1^H} (176 MHz, CDCl_3_): δ = 166.7 (C=O), 161.5 (C=O), 135.5 (C), 131.6 (C), 130.9 (C), 129.2 (CH), 128.9 (CH), 128.6 (CH), 125.5 (CH), 123.5 (C), 116.8 (CH), 108.6 (CH), 52.4 (OCH_3_), 51.7 (OCH_3_). GC-MS (EI): *m*/z (%) = 259 (66) (M)^+^, 227 (100), 199 (8), 196 (30), 169 (35), 140 (18), 129 (2), 113 (5). HRMS (ESI-Orbitrap): *m*/*z* [M + H]^+^ calcd for C_14_H_14_NO_4_: 260.09173; found: 260.09149.

*Methyl 5-(3-nitrophenyl)-1H-pyrrole-2-carboxylate (**2o**)* The general Suzuki procedure B was applied to methyl 5-(4,4,5,5-tetramethyl-1,3,2-dioxaborolan-2-yl)-1*H*-pyrrole-2-carboxylate (251 mg, 1 mmol, 1 equiv) and 1-bromo-3-nitrobenzene (303 mg, 1.5 mmol, 1.5 equiv) for 48 h. Yellow solid; yield: 231 mg (94%); mp 201–203 °C; *R_f_* = 0.30 (hexanes–CH_2_Cl_2_ 1:3). FT-IR (ATR): 3324, 3302, 2955, 1675, 1566, 1496, 1463, 1340, 1310, 1265, 1193, 1150, 1105, 1005, 955, 899, 860, 703, 600, 579 cm^−1^. ^1^H NMR (700 MHz, CDCl_3_): δ = 9.57 (br s, 1 H), 8.42 (s, 1 H), 8.15 (dd, *J* = 8.1, 1.0 Hz, 1H), 7.89 (d, *J* = 7.7, 1 H), 7.60 (t, *J* = 8.0 Hz, 1 H), 6.99 (dd, *J* = 3.4, 2.5 Hz, 1 H), 6.68 (apparent t, *J* = 3.1 Hz, 1 H), 3.90 (s, 3 H). ^13^C NMR {^1^H} (176 MHz, CDCl_3_): δ = 161.4 (C=O), 148.8 (C), 133.9 (C), 132.9 (C), 130.2 (CH), 130.1 (CH), 124.4 (C), 122.1 (CH), 119.3 (CH), 116.9 (CH), 109.5 (CH), 51.6 (OCH_3_). GC-MS (EI): *m*/z (%) = 246 (64) (M)^+^, 214 (100), 200 (3), 186 (9), 168 (26), 156 (4), 140 (29), 128 (4), 113 (9). HRMS (ESI-Orbitrap): *m*/*z* [M + H]^+^ calcd for C_12_H_11_N_2_O_4_: 247.07133; found: 247.07103.

*Methyl 5-(3,5-dimethylphenyl)-1H-pyrrole-2-carboxylate (**2p**)* The general Suzuki procedure A was applied to methyl 5-(4,4,5,5-tetramethyl-1,3,2-dioxaborolan-2-yl)-1*H*-pyrrole-2-carboxylate (251 mg, 1 mmol, 1 equiv) and 1-bromo-3,5-dimethylbenzene (204 µL, 278 mg, 1.5 mmol, 1.5 equiv) for 48 h.

Colorless solid; yield: 192 mg (84%); mp 100–101 °C; *R_f_* = 0.50 (hexanes–CH_2_Cl_2_ 1:3). FT-IR (ATR): 3299, 3033, 2953, 2915, 2852, 1686, 1599, 1494, 1423, 1290, 1243, 1212, 1044, 1005, 862 cm^−1^. ^1^H NMR (700 MHz, CDCl_3_): δ = 9.31 (br s, 1 H), 7.18 (s, 2 H), 6.95–6.94 (m, 2 H), 6.51 (dd, *J* = 3.8, 2.7 Hz, 1 H), 3.87 (s, 3 H), 2.35 (s, 6 H). ^13^C NMR {^1^H} (176 MHz, CDCl_3_): δ = 161.6 (C=O), 138.6 (2 C), 137.1 (C), 131.1 (C), 129.5 (CH), 122.7 (C), 122.6 (2 CH), 116.8 (CH), 107.9 (CH), 51.5 (OCH_3_), 21.3 (2 CH_3_). GC-MS (EI): *m*/z (%) = 229 (71) (M)^+^, 197 (100), 169 (26), 154 (22), 143 (8), 129 (9), 115 (4). HRMS (ESI-Orbitrap): *m*/*z* [M + H]^+^ calcd for C_14_H_16_NO_2_: 230.11756; found: 230.11724.

*Methyl 5-(3,5-dichlorophenyl)-1H-pyrrole-2-carboxylate (**2q**)* The general Suzuki procedure B was applied to methyl 5-(4,4,5,5-tetramethyl-1,3,2-dioxaborolan-2-yl)-1*H*-pyrrole-2-carboxylate (251 mg, 1 mmol, 1 equiv) and 1-bromo-3,5-dichlorobenzene (339 mg, 1.5 mmol, 1.5 equiv) for 48 h. Colorless solid; yield: 197 mg (73%); mp 183–185 °C; *R_f_* = 0.50 (hexanes–CH_2_Cl_2_ 1:1). FT-IR (ATR): 3290, 3005, 2954, 1682, 1595, 1492, 1431, 1272, 1151, 1101, 992, 926, 848, 757, 703, 622 cm^−1^. ^1^H NMR (700 MHz, CDCl_3_): δ = 9.65 (br s, 1 H), 7.45 (s, 2 H), 7.28 (s, 1 H), 6.96 (s, 1 H), 6.56 (s, 1 H), 3.90 (s, 3 H). ^13^C NMR {^1^H} (176 MHz, CDCl_3_): δ = 161.6 (C=O), 135.6 (2 C), 134.1 (C), 133.9 (C), 127.4 (CH), 124.2 (C), 123.1 (2 CH), 116.9 (CH), 109.4 (CH), 51.9 (OCH_3_). GC-MS (EI): *m*/z (%) = 269 (37) (M)^+^, 271 (23) (M+2)^+^, 237 (100), 209 (25), 183 (14), 174 (46), 148 (6), 139 (4). HRMS (ESI-Orbitrap): *m*/*z* [M + H]^+^ calcd for C_12_H_10_Cl_2_NO_2_: 270.00831; found: 270.00846.

*Methyl 5-(3,5-bis(trifluoromethyl)phenyl)-1H-pyrrole-2-carboxylate (**2r**)* The general Suzuki procedure B was applied to methyl 5-(4,4,5,5-tetramethyl-1,3,2-dioxaborolan-2-yl)-1*H*-pyrrole-2-carboxylate (251 mg, 1 mmol, 1 equiv) and 1-bromo-3,5-bis(trifluoromethyl) benzene (259 µL, 440 mg, 1.5 mmol, 1.5 equiv) for 24 h. Colorless solid; yield: 256 mg (76%); mp 167–168 °C; *R_f_* = 0.60 (hexanes–CH_2_Cl_2_ 1:1). FT-IR (ATR): 3305, 3136, 2963, 1668, 1330, 1273, 1039, 1007, 680 cm^−1^. ^1^H NMR (700 MHz, CDCl_3_): δ = 10.19 (br s, 1 H), 8.00 (s, 2 H), 7.77 (s, 1 H), 7.00 (dd, *J* = 3.4, 2.5 Hz, 1 H), 6.69 (apparent t, *J* = 3.1 Hz, 1H), 3.86 (s, 3 H). ^13^C NMR {^1^H} (176 MHz, CDCl_3_): δ = 162.0 (C=O), 133.9 (C), 133.5 (C), 132.4 (q, ^2^*J*_C-F_ = 33.3 Hz, 2 C), 124.8 (C), 124.7 (d, ^3^*J*_C-F_ = 2.9 Hz, 2 CH), 123.2 (q, ^1^*J*_C-F_ = 272.6 Hz, 2 CF_3_), 120.8 (m, ^3^*J*_C-F_ = 3.6 Hz, CH), 117.2 (CH), 110.0 (CH), 51.9 (OCH_3_). GC-MS (EI): *m*/z (%) = 337 (32) (M)^+^, 305 (61), 286 (8), 277 (12), 257 (12), 251 (29), 238 (7), 231 (4), 207 (100), 182 (10), 158 (7). HRMS (ESI-Orbitrap): *m*/*z* [M + H]^+^ calcd for C_14_H_10_F_6_NO_2_: 338.06102; found: 338.06112.

*Methyl 5-(3-amino-5-(trifluoromethyl)phenyl)-1H-pyrrole-2-carboxylate (**2s**)* The general Suzuki procedure B was applied to methyl 5-(4,4,5,5-tetramethyl-1,3,2-dioxaborolan-2-yl)-1*H*-pyrrole-2-carboxylate (251 mg, 1 mmol, 1 equiv) and 3-bromo-5-(trifluoromethyl) aniline (211 µL, 360 mg, 1.5 mmol, 1.5 equiv) for 48 h. Colorless solid; yield: 248 mg (87%); mp 203–205 °C; *R_f_* = 0.10 (hexanes–CH_2_Cl_2_ 1:3). FT-IR (ATR): 3422, 3321, 3208, 3135, 3032, 2958, 1688, 1610, 1465, 1442, 1356, 1292, 1154, 1004, 882, 793, 751, 630 cm^−1^. ^1^H NMR (700 MHz, CDCl_3_): δ = 9.24 (br s, 1 H), 7.15 (s, 1 H), 6.98 (s, 1 H), 6.95 (apparent t, *J* = 3.1 Hz, 1 H), 6.83 (s, 1 H), 6.54 (apparent t, *J* = 3.1 Hz, 1 H), 3.96 (br s, 2 H), 3.89 (s, 3 H). ^13^C NMR {^1^H} (176 MHz, CDCl_3_): δ = 161.4 (C=O), 147.3 (C), 135.4 (C), 133.0 (C), 132.5 (q, ^2^*J*_C-F_ = 32 Hz, C), 123.9 (q, ^1^*J*_C-F_ = 272.2 Hz, CF_3_), 123.5 (C), 116.7 (CH), 113.6 (CH), 111.5 (d, *J* = 3.7, CH), 110.6 (d, *J* = 3.7, CH), 108.7 (CH), 51.7 (OCH_3_). GC-MS (EI): *m*/z (%) = 284 (42) (M)^+^, 252 (100), 233 (4), 224 (37), 205 (4), 198 (17), 176 (6), 155 (37), 151 (5), 128 (3), 126 (3). HRMS (ESI-Orbitrap): *m*/*z* [M + H]^+^ calcd for C_13_H_12_F_3_N_2_O_2_: 285.08454; found: 285.08465.

*Methyl 5-(3-chloro-5-hydroxyphenyl)-1H-pyrrole-2-carboxylate (**2t**)* The general Suzuki procedure B was applied to methyl 5-(4,4,5,5-tetramethyl-1,3,2-dioxaborolan-2-yl)-1*H*-pyrrole-2-carboxylate (251 mg, 1 mmol, 1 equiv) and 3-bromo-5-chlorophenol (311 mg, 1.5 mmol, 1.5 equiv) for 48 h. Colorless solid; yield: 145 mg (58%); mp 164 °C; *R_f_* = 0.1 (hexanes–CH_2_Cl_2_ 1:3). FT-IR (ATR): 3356, 3254, 3231, 2955, 1661, 1616, 1579, 1500, 1444, 1347, 1288, 1226, 1164, 1052, 1008, 991, 908, 878, 755, 696, 557 cm^−1^. ^1^H NMR (700 MHz, CDCl_3_): δ = 9.84 (br s, 1 H), 7.15 (s, 1 H), 7.07 (s, 1 H), 6.94 (s, 1H), 6.83 (s, 1 H), 6.60 (br s, 1 H), 6.53 (s, 1H), 3.91 (s, 3 H). ^13^C NMR {^1^H} (176 MHz, CDCl_3_): δ = 162.4 (C=O), 156.6 (C), 135.7 (C), 135.3 (C), 133.9 (C), 123.3 (C), 117.8 (CH), 117.3 (CH), 115.0 (CH), 109.8 (CH), 108.7 (CH), 51.9 (OCH_3_). GC-MS (EI): *m*/z (%) = 251 (42) (M)^+^, 253 (13) (M+2)^+^, 221 (31), 219 (100), 193 (8), 191 (25), 166 (3), 164 (9), 156 (28), 128 (6), 101 (4). HRMS (ESI-Orbitrap): *m*/*z* [M + H]^+^ calcd for C_12_H_11_ClNO_3_: 252.04220; found: 252.04268.

*Methyl 5-(2,2-difluorobenzo[d]*[1,3]*dioxol-5-yl)-1H-pyrrole-2-carboxylate (**2u**)* The general Suzuki procedure B was applied to methyl 5-(4,4,5,5-tetramethyl-1,3,2-dioxaborolan-2-yl)-1*H*-pyrrole-2-carboxylate (251 mg, 1 mmol, 1 equiv) and 5-bromo-2,2-difluorobenzo[d] [1,3]dioxole (204 µL, 356 mg, 1.5 mmol, 1.5 equiv) for 48 h. Colorless solid; yield: 224 mg (80%); mp 171–173 °C; *R_f_* = 0.30 (hexanes–CH_2_Cl_2_ 1:3). FT-IR (ATR): 3315, 3142, 2961, 1680, 1619, 1514, 1472, 1441, 1387, 1288, 1241, 1060, 1044, 1003, 964, 938, 826, 765, 704, 634 cm^−1^. ^1^H NMR (700 MHz, CDCl_3_): δ = 9.81 (br s, 1 H), 7.38 (s, 1 H), 7.32 (d, *J* = 8.3 Hz, 1 H), 7.09 (d, *J* = 8.3 Hz, 1 H), 6.96 (apparent t, *J* = 2.7 Hz, 1 H), 6.48 (apparent t, *J* = 2.8 Hz, 1 H), 3.89 (s, 3 H). ^13^C NMR {^1^H} (176 MHz, CDCl_3_): δ = 161.9 (C=O), 144.3 (C), 143.2 (C), 135.8 (C), 131.6 (t, ^1^*J*_C-F_ = 256 Hz, CF_2_), 128.0 (C), 123.3 (C), 120.4 (CH), 117.0 (CH), 109.9 (CH), 108.4 (CH), 106.5 (CH), 51.8 (OCH_3_). GC-MS (EI): *m*/z (%) = 281 (51) (M)^+^, 249 (100), 221 (77), 195 (21), 155 (24), 127 (43), 101 (5), 75 (3). HRMS (ESI-Orbitrap): *m*/*z* [M + H]^+^ calcd for C_13_H_10_F_2_NO_4_: 282.05724; found: 282.05724.

*Methyl 5-(3,4,5-trimethoxyphenyl)-1H-pyrrole-2-carboxylate (**2v**)* The general Suzuki procedure A was applied to methyl 5-(4,4,5,5-tetramethyl-1,3,2-dioxaborolan-2-yl)-1*H*-pyrrole-2-carboxylate (251 mg, 1 mmol, 1 equiv) and 5-bromo-1,2,3-trimethoxybenzene (371 mg, 1.5 mmol, 1.5 equiv) for 48 h. Colorless solid; yield: 246 mg (85%); mp 105–107 °C; *R_f_* = 0.50 (hexanes–CH_2_Cl_2_ 1:3). FT-IR (ATR): 3302, 2995, 2941, 2838, 1677, 1568, 1565, 1476, 1425, 1378, 1238, 1219, 1189, 1042, 999, 927, 832, 700, 662, 613 cm^−1^. ^1^H NMR (700 MHz, CDCl_3_): δ = δ 9.54 (br s, 1 H), 6.95 (dd, *J* = 3.8, 2.4 Hz, 1 H), 6.76 (s, 2 H), 6.47 (dd, *J* = 3.8, 2.7 Hz, 1 H), 3.91 (s, 6 H), 3.87 (s, 3 H), 3.85 (s, 3 H). ^13^C NMR {^1^H} (176 MHz, CDCl_3_): δ = 161.8 (C=O), 153.7 (2 C), 137.9 (C), 137.2 (C), 127.2 (C), 122.8 (C), 116.9 (CH), 108.0 (CH), 102.3 (2 CH), 61.0 (OCH_3_), 56.2 (2 OCH_3_), 51.6 (OCH_3_). GC-MS (EI): *m*/z (%) = 291 (43) (M)^+^, 259 (65), 244 (100), 216 (31), 212 (7), 205 (4), 201 (9), 188 (7), 186 (6), 173 (5), 161 (8), 130 (8). HRMS (ESI-Orbitrap): *m*/*z* [M + H]^+^ calcd for C_15_H_18_NO_5_: 292.11795; found: 292.11762.

*Methyl 5-(3,4,5-trichlorophenyl)-1H-pyrrole-2-carboxylate (**2w**)* The general Suzuki procedure B was applied to methyl 5-(4,4,5,5-tetramethyl-1,3,2-dioxaborolan-2-yl)-1*H*-pyrrole-2-carboxylate (251 mg, 1 mmol, 1 equiv) and 5-bromo-1,2,3-trichlorobenzene (390 mg, 1.5 mmol, 1.5 equiv) for 48 h. Colorless solid; yield: 79 mg (26%); mp 236–238 °C; *R_f_* = 0.1 (hexanes–CH_2_Cl_2_ 1:3). FT-IR (ATR): 3306, 3075, 2954, 1665, 1593, 1566, 1492, 1437, 1334, 1282, 1217, 1090, 1054, 1006, 927, 784, 759, 697 cm^−1^. ^1^H NMR (700 MHz, CDCl_3_): δ = 9.38 (br s, 1 H), 7.56 (s, 2 H), 6.95 (apparent t, *J* = 3.2 Hz, 1 H), 6.56 (apparent t, *J* = 3.5 Hz, 1 H), 3.90 (s, 3 H). ^13^C NMR {^1^H} (176 MHz, CDCl_3_): δ = 161.3 (C=O), 134.9 (2 C), 132.9 (C), 131.3 (C), 130.2 (C), 124.6 (2 CH), 124.4 (C), 116.8 (CH), 109.6 (CH), 51.6 (OCH_3_). GC-MS (EI): *m*/z (%) = 303 (36) (M)^+^, 305 (35) (M+2)^+^, 273 (100), 247 (12), 245 (35), 243 (35), 219 (24), 217 (24), 212 (11), 210 (59), 208 (99), 182 (10), 173 (11). HRMS (ESI-Orbitrap): *m*/*z* [M + H]^+^ calcd for C_12_H_9_Cl_3_NO_2_: 303.96934; found: 303.96995.

*Methyl 5-(anthracen-9-yl)-1H-pyrrole-2-carboxylate (**2x**)* The general Suzuki procedure A was applied to methyl 5-(4,4,5,5-tetramethyl-1,3,2-dioxaborolan-2-yl)-1*H*-pyrrole-2-carboxylate (251 mg, 1 mmol, 1 equiv) and 9-bromoanthracene (386 mg, 1.5 mmol, 1.5 equiv) for 36 h. Light yellow solid; yield: 129 mg (43%); mp 202–207 °C; *R_f_* = 0.50 (hexanes–CH_2_Cl_2_ 1:3). FT-IR (ATR): 3280, 3052, 2951, 2895, 1667, 1555, 1494, 1403, 1212, 1173, 1133, 1042, 963, 931, 890, 851, 740, 656 cm^−1^. ^1^H NMR (700 MHz, CDCl_3_): δ = 9.50 (br s, 1 H), 8.50 (s, 1 H), 8.01 (d, *J* = 8.4, 2 H), 7.82 (dd, *J* = 8.7, 0.7 Hz, 2 H), 7.46 (ddd, *J* = 8.3, 6.5, 1.0 Hz, 2 H), 7.42 (ddd, *J* = 8.6, 6.4, 1.2 Hz, 2 H), 7.16 (dd, *J* = 3.4, 2.7 Hz, 1 H), 6.50 (dd, *J* = 3.6, 2.7 Hz, 1 H), 3.71 (s, 3 H). ^13^C NMR {^1^H} (176 MHz, CDCl_3_): δ = 161.7 (C=O), 133.0 (C), 131.4 (2 C), 131.1 (2 C), 128.4 (2 CH), 128.2 (CH), 126.6 (C), 126.3 (2 CH), 126.1 (2 CH), 125.4 (2 CH), 122.9 (C), 115.9 (CH), 113.6 (CH), 51.4 (OCH_3_). GC-MS (EI): *m*/z (%) = 301 (41) (M)^+^, 269 (85), 241 (100), 216 (16), 120 (2), 108 (2). HRMS (ESI-Orbitrap): *m*/*z* [M + H]^+^ calcd for C_20_H_16_NO_2_: 302.11756; found: 302.11771.

*Methyl 5-(thiophen-2-yl)-1H-pyrrole-2-carboxylate (**3a**)* The general Suzuki procedure A was applied to methyl 5-(4,4,5,5-tetramethyl-1,3,2-dioxaborolan-2-yl)-1*H*-pyrrole-2-carboxylate (251 mg, 1 mmol, 1 equiv) and 2-bromothiophene (145 µL, 245 mg, 1.5 mmol, 1.5 equiv) for 48 h. Yellow solid; yield: 190 mg (92%); mp 109 °C, lit[39] 105–106 °C; *R_f_* = 0.30 (hexanes–CH_2_Cl_2_ 1:3). FT-IR (ATR): 3303, 3101, 3071, 2945, 2841, 1687, 1522, 1477, 1321, 1267, 1140, 1085, 887, 753 cm^−1^. ^1^H NMR (700 MHz, CDCl_3_): δ = 9.47 (br s, 1 H), 7.25 (d, *J* = 4.0 Hz, 1 H), 7.23 (d, *J* = 4.0 Hz, 1 H), 7.04 (apparent t, *J* = 4.6 Hz, 1 H), 6.92 (apparent t, *J* = 3.2 Hz, 1 H), 6.43 (apparent t, *J* = 3.2 Hz, 1 H), 3.87 (s, 3 H). ^13^C NMR {^1^H} (176 MHz, CDCl_3_): δ = 161.5 (C=O), 134.4 (C), 131.4 (C), 127.8 (CH), 124.6 (CH), 123.1 (CH), 122.6 (C), 116.8 (CH), 108.5 (CH), 51.7 (OCH_3_). GC-MS (EI): *m*/z (%) = 207 (100) (M)^+^, 175 (84), 147 (58), 121 (12), 103 (6), 93 (6), 77 (3). HRMS (ESI-Orbitrap): *m*/*z* [M + H]^+^ calcd for C_10_H_10_NO_2_S: 208.04268; found: 208.04268.

*Methyl 5-(5-formylthiophen-2-yl)-1H-pyrrole-2-carboxylate (**3b**)* The general Suzuki procedure A was applied to methyl 5-(4,4,5,5-tetramethyl-1,3,2-dioxaborolan-2-yl)-1*H*-pyrrole-2-carboxylate (251 mg, 1 mmol, 1 equiv) and 5-bromothiophene-2-carbaldehyde (178 µL, 287 mg, 1.5 mmol, 1.5 equiv) for 48 h. Yellow solid; yield: 181 mg (77%); mp 227–230 °C; *R_f_* = 0.20 (hexanes–CH_2_Cl_2_ 1:3). FT-IR (ATR): 3312, 2824, 2795, 1692, 1649, 1575, 1492, 1436, 1375, 1300, 1228, 1148, 1075, 998, 818 cm^−1^. ^1^H NMR (700 MHz, CDCl_3_): δ = 9.89 (s, 1 H), 9.29 (br s, 1 H), 7.71 (d, *J* = 4.0 Hz, 1 H), 7.27 (d, *J* = 4.0 Hz, 1 H), 6.94 (apparent t, *J* = 2.4 Hz, 1 H), 6.61 (apparent t, *J* = 3.1 Hz, 1 H), 3.90 (s, 3 H). ^13^C NMR {^1^H} (176 MHz, CDCl_3_): δ = 182.4 (C=O), 161.1 (C=O), 143.6 (C), 141.8 (CH), 137.3 (CH), 129.6 (C), 124.5 (C), 123.3 (CH), 116.9 (CH), 110.9 (CH), 51.9 (OCH_3_). GC-MS (EI): *m*/z (%) = 235 (92) (M)^+^, 203 (100), 175 (37), 146 (24), 121 (9), 103 (4). HRMS (ESI-Orbitrap): *m*/*z* [M + H]^+^ calcd for C_11_H_10_NO_3_S: 236.03759; found: 236.03645.

*Methyl 5-(5-methylthiophen-3-yl)-1H-pyrrole-2-carboxylate (**3c**)* The general Suzuki procedure B was applied to methyl 5-(4,4,5,5-tetramethyl-1,3,2-dioxaborolan-2-yl)-1*H*-pyrrole-2-carboxylate (251 mg, 1 mmol, 1 equiv) and 4-bromo-2-methylthiophene (168 µL, 266 mg, 1.5 mmol, 1.5 equiv) for 48 h. Colorless solid; yield: 190 mg (86%); mp 173–176 °C; *R_f_* = 0.30 (hexanes–CH_2_Cl_2_ 1:3). FT-IR (ATR): 3315, 3106, 2948, 2873, 1684, 1501, 1436, 1347, 1269, 1237, 1140, 1051, 1003, 929, 875, 786 cm^−1^. ^1^H NMR (700 MHz, CDCl_3_): δ = 9.47 (br s, 1 H), 7.18 (d, *J* = 1.0 Hz, 1 H), 6.98 (s, 1 H), 6.92 (apparent t, *J* = 3.1 Hz, 1 H), 6.36 (apparent, *J* = 3.2 Hz, 1 H), 3.87 (s, 3 H), 2.50 (s, 3 H). ^13^C NMR {^1^H} (176 MHz, CDCl_3_): δ = 161.8 (C=O), 141.0 (C), 133.4 (C), 132.6 (C), 123.5 (CH), 121.9 (C), 117.3 (CH), 116.7 (CH), 107.8 (CH), 51.6 (OCH_3_), 15.3 (CH_3_). GC-MS (EI): *m*/z (%) = 221 (100) (M)^+^, 189 (86), 161 (40), 146 (3), 135 (11), 128 (4), 116 (3), 91 (2), 89 (2). HRMS (ESI-Orbitrap): *m*/*z* [M + H]^+^ calcd for C_11_H_12_NO_2_S: 222.05833; found: 222.05805.

*Dimethyl 1H,1’H-[2,3’-bipyrrole]-5,5’-dicarboxylate (**3d**)* The general Suzuki procedure A was applied to methyl 5-(4,4,5,5-tetramethyl-1,3,2-dioxaborolan-2-yl)-1*H*-pyrrole-2-carboxylate (251 mg, 1 mmol, 1 equiv) and methyl 4-bromo-1*H*-pyrrole-2-carboxylate (306 mg, 1.5 mmol, 1.5 equiv) for 48 h. Yellow solid; yield: 185 mg (75%); mp 203–206 °C; *R_f_* = 0.20 (hexanes–CH_2_Cl_2_ 1:3). FT-IR (ATR): 3320, 3291, 3134, 3001, 2950, 1680, 1610, 1549, 1513, 1442, 1359, 1192, 1069, 1008, 929, 754 cm^−1^. ^1^H NMR (700 MHz, CDCl_3_): δ = 9.23 (br s, 1 H), 9.15 (br s, 1 H), 7.18 (s, 1 H), 7.08 (s, 1 H), 6.92 (s, 1 H), 6.30 (apparent t, *J* = 2.9 Hz, 1 H), 3.89 (s, 3 H), 3.87 (s, 3 H). ^13^C NMR {^1^H} (176 MHz, CDCl_3_): δ = 161.6 (C=O), 161.2 (C=O), 132.0 (C), 123.6 (C), 121.6 (C), 119.2 (CH), 118.2 (C), 116.8 (CH), 111.7 (CH), 106.9 (CH), 51.8 (OCH_3_), 51.5 (OCH_3_). GC-MS (EI): *m*/z (%) = 248 (72) (M)^+^, 216 (100), 184 (75), 156 (13), 129 (12), 102 (6). HRMS (ESI-Orbitrap): *m*/*z* [M + H]^+^ calcd for C_12_H_13_N_2_O_4_: 249.08698; found: 249.08610.

*Methyl 5-(pyridin-3-yl)-1H-pyrrole-2-carboxylate (**3e**)* The general Suzuki procedure A was applied to methyl 5-(4,4,5,5-tetramethyl-1,3,2-dioxaborolan-2-yl)-1*H*-pyrrole-2-carboxylate (251 mg, 1 mmol, 1 equiv) and 3-bromopyridine (145 µL, 237 mg, 1.5 mmol, 1.5 equiv) for 48 h. Colorless solid; yield: 155 mg (77%); mp 149–150 °C, lit[38] 147.9–149.4 °C; *R_f_* = 0.20 (hexanes–EtOAc 2:1). FT-IR (ATR): 3312, 2952, 1683, 1561, 1469, 1333, 1276, 1194, 1121, 1076, 756 cm^−1^. ^1^H NMR (700 MHz, CDCl_3_): δ = 10.57 (br s, 1 H), 8.98 (d, *J* = 1.6 Hz, 1 H), 8.54 (d, *J* = 3.9 Hz, 1 H), 7.95 (d, *J* = 7.8 Hz, 1 H), 7.32 (dd, *J* = 7.8, 4.8 Hz, 1 H), 6.99 (apparent t, *J* = 3.4 Hz, 1 H), 6.60 (apparent t, *J* = 3.1 Hz, 1 H), 3.89 (s, 3 H). ^13^C NMR {^1^H} (176 MHz, CDCl_3_): δ = 162.0 (C=O), 148.5 (CH), 146.6 (CH), 134.0 (C), 132.3 (CH), 127.6 (C), 124.1 (C), 123.6 (CH), 117.1 (CH), 108.9 (CH), 51.9 (OCH_3_). GC-MS (EI): *m*/z (%) = 202 (100) (M)^+^, 170 (80), 142 (50), 115 (30), 89 (11), 63 (5). HRMS (ESI-Orbitrap): *m*/*z* [M + H]^+^ calcd for C_11_H_11_N_2_O_2_: 203.08150; found: 203.08051.

## 4. Conclusions

In conclusion, methyl 5-(4,4,5,5-tetramethyl-1,3,2-dioxaborolan-2-yl)-1*H*-pyrrole-2-carboxylate **1** was synthesized on 10-gram scale using iridium-catalyzed C–H borylation. The borylated pyrrole was successfully employed in Suzuki coupling reactions to prepare a variety of 5-(hetero)aryl substituted pyrrole-2-carboxylates. This catalytic borylation–Suzuki coupling synthetic route has several advantages over direct arylation protocols including compatibility with NH_2_, OH, and pyrrole N-H functional groups, retention of chloro substituents for further functionalization, installation of highly electron-rich aromatic rings, and preparation of bi-heteroaryls including α-β linked bi-pyrrole.

## Supplementary Materials

The following are available online at https://www.mdpi.com/1420-3049/25/9/2106/s1, ^1^H and ^13^C NMR spectra of the synthesized compounds.

## References

[1] Domagala A., Jarosz T., Lapkowski M. (2015). Living on pyrrolic foundations – Advances in natural and artificial bioactive pyrrole derivatives. Eur. J. Med. Chem..

[2] Gholap S.S. (2016). Pyrrole: An emerging scaffold for construction of valuable therapeutic agents. Eur. J. Med. Chem..

[3] Fukuda T., Umeki T., Tokushima K., Xiang G., Yoshida Y., Ishibashi F., Oku Y., Nishiya N., Uehara Y., Iwao M. (2017). Design, synthesis, and evaluation of A-ring-modified lamellarin N analogues as noncovalent inhibitors of the EGFR T790M/L858R mutant. Bioorg. Med. Chem..

[4] Lade D.M., Pawar A.B., Mainkar P.S., Chandrasekhar S. (2017). Total Synthesis of Lamellarin D Trimethyl Ether, Lamellarin, D., and Lamellarin, H.J. Org. Chem..

[5] Imbri D., Tauber J., Opatz T. (2014). Synthetic Approaches to the Lamellarins—A Comprehensive Review. Mar. Drugs.

[6] Dialer C., Imbri D., Hansen S.P., Opatz T. (2015). Synthesis of Lamellarin D Trimethyl Ether and Lamellarin H via 6π-Electrocyclization. J. Org. Chem..

[7] Fan H., Peng J., Hamann M.T., Hu J.-F. (2008). Lamellarins and Related Pyrrole-Derived Alkaloids from Marine Organisms. Chem. Rev..

[8] Saleem R.S.Z., Lansdell T.A., Tepe J.J. (2012). Synthesis and evaluation of debromohymenialdisine-derived Chk2 inhibitors. Bioorg. Med. Chem..

[9] Nguyen T.N.T., Saleem R.S.Z., Luderer M.J., Hovde S., Henry R.W., Tepe J.J. (2012). Radioprotection by Hymenialdisine-Derived Checkpoint Kinase 2 Inhibitors. ACS Chem. Biol..

[10] Curreli F., Belov D.S., Kwon Y.D., Ramesh R., Furimsky A.M., O’Loughlin K., Byrge P.C., Iyer L.V., Mirsalis J.C., Kurkin A.V. (2018). Structure-based lead optimization to improve antiviral potency and ADMET properties of phenyl-1H-pyrrole-carboxamide entry inhibitors targeted to HIV-1 gp120. Eur. J. Med. Chem..

[11] Curreli F., Kwon Y.D., Belov D.S., Ramesh R.R., Kurkin A.V., Altieri A., Kwong P.D., Debnath A.K. (2017). Synthesis, Antiviral Potency, in Vitro ADMET, and X-ray Structure of Potent CD4 Mimics as Entry Inhibitors That Target the Phe43 Cavity of HIV-1 gp120. J. Med. Chem..

[12] Curreli F., Belov D.S., Ramesh R.R., Patel N., Altieri A., Kurkin A.V., Debnath A.K. (2016). Design, synthesis and evaluation of small molecule CD4-mimics as entry inhibitors possessing broad spectrum anti-HIV-1 activity. Bioorg. Med. Chem..

[13] Curreli F., Kwon Y.D., Zhang H., Scacalossi D., Belov D.S., Tikhonov A.A., Andreev I.A., Altieri A., Kurkin A.V., Kwong P.D. (2015). Structure-Based Design of a Small Molecule CD4-Antagonist with Broad Spectrum Anti-HIV-1 Activity. J. Med. Chem..

[14] Pinna G., Loriga G., Murineddu G., Grella G., Mura M., Vargiu L., Murgioni C., La Colla P. (2001). Synthesis and Anti-HIV-1 Activity of New Delavirdine Analogues Carrying Arylpyrrole Moieties. Chem. Pharm. Bull..

[15] Kudryavtsev K.V., Bentley M.L., McCafferty D.G. (2009). Probing of the cis-5-phenyl proline scaffold as a platform for the synthesis of mechanism-based inhibitors of the Staphylococcus aureus sortase SrtA isoform. Bioorg. Med. Chem..

[16] Banwell M.G., Hamel E., Hockless D.C.R., Verdier-Pinard P., Willis A.C., Wong D.J. (2006). 4,5-Diaryl-1H-pyrrole-2-carboxylates as combretastatin A-4/lamellarin T hybrids: Synthesis and evaluation as anti-mitotic and cytotoxic agents. Bioorg. Med. Chem..

[17] Galenko E.E., Galenko A.V., Novikov M.S., Khlebnikov A.F., Kudryavtsev I.V., Terpilowski M.A., Serebriakova M.K., Trulioff A.S., Goncharov N.V. (2017). 4-Diazo and 4-(Triaz-1-en-1-yl)-1H-pyrrole-2-carboxylates as Agents Inducing Apoptosis. ChemistrySelect.

[18] Galenko E.E., Galenko A.V., Khlebnikov A.F., Novikov M.S., Shakirova J.R. (2016). Synthesis and Intramolecular Azo Coupling of 4-Diazopyrrole-2-carboxylates: Selective Approach to Benzo and Hetero [c]-Fused 6H-Pyrrolo[3,4-c]pyridazine-5-carboxylates. J. Org. Chem..

[19] Killoran J., Gallagher J.F., Murphy P.V., O’Shea D.F. (2005). A study of the effects of subunit pre-orientation for diarylpyrrole esters; design of new aryl-heteroaryl fluorescent sensors. New J. Chem..

[20] Granda J.M., Staszewska-Krajewska O., Jurczak J. (2014). Bispyrrolylbenzene Anion Receptor: From Supramolecular Switch to Molecular Logic Gate. Chem. Eur. J..

[21] Zhang H., Lee J., Lammer A.D., Chi X., Brewster J.T., Lynch V.M., Li H., Zhang Z., Sessler J.L. (2016). Self-Assembled Pyridine-Dipyrrolate Cages. J. Am. Chem. Soc..

[22] Chaolu E., Satoshi H., Jun-ichiro S. (2015). One-Handed Single Helicates of Dinickel(II) Benzenehexapyrrole-α,ω-diimine with an Amine Chiral Source. Chem. Eur. J..

[23] Setsune J.-i., Kawama M., Nishinaka T. (2011). Helical binuclear CoII complexes of pyriporphyrin analogue for sensing homochiral carboxylic acids. Tetrahedron Lett..

[24] Boukou-Poba J.P., Farnier M., Guilard R. (1979). A general method for the synthesis of 2-arylpyrroles. Tetrahedron Lett..

[25] Ezquerra J., Pedregal C., Rubio A., Valenciano J., Navio J.L.G., Alvarez-Builla J., Vaquero J.J. (1993). General method for the synthesis of 5-arylpyrrole-2-carboxylic acids. Tetrahedron Lett..

[26] Queiroz M.-J.R.P., Begouin A., Pereira G., Ferreira P.M.T. (2008). New synthesis of methyl 5-aryl or heteroaryl pyrrole-2-carboxylates by a tandem Sonogashira coupling/5-endo-dig-cyclization from β-iododehydroamino acid methyl esters and terminal alkynes. Tetrahedron.

[27] Estevez V., Villacampa M., Menendez J.C. (2014). Recent advances in the synthesis of pyrroles by multicomponent reactions. Chem. Soc. Rev..

[28] Cheng B.-Y., Wang Y.-N., Li T.-R., Lu L.-Q., Xiao W.-J. (2017). Synthesis of Polysubstituted Pyrroles through a Formal [4 + 1] Cycloaddition/E1cb Elimination/Aromatization Sequence of Sulfur Ylides and α,β-Unsaturated Imines. J. Org. Chem..

[29] Ngwerume S., Lewis W., Camp J.E. (2013). Development of a Gold-Multifaceted Catalysis Approach to the Synthesis of Highly Substituted Pyrroles: Mechanistic Insights via Huisgen Cycloaddition Studies. J. Org. Chem..

[30] Wang Z., Shi Y., Luo X., Han D.-M., Deng W.-P. (2013). Direct synthesis of pyrroles via 1,3-dipolar cycloaddition of azomethine ylides with ynones. New J. Chem..

[31] Kudryavtsev K.V., Ivantcova P.M., Churakov A.V., Vasin V.A. (2012). Phenyl α-bromovinyl sulfone in cycloadditions with azomethine ylides: An unexpected facile aromatization of the cycloadducts into pyrroles. Tetrahedron Lett..

[32] Lade D.M., Pawar A.B. (2016). Cp*Co(iii)-catalyzed vinylic C-H bond activation under mild conditions: Expedient pyrrole synthesis via (3 + 2) annulation of enamides and alkynes. Org. Chem. Front..

[33] Imbri D., Netz N., Kucukdisli M., Kammer L.M., Jung P., Kretzschmann A., Opatz T. (2014). One-Pot Synthesis of Pyrrole-2-carboxylates and -carboxamides via an Electrocyclization/Oxidation Sequence. J. Org. Chem..

[34] López-Pérez A., Robles-Machín R., Adrio J., Carretero J.C. (2007). Oligopyrrole Synthesis by 1,3-Dipolar Cycloaddition of Azomethine Ylides with Bissulfonyl Ethylenes. Angew. Chem. Int. Ed..

[35] Wang Y., Jiang C.-M., Li H.-L., He F.-S., Luo X., Deng W.-P. (2016). Regioselective Iodine-Catalyzed Construction of Polysubstituted Pyrroles from Allenes and Enamines. J. Org. Chem..

[36] Galenko E.E., Bodunov V.A., Galenko A.V., Novikov M.S., Khlebnikov A.F. (2017). Fe(II)-Catalyzed Isomerization of 4-Vinylisoxazoles into Pyrroles. J. Org. Chem..

[37] Padwa A., Stengel T. (2004). Grubbs and Wilkinson catalyzed reactions of 2-phenyl-3-vinyl substituted 2H-azirines. Arkivoc.

[38] Farney E.P., Yoon T.P. (2014). Visible-Light Sensitization of Vinyl Azides by Transition-Metal Photocatalysis. Angew. Chem. Int. Ed..

[39] Dong H., Shen M., Redford J.E., Stokes B.J., Pumphrey A.L., Driver T.G. (2007). Transition Metal-Catalyzed Synthesis of Pyrroles from Dienyl Azides. Org. Lett..

[40] Liu C., Ji C.L., Hong X., Szostak M. (2018). Palladium-Catalyzed Decarbonylative Borylation of Carboxylic Acids: Tuning Reaction Selectivity by Computation. Angew. Chem. Int. Ed..

[41] Akira S., Yasunori Y. (2011). Cross-coupling Reactions of Organoboranes: An Easy Method for C–C Bonding. Chem. Lett..

[42] Lennox A.J.J., Lloyd-Jones G.C. (2014). Selection of boron reagents for Suzuki–Miyaura coupling. Chem. Soc. Rev..

[43] El-Maiss J., Mohy El Dine T., Lu C.-S., Karamé I., Kanj A., Polychronopoulou K., Shaya J. (2020). Recent Advances in Metal-Catalyzed Alkyl–Boron (C(sp^3^)–C(sp^2^)) Suzuki-Miyaura Cross-Couplings. Catalysts.

[44] Shi S., Szostak M. (2019). Decarbonylative Borylation of Amides by Palladium Catalysis. ACS Omega.

[45] Martina S., Enkelmann V., Wegner G., Schlüter A.-D. (1991). N-Protected Pyrrole Derivatives Substituted for Metal-Catalyzed Cross-Coupling Reactions. Synthesis.

[46] Laha J.K., Sharma S., Bhimpuria R.A., Dayal N., Dubey G., Bharatam P.V. (2017). Integration of oxidative arylation with sulfonyl migration: One-pot tandem synthesis of densely functionalized (NH)-pyrroles. New J. Chem..

[47] Yiğit B., Gürbüz N., Yiğit M., Dağdeviren Z., Özdemir İ. (2017). Palladium(II) N-heterocyclic carbene complexes as catalysts for the direct arylation of pyrrole derivatives with aryl chlorides. Inorg. Chim. Acta.

[48] Laha J.K., Bhimpuria R.A., Prajapati D.V., Dayal N., Sharma S. (2016). Palladium-catalyzed regioselective C-2 arylation of 7-azaindoles, indoles, and pyrroles with arenes. Chem. Commun..

[49] Carina S., Karthik D., Andreas O., Gates P.J., Pilarski L.T. (2015). Ru-Catalysed C-H Arylation of Indoles and Pyrroles with Boronic Acids: Scope and Mechanistic Studies. Chem. Eur. J..

[50] Wang L., Li Z., Qu X., Peng W. (2015). Highly Efficient Synthesis of Arylpyrrole Derivatives via Rh(III)-Catalyzed Direct C-H Arylation with Aryl Boronic Acids. Chin. J. Chem..

[51] Pla D., Marchal A., Olsen C.A., Albericio F., Álvarez M. (2005). Modular Total Synthesis of Lamellarin, D.J. Org. Chem..

[52] Belov D.S., Ivanov V.N., Curreli F., Kurkin A.V., Altieri A., Debnath A.K. (2017). Synthesis of 5-Arylpyrrole-2-carboxylic Acids as Key Intermediates for NBD Series HIV-1 Entry Inhibitors. Synthesis.

[53] Hodge P., Rickards R.W. (1965). 72. The halogenation of methyl pyrrole-2-carboxylate and of some related pyrroles. J. Chem. Soc..

[54] Anderson H.J., Lee S.-F. (1965). Pyrrole Chemistry: IV. The Preparation and some reactions of brominated pyrrole derivatives. Can. J. Chem..

[55] Chen W., Cava M.P. (1987). Convenient synthetic equivalents of 2-lithiopyrrole and 2,5-dilithiopyrrole. Tetrahedron Lett..

[56] Komatsubara M., Umeki T., Fukuda T., Iwao M. (2014). Modular Synthesis of Lamellarins via Regioselective Assembly of 3,4,5-Differentially Arylated Pyrrole-2-carboxylates. J. Org. Chem..

[57] Urbano M., Guerrero M., Zhao J., Velaparthi S., Schaeffer M.-T., Brown S., Rosen H., Roberts E. (2011). SAR analysis of innovative selective small molecule antagonists of sphingosine-1-phosphate 4 (S1P4) receptor. Bioorg. Med. Chem. Lett..

[58] Setsune J.-i., Toda M., Watanabe K., Panda P.K., Yoshida T. (2006). Synthesis of bis(pyrrol-2-yl)arenes by Pd-catalyzed cross coupling. Tetrahedron Lett..

[59] Cho J.-Y., Tse M.K., Holmes D., Maleczka R.E., Smith M.R. (2002). Remarkably Selective Iridium Catalysts for the Elaboration of Aromatic C-H Bonds. Science.

[60] Ishiyama T., Takagi J., Ishida K., Miyaura N., Anastasi N.R., Hartwig J.F. (2002). Mild Iridium-Catalyzed Borylation of Arenes. High Turnover Numbers, Room Temperature Reactions, and Isolation of a Potential Intermediate. J. Am. Chem. Soc..

[61] Mkhalid I.A.I., Barnard J.H., Marder T.B., Murphy J.M., Hartwig J.F. (2010). C−H Activation for the Construction of C−B Bonds. Chem. Rev..

[62] Xu L., Wang G., Zhang S., Wang H., Wang L., Liu L., Jiao J., Li P. (2017). Recent advances in catalytic C−H borylation reactions. Tetrahedron.

[63] Takagi J., Sato K., Hartwig J.F., Ishiyama T., Miyaura N. (2002). Iridium-catalyzed C–H coupling reaction of heteroaromatic compounds with bis(pinacolato)diboron: Regioselective synthesis of heteroarylboronates. Tetrahedron Lett..

[64] Ishiyama T., Nobuta Y., Hartwig J.F., Miyaura N. (2003). Room temperature borylation of arenes and heteroarenes using stoichiometric amounts of pinacolborane catalyzed by iridium complexes in an inert solvent. Chem. Commun..

[65] Greenwood R., Yeung K. (2016). The synthesis of novel pyrrololactams and their boronate ester derivatives. Tetrahedron Lett..

[66] Paul S., Chotana G.A., Holmes D., Reichle R.C., Maleczka R.E., Smith M.R. (2006). Ir-Catalyzed Functionalization of 2-Substituted Indoles at the 7-Position: Nitrogen-Directed Aromatic Borylation. J. Am. Chem. Soc..

[67] Robbins D.W., Boebel T.A., Hartwig J.F. (2010). Iridium-Catalyzed, Silyl-Directed Borylation of Nitrogen-Containing Heterocycles. J. Am. Chem. Soc..

[68] Shen F., Tyagarajan S., Perera D., Krska S.W., Maligres P.E., Smith M.R., Maleczka R.E. (2016). Bismuth Acetate as a Catalyst for the Sequential Protodeboronation of Di- and Triborylated Indoles. Org. Lett..

[69] Eastabrook A.S., Sperry J. (2017). Synthetic Access to 3,5,7-Trisubstituted Indoles Enabled by Iridium Catalyzed C–H Borylation. Synthesis.

[70] Chotana G.A., Kallepalli V.A., Maleczka R.E., Smith M.R. (2008). Iridium-catalyzed borylation of thiophenes: Versatile, synthetic elaboration founded on selective C–H functionalization. Tetrahedron.

[71] Sadler S.A., Tajuddin H., Mkhalid I.A.I., Batsanov A.S., Albesa-Jove D., Cheung M.S., Maxwell A.C., Shukla L., Roberts B., Blakemore D.C. (2014). Iridium-catalyzed C-H borylation of pyridines. Org. Biomol. Chem..

[72] Batool F., Emwas A.-H., Gao X., Munawar M.A., Chotana G.A. (2017). Synthesis and Suzuki Cross-Coupling Reactions of 2,6-Bis(trifluoromethyl)pyridine-4-boronic Acid Pinacol Ester. Synthesis.

[73] Yang L., Semba K., Nakao Y. (2017). para-Selective C−H Borylation of (Hetero)Arenes by Cooperative Iridium/Aluminum Catalysis. Angew. Chem. Int. Ed..

[74] Larsen M.A., Hartwig J.F. (2014). Iridium-Catalyzed C–H Borylation of Heteroarenes: Scope, Regioselectivity, Application to Late-Stage Functionalization, and Mechanism. J. Am. Chem. Soc..

[75] Tse M.K., Cho J.-Y., Smith M.R. (2001). Regioselective Aromatic Borylation in an Inert Solvent. Org. Lett..

[76] Kallepalli V.A., Shi F., Paul S., Onyeozili E.N., Maleczka R.E., Smith M.R. (2009). Boc Groups as Protectors and Directors for Ir-Catalyzed C−H Borylation of Heterocycles. J. Org. Chem..

[77] Swartz D.L., Staples R.J., Odom A.L. (2011). Synthesis and hydroamination catalysis with 3-aryl substituted pyrrolyl and dipyrrolylmethane titanium(iv) complexes. Dalton Trans..

[78] Robbins D.W., Hartwig J.F. (2012). A C–H Borylation Approach to Suzuki–Miyaura Coupling of Typically Unstable 2–Heteroaryl and Polyfluorophenyl Boronates. Org. Lett..

[79] Asghar S., Shahzadi T., Alazmi M., Gao X., Emwas A.-H., Saleem R.S.Z., Batool F., Chotana G.A. (2018). Iridium-Catalyzed Regioselective Borylation of Substituted Biaryls. Synthesis.

[80] Batool F., Parveen S., Emwas A.-H., Sioud S., Gao X., Munawar M.A., Chotana G.A. (2015). Synthesis of Fluoroalkoxy Substituted Arylboronic Esters by Iridium-Catalyzed Aromatic C–H Borylation. Org. Lett..

[81] Shahzadi T., Saleem R.S.Z., Chotana G.A. (2018). Facile Synthesis of Halogen Decorated para-/meta-Hydroxy benzoates by Iridium-Catalyzed Borylation and Oxidation. Synthesis.

[82] Ikram H.M., Rasool N., Ahmad G., Chotana G.A., Musharraf S.G., Zubarir M., Rana U.A., Zia-ul-Haq M., Jaafar H.Z. (2015). Selective C-Arylation of 2,5-Dibromo-3-hexylthiophene via Suzuki Cross Coupling Reaction and Their Pharmacological Aspects. Molecules.

[83] Ikram H.M., Rasool N., Zubair M., Khan K.M., Chotana G.A., Akhtar M.N., Abu N., Alitheen N.B., Elgorban A.M., Rana U.A. (2016). Efficient Double Suzuki Cross-Coupling Reactions of 2,5-Dibromo-3-hexylthiophene: Anti-Tumor, Haemolytic, Anti-Thrombolytic and Biofilm Inhibition Studies. Molecules.

[84] Qazi F., Zakir H., Asghar S., Abbas G., Riaz M. (2018). Malus domestica Mediated Synthesis of Palladium Nanoparticles and Investigation of Their Catalytic Activity Towards the Suzuki Coupling Reactions. Nanosci. Nanotechnol. Lett..

[85] Miller S.L., Chotana G.A., Fritz J.A., Chattopadhyay B., Maleczka R.E., Smith M.R. (2019). C-H Borylation Catalysts that Distinguish between Similarly Sized Substituents like Fluorine and Hydrogen. Org. Lett..

[86] Chotana G.A., Montero Bastidas J.R., Miller S.L., Smith M.R., Maleczka R.E. (2020). One-Pot Iridium-Catalyzed C–H Borylation/Sonogashira Cross-Coupling: Access to Borylated Aryl Alkynes. Molecules.

[87] Ishiyama T., Takagi J., Yonekawa Y., Hartwig J.F., Miyaura N. (2003). Iridium-Catalyzed Direct Borylation of Five-Membered Heteroarenes by Bis(pinacolato)diboron: Regioselective, Stoichiometric, and Room Temperature Reactions. Adv. Synth. Catal..

[88] Billingsley K.L., Anderson K.W., Buchwald S.L. (2006). A Highly Active Catalyst for Suzuki–Miyaura Cross-Coupling Reactions of Heteroaryl Compounds. Angew. Chem. Int. Ed..

[89] Rieth R.D., Mankad N.P., Calimano E., Sadighi J.P. (2004). Palladium-Catalyzed Cross-Coupling of Pyrrole Anions with Aryl Chlorides, Bromides, and Iodides. Org. Lett..

[90] Farnier M., Soth S., Fournari P. (1976). Recherches en série hétérocyclique. XXVIII. Synthèse de bipyrroles. Can. J. Chem..

[91] Castro A.J., Giannini D.D., Greenlee W.F. (1970). Synthesis of a 2,3’-bipyrrole. Denitrosation in the Knorr pyrrole synthesis. J. Org. Chem..

[92] Dohi T., Morimoto K., Maruyama A., Kita Y. (2006). Direct Synthesis of Bipyrroles Using Phenyliodine Bis(trifluoroacetate) with Bromotrimethylsilane. Org. Lett..

