# Supplementary material for: Facile Synthesis of NH-Free 5-(Hetero)Aryl-Pyrrole-2-Carboxylates by Catalytic C–H Borylation and Suzuki Coupling
Source: Molecules. 2020 Apr 30;25(9):2106. doi: 10.3390/molecules25092106 (PMC7248765; doi:10.3390/molecules25092106)
Supplement: Supplementary file 1 [file molecules-25-02106-s001.pdf]

Facile Synthesis of NH-Free 5-(Hetero)Aryl-Pyrrole-2-Carboxylates by Catalytic C–H Borylation and Suzuki Coupling

Saba Kanwal [1], Noor-ul-Ann [1], Saman Fatima [1], Abdul-Hamid Emwas [2], Meshari Alazmi [3,4], Xin Gao [3], Maha Ibrar [1], Rahman Shah Zaib Saleem [1] and Ghayoor Abbas Chotana [1,*]

[1]Department of Chemistry and Chemical Engineering, Syed Babar Ali School of Science & Engineering, Lahore University of Management Sciences, Lahore-54792, Pakistan

[2]King Abdullah University of Science and Technology (KAUST), Core Labs, Thuwal, 23955-6900, Saudi Arabia

[3]King Abdullah University of Science and Technology (KAUST), Computational Bioscience Research Center (CBRC), Computer, Electrical and Mathematical Sciences and Engineering (CEMSE) Division, Thuwal, 23955-6900, Saudi Arabia

[4]College of Computer Science and Engineering, University of Ha'il, P.O. Box 2440, Ha'il, 81481, Saudi Arabia

Email: ghayoor.abbas@lums.edu.pk

Supporting Information

Table of Contents:

- **NMR Spectra S2**

^{1}H & ^{13}C NMR Spectra

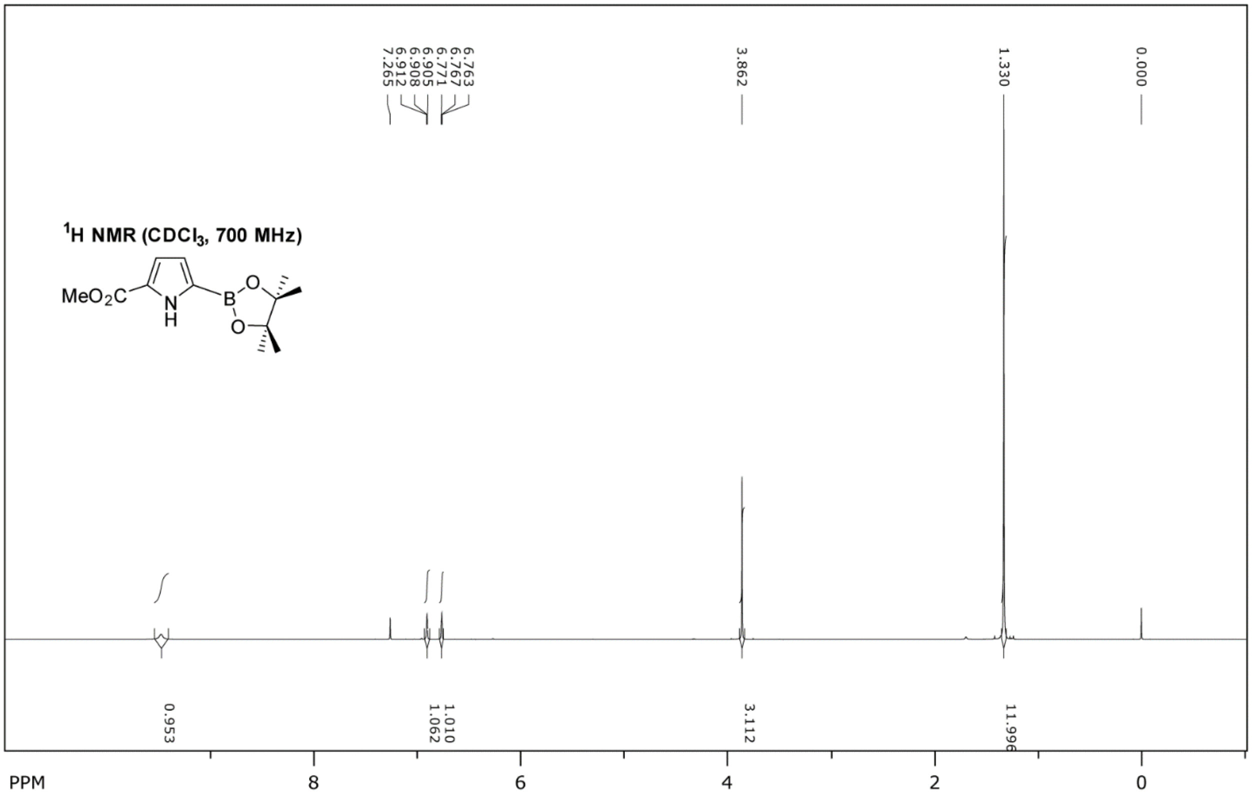

Compound 1: ^{1}H NMR spectrum of methyl 5-(4,4,5,5-tetramethyl-1,3,2-dioxaborolan-2-yl)-1*H*-pyrrole-2-carboxylate

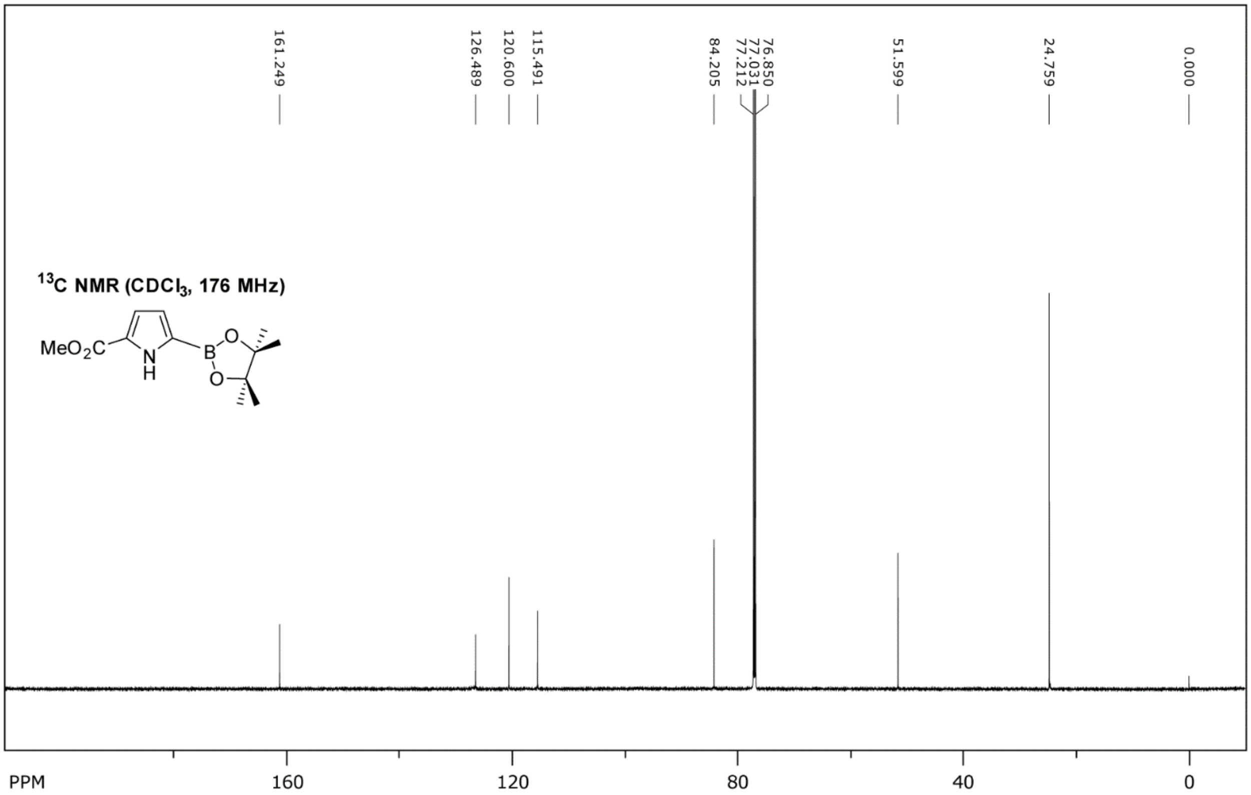

Compound 1: ^{13}C NMR spectrum of methyl 5-(4,4,5,5-tetramethyl-1,3,2-dioxaborolan-2-yl)-1*H*-pyrrole-2-carboxylate

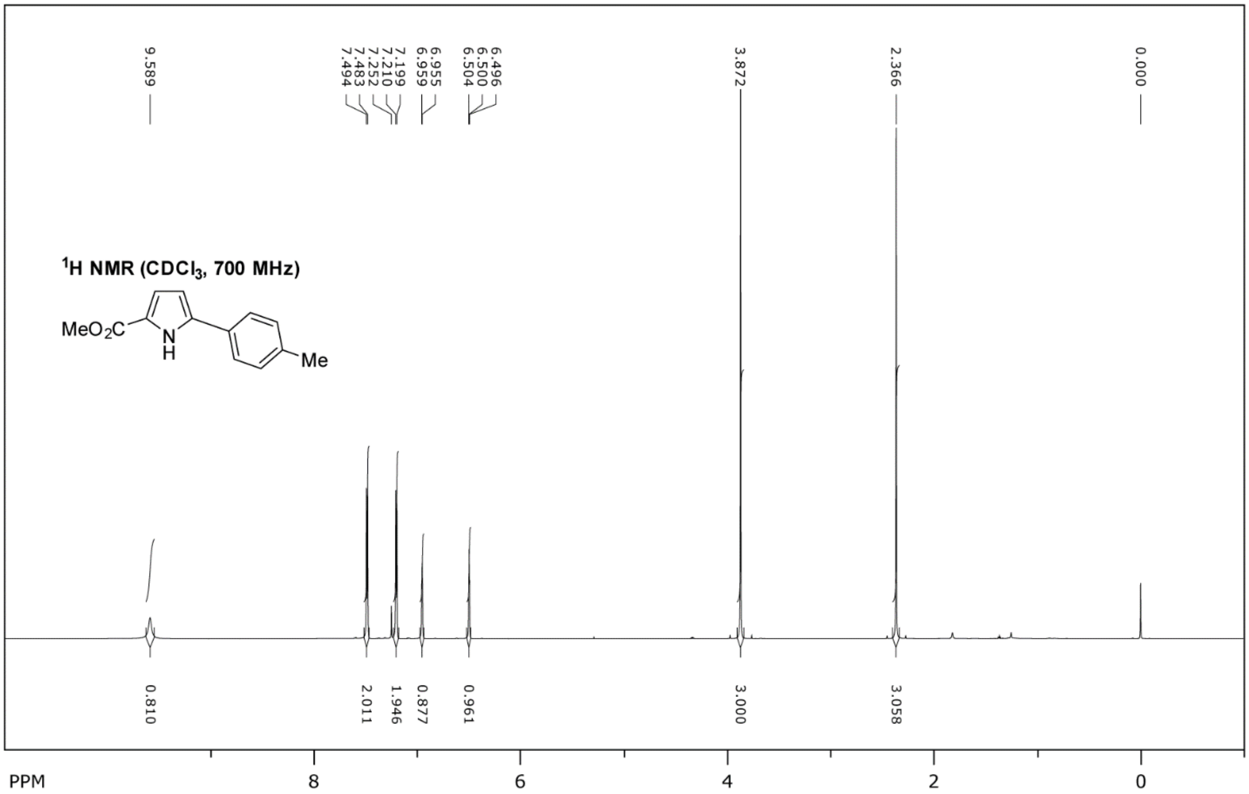

Compound 2a: ^{1}H NMR spectrum of methyl 5-(p-tolyl)-1*H*-pyrrole-2-carboxylate

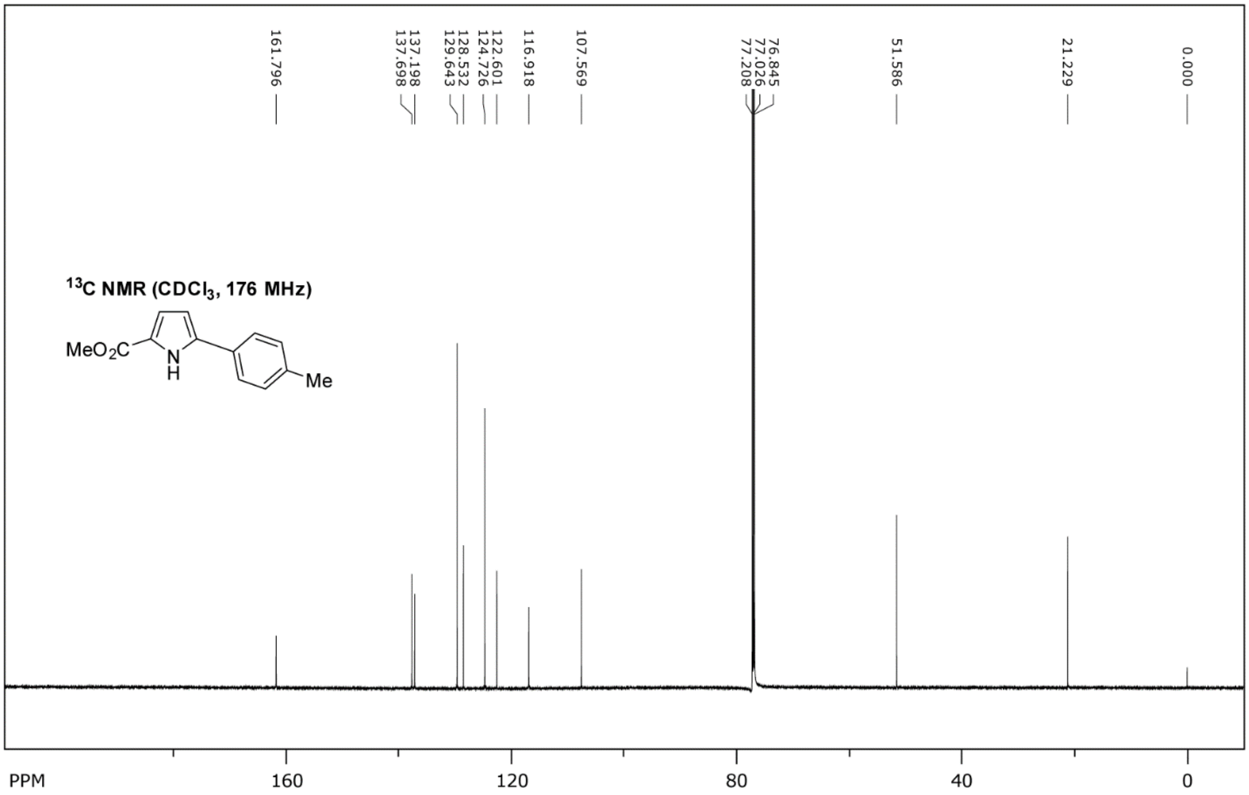

Compound 2a: ^{13}C NMR spectrum of methyl 5-(p-tolyl)-1*H*-pyrrole-2-carboxylate

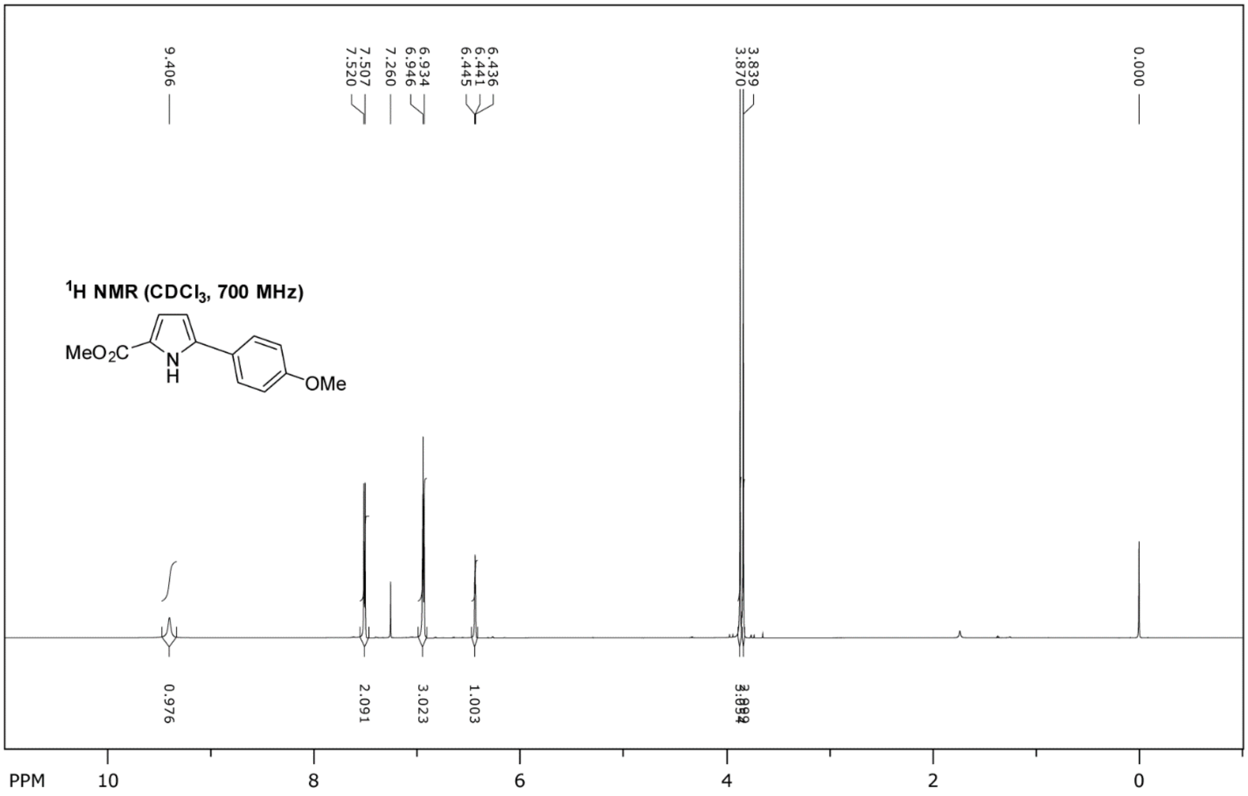

Compound 2b: ^{1}H NMR spectrum of methyl 5-(4-methoxyphenyl)-1*H*-pyrrole-2-carboxylate

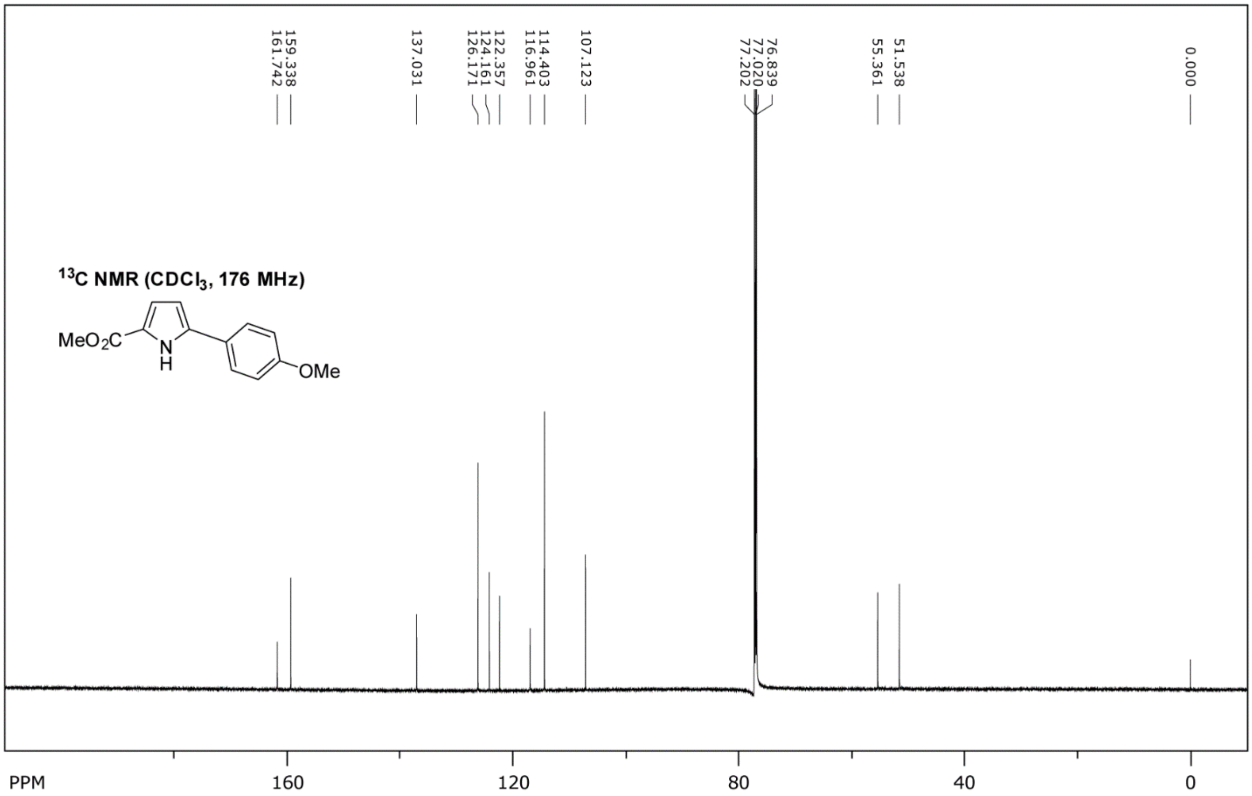

Compound 2b: ^{13}C NMR spectrum of methyl 5-(4-methoxyphenyl)-1*H*-pyrrole-2-carboxylate

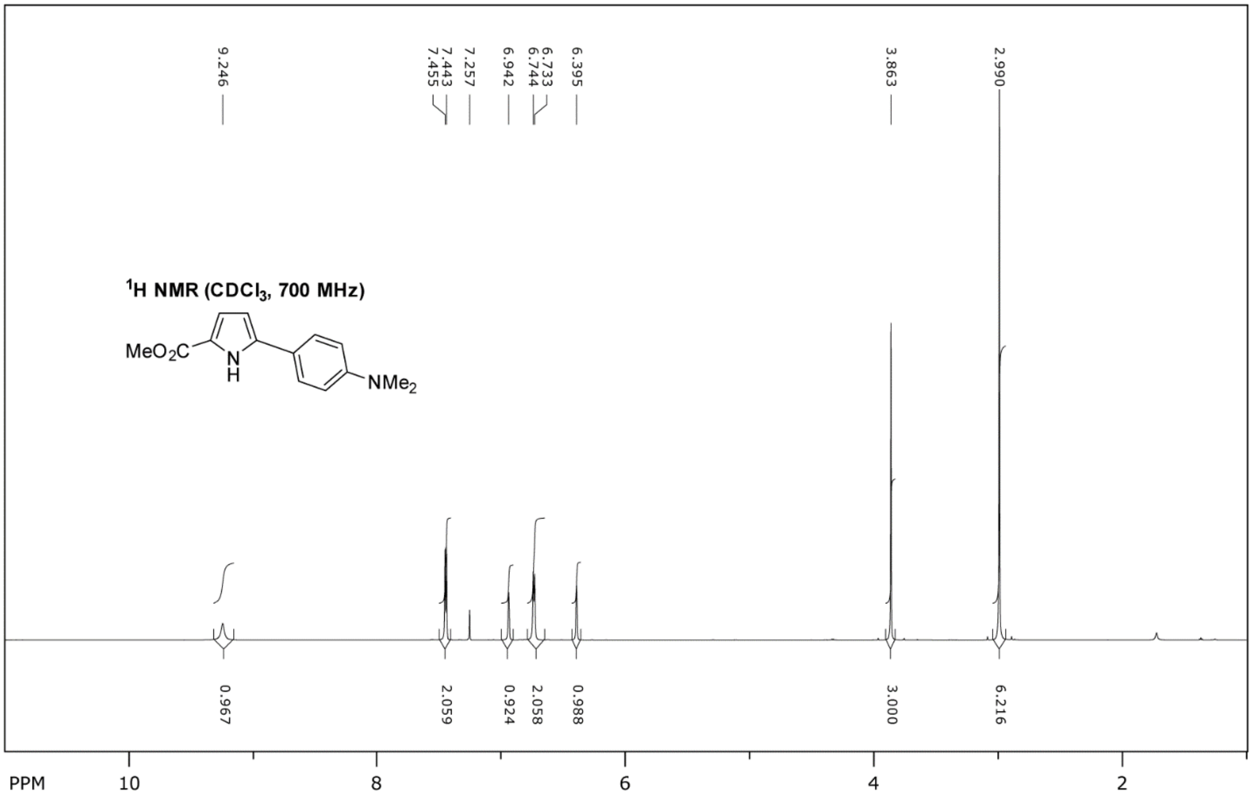

Compound 2c: ^{1}H NMR spectrum of methyl 5-(4-(dimethylamino)phenyl)-1*H*-pyrrole-2-carboxylate

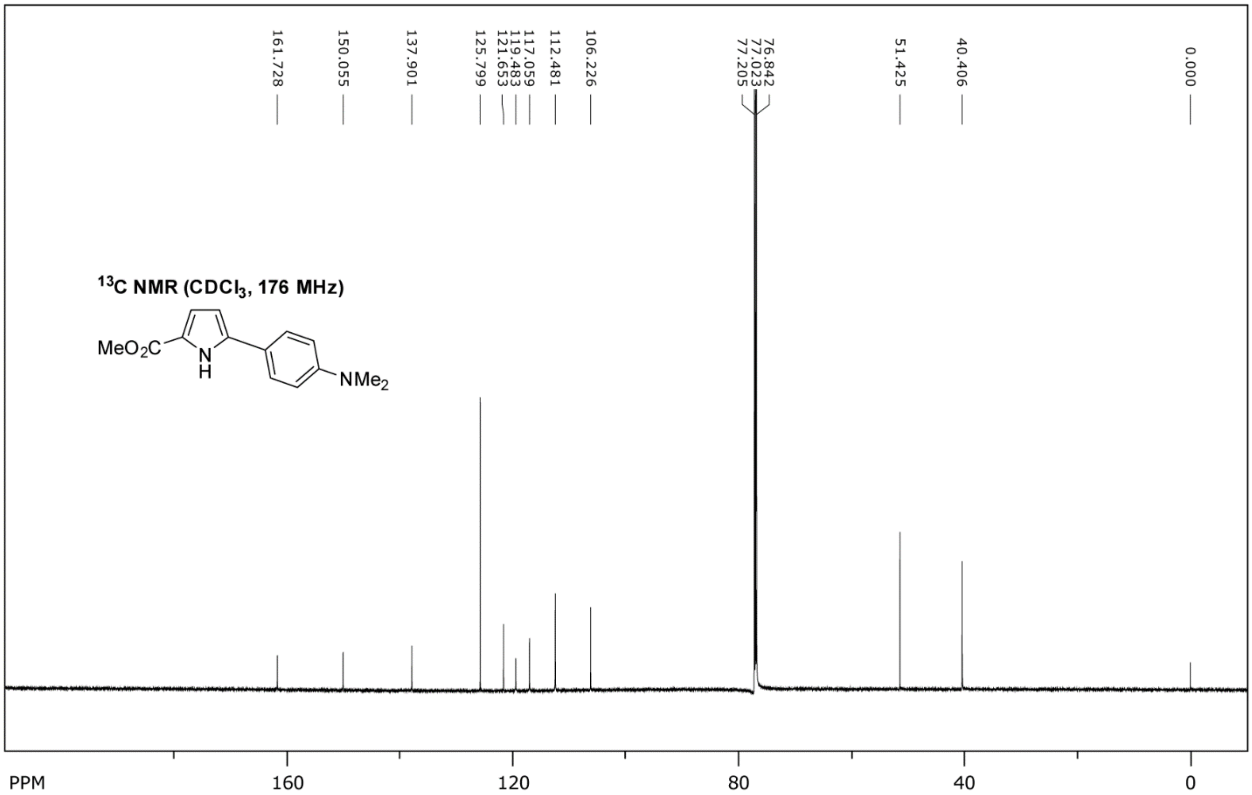

Compound 2c: ^{13}C NMR spectrum of methyl 5-(4-(dimethylamino)phenyl)-1*H*-pyrrole-2-carboxylate

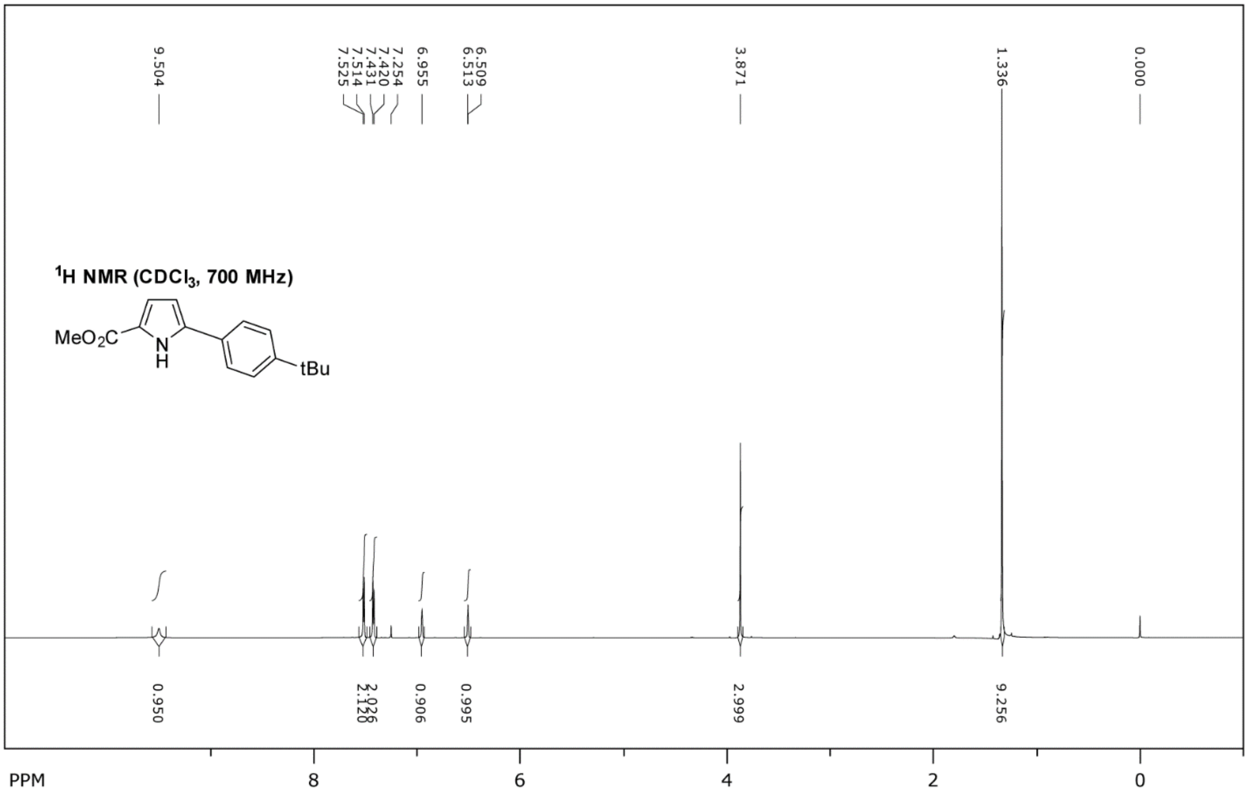

Compound 2d: ^{1}H NMR spectrum of methyl 5-(4-(tert-butyl)phenyl)-1*H*-pyrrole-2-carboxylate

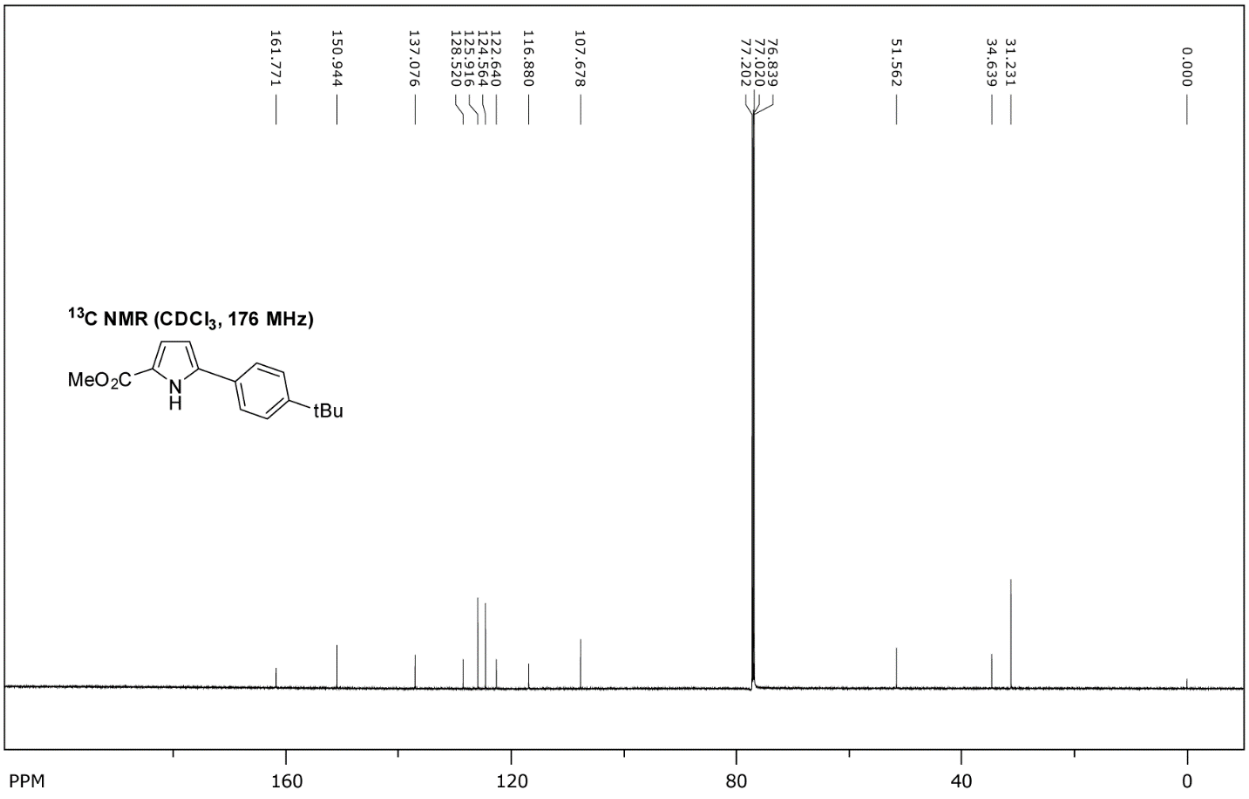

Compound 2d: ^{13}C NMR spectrum of methyl 5-(4-(tert-butyl)phenyl)-1*H*-pyrrole-2-carboxylate

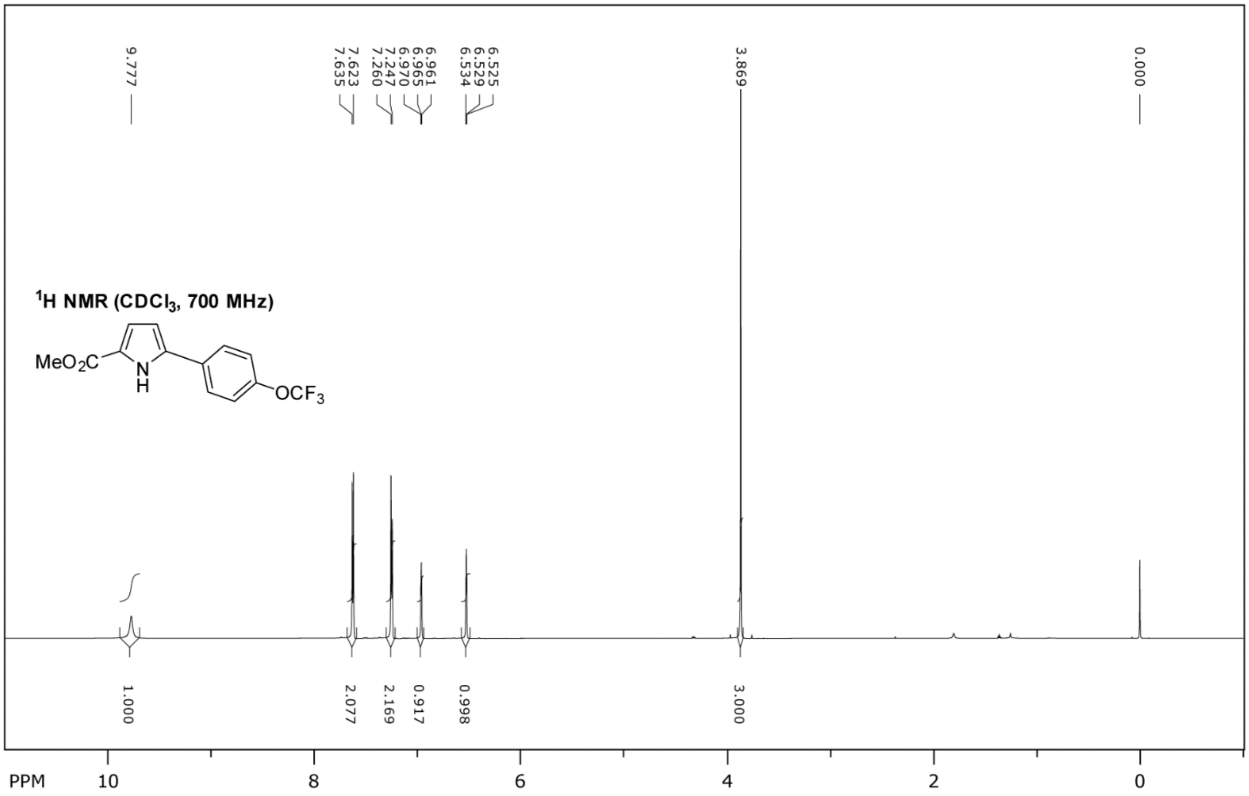

Compound 2e: ^{1}H NMR spectrum of methyl 5-(4-(trifluoromethoxy)phenyl)-1*H*-pyrrole-2-carboxylate

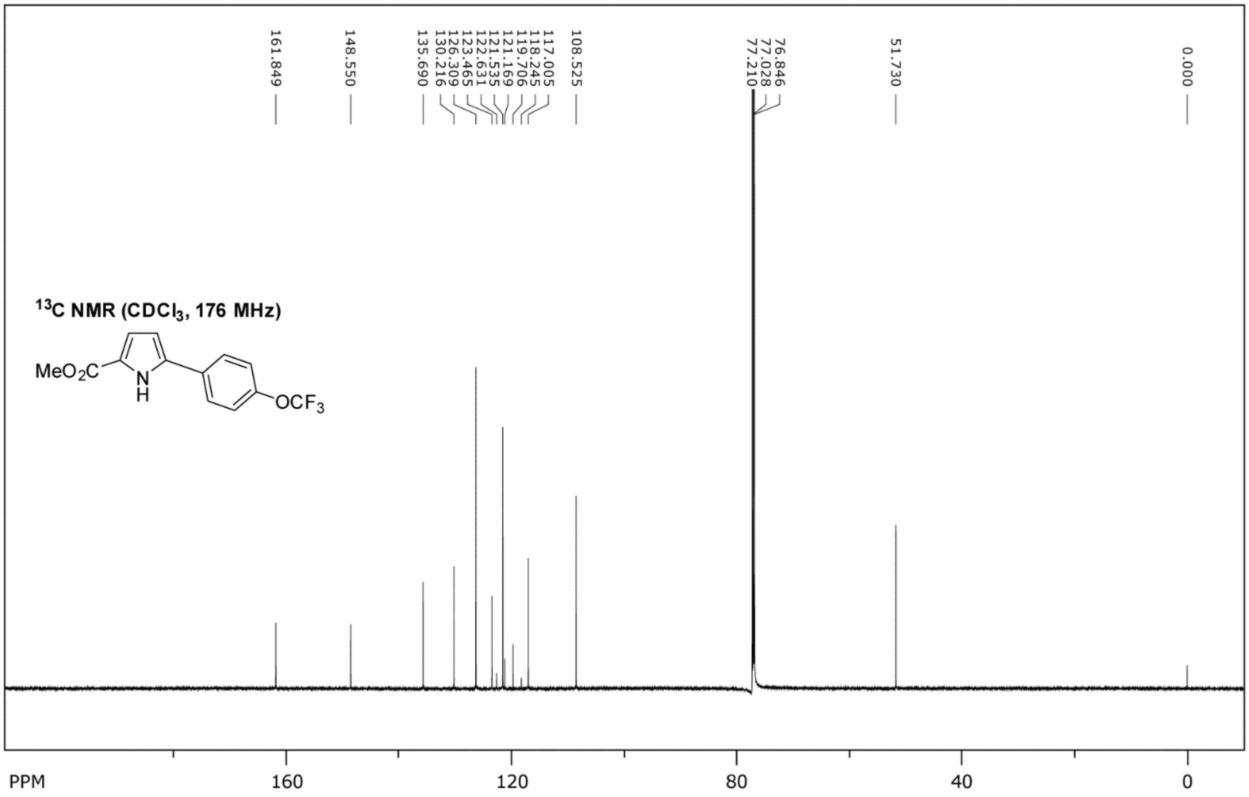

Compound 2e: ^{13}C NMR spectrum of methyl 5-(4-(trifluoromethoxy)phenyl)-1*H*-pyrrole-2-carboxylate

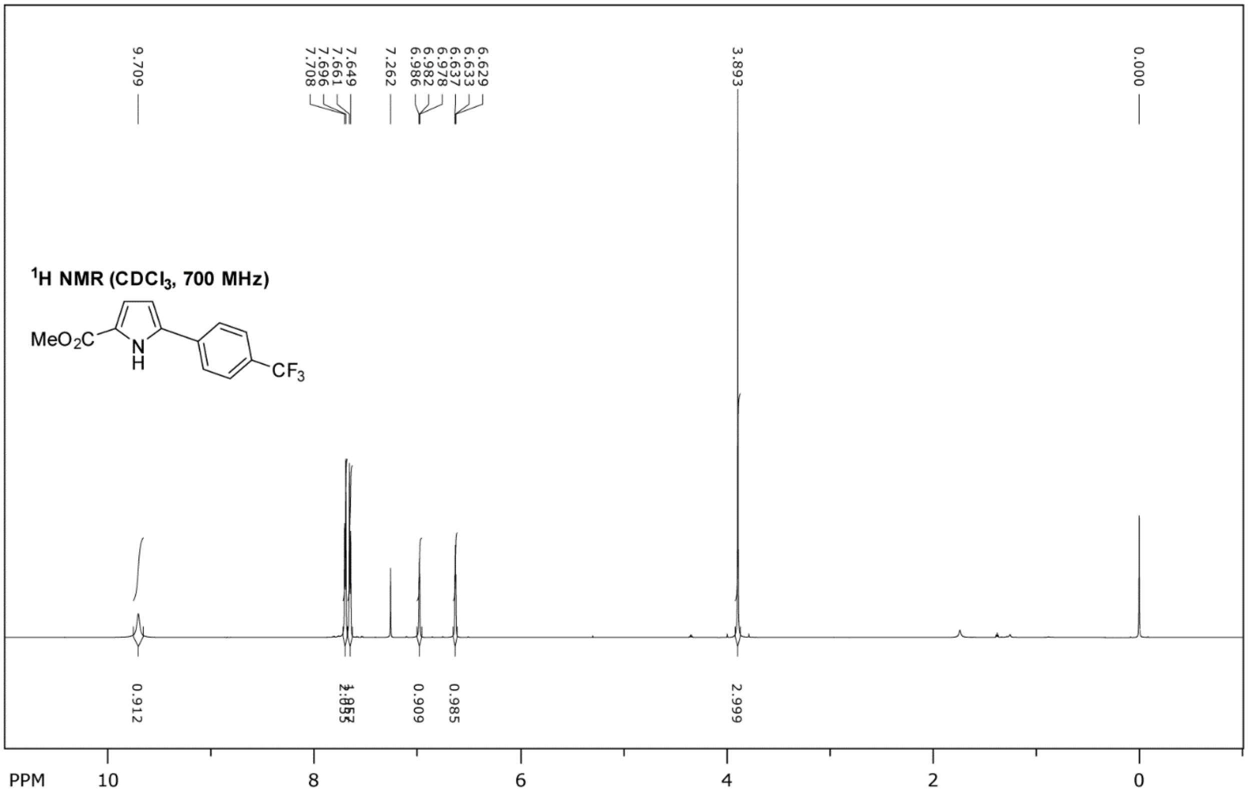

Compound 2f: ^{1}H NMR spectrum of methyl 5-(4-(trifluoromethyl)phenyl)-1*H*-pyrrole-2-carboxylate

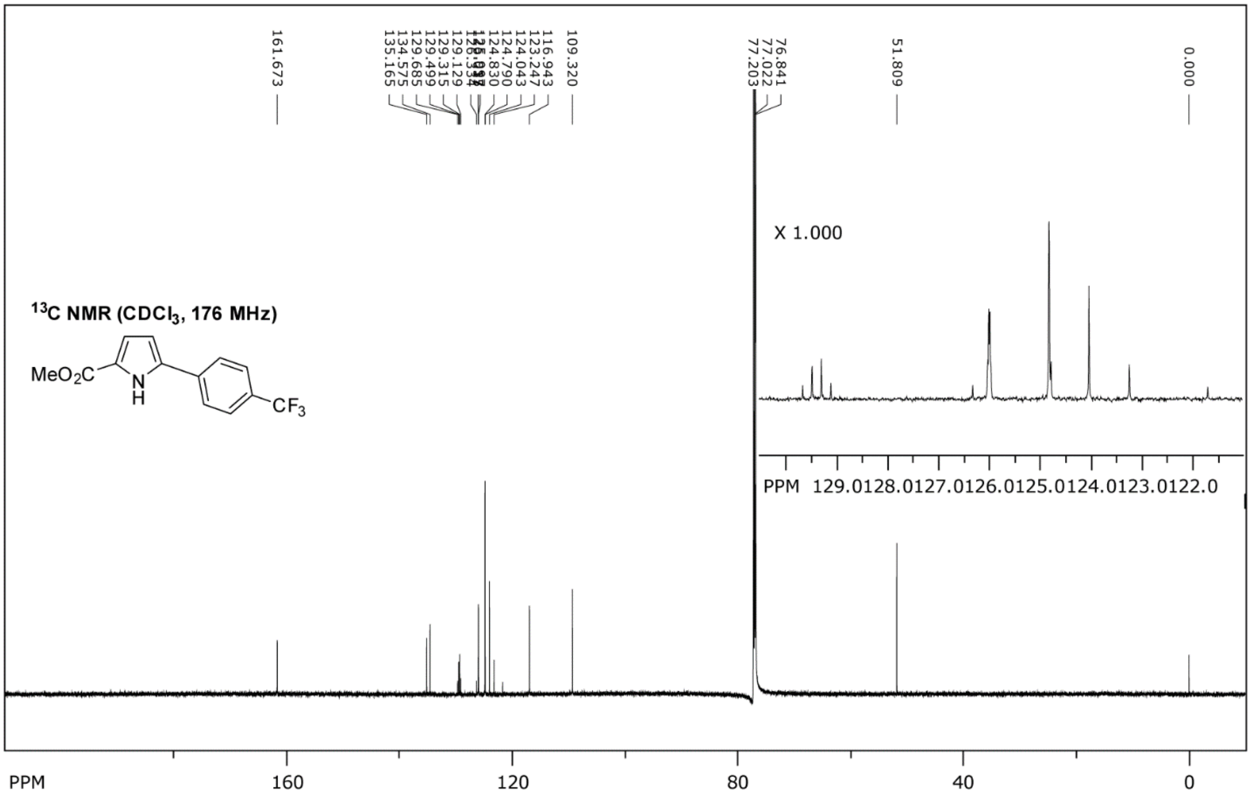

Compound 2f: ^{13}C NMR spectrum of methyl 5-(4-(trifluoromethyl)phenyl)-1*H*-pyrrole-2-carboxylate

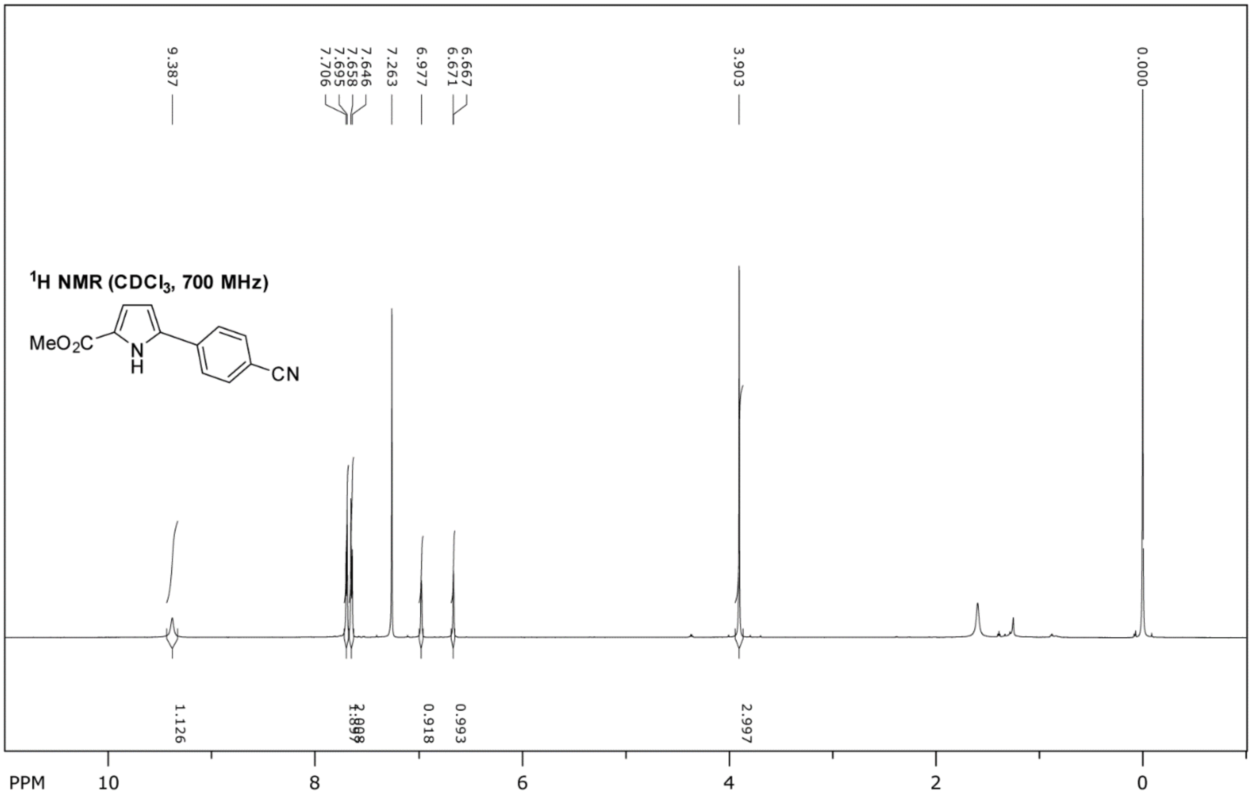

Compound 2g: ^{1}H NMR spectrum of methyl 5-(4-cyanophenyl)-1*H*-pyrrole-2-carboxylate

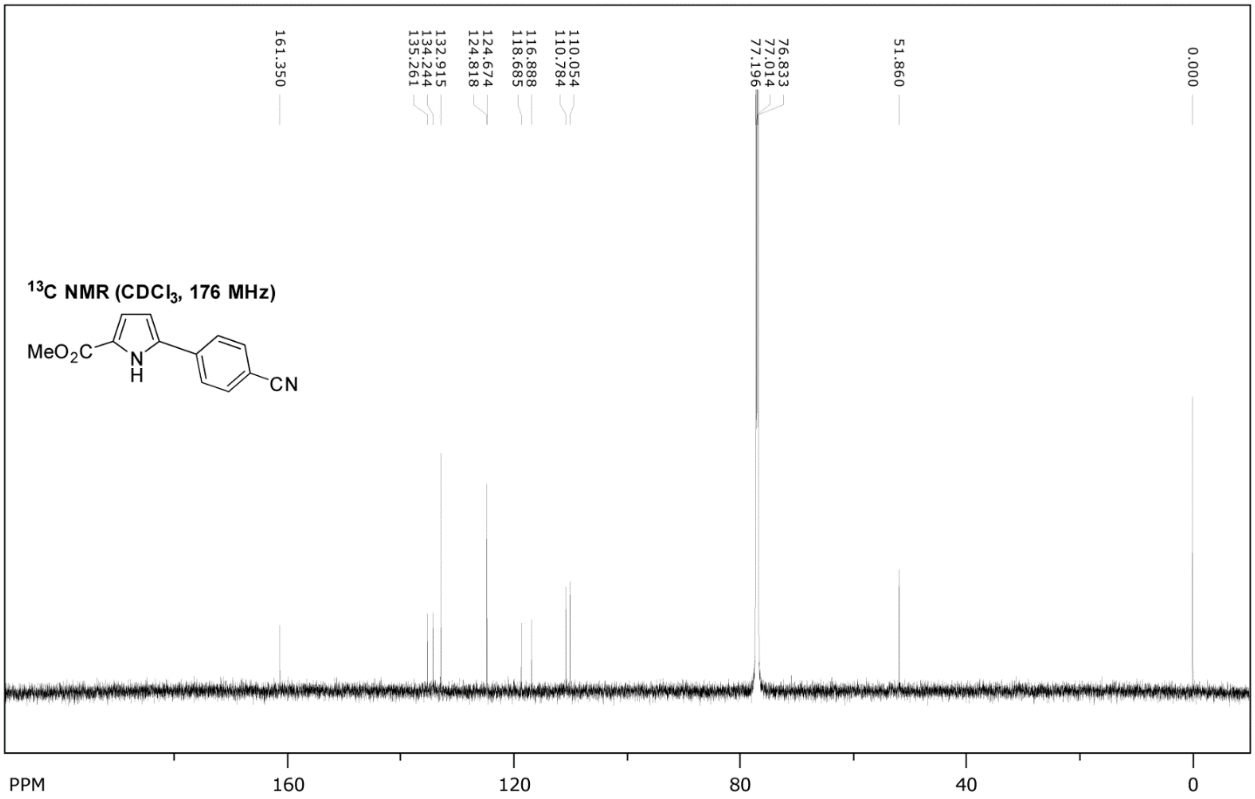

Compound 2g: ^{13}C NMR spectrum of methyl 5-(4-cyanophenyl)-1*H*-pyrrole-2-carboxylate

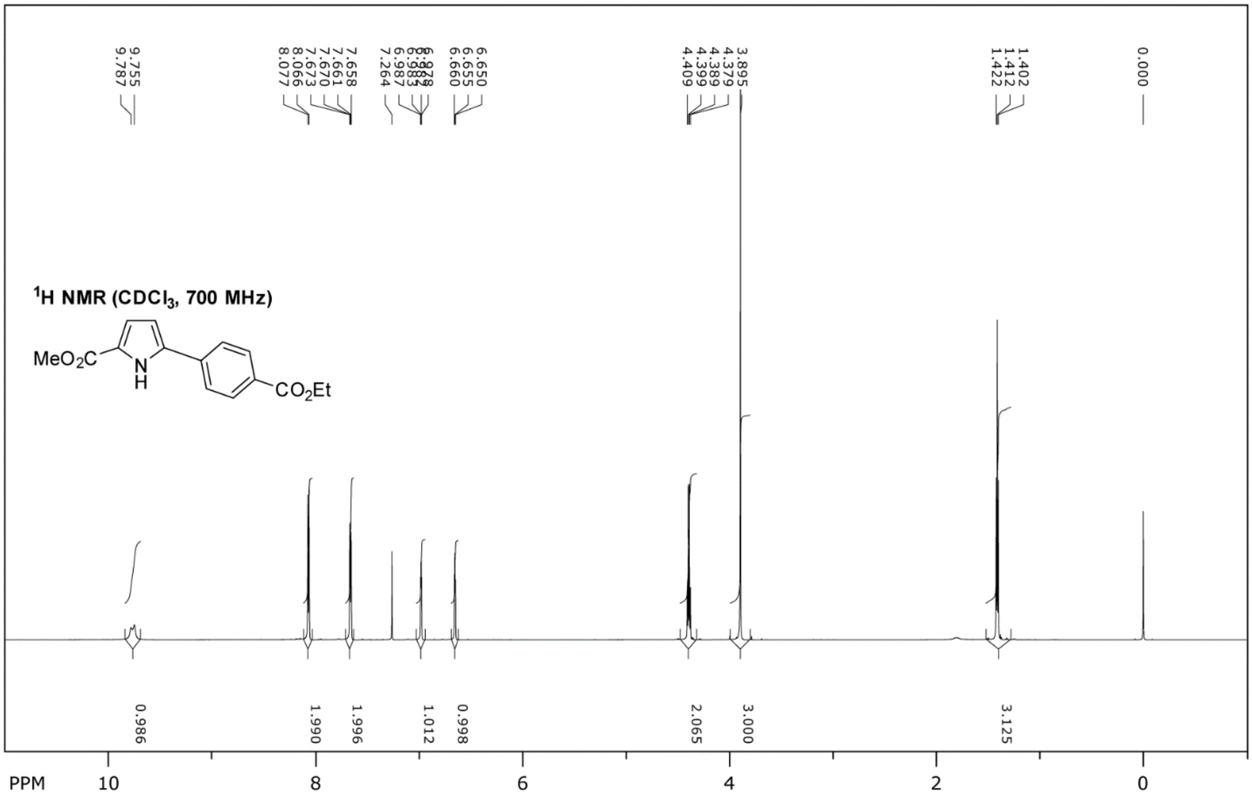

Compound 2h: ^{1}H NMR spectrum of methyl 5-(4-(ethoxycarbonyl)phenyl)-1*H*-pyrrole-2-carboxylate

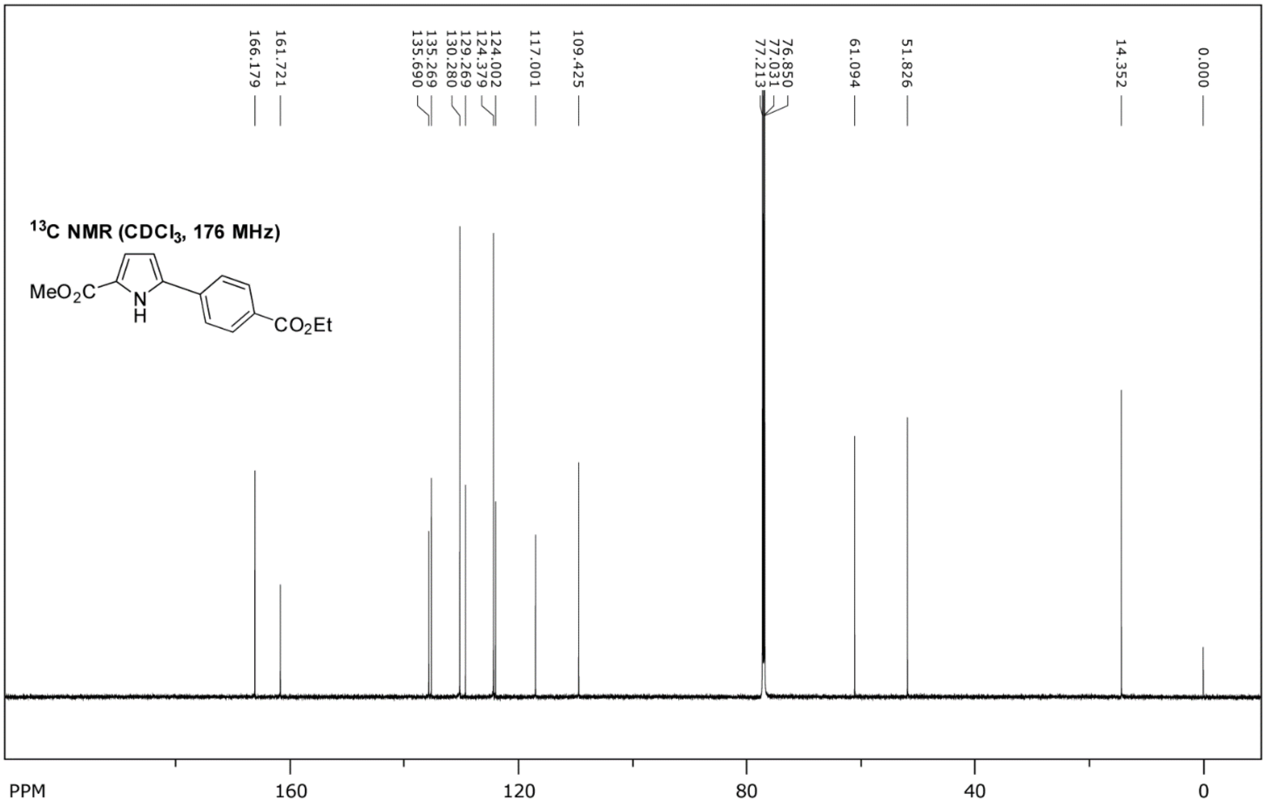

Compound 2h: ^{13}C NMR spectrum of methyl 5-(4-(ethoxycarbonyl)phenyl)-1*H*-pyrrole-2-carboxylate

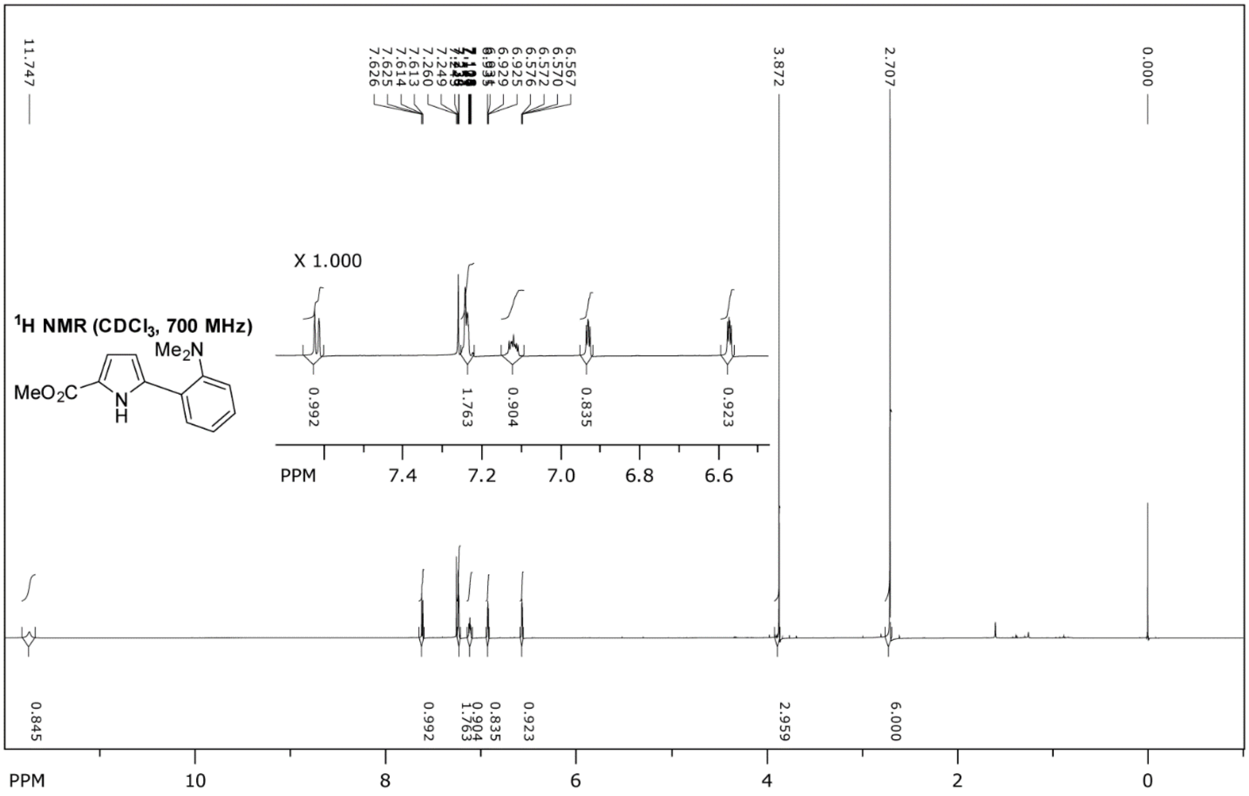

Compound 2i: ^{1}H NMR spectrum of methyl 5-(2-(dimethylamino)phenyl)-1*H*-pyrrole-2-carboxylate

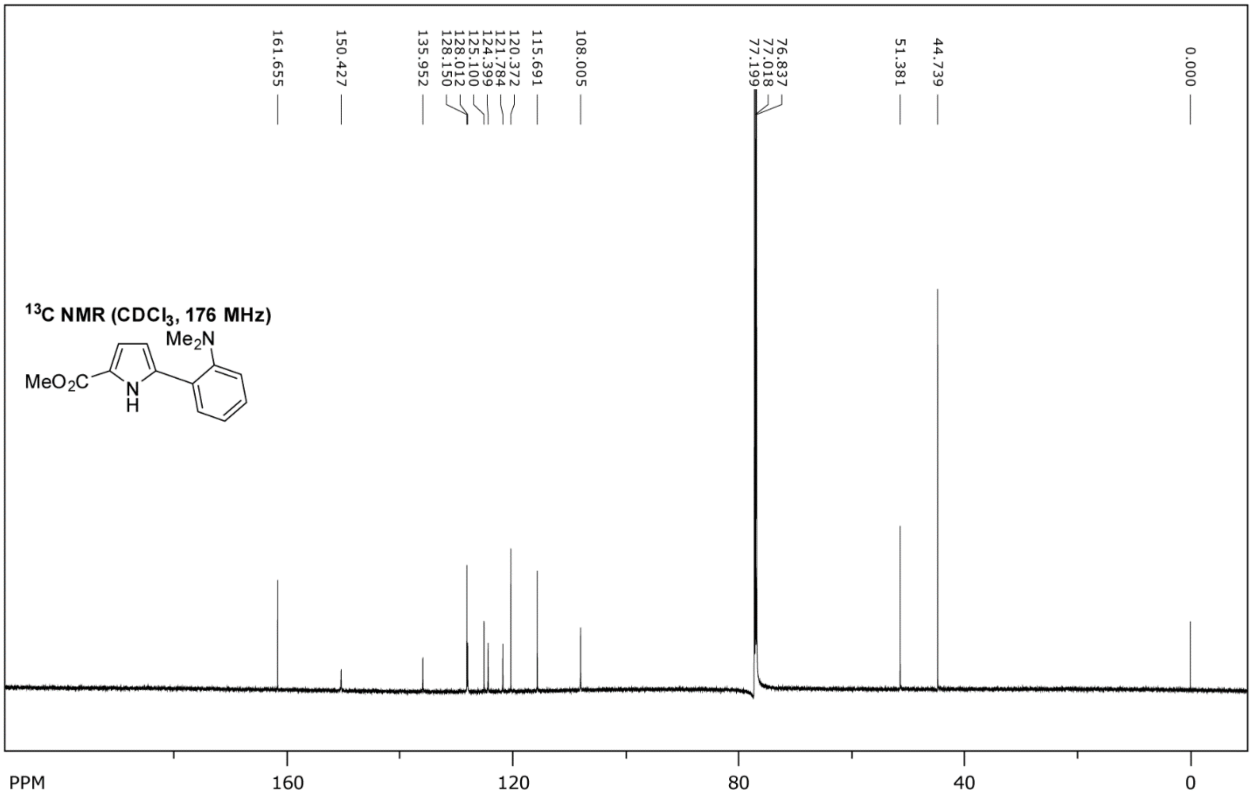

Compound 2i: ^{13}C NMR spectrum of methyl 5-(2-(dimethylamino)phenyl)-1*H*-pyrrole-2-carboxylate

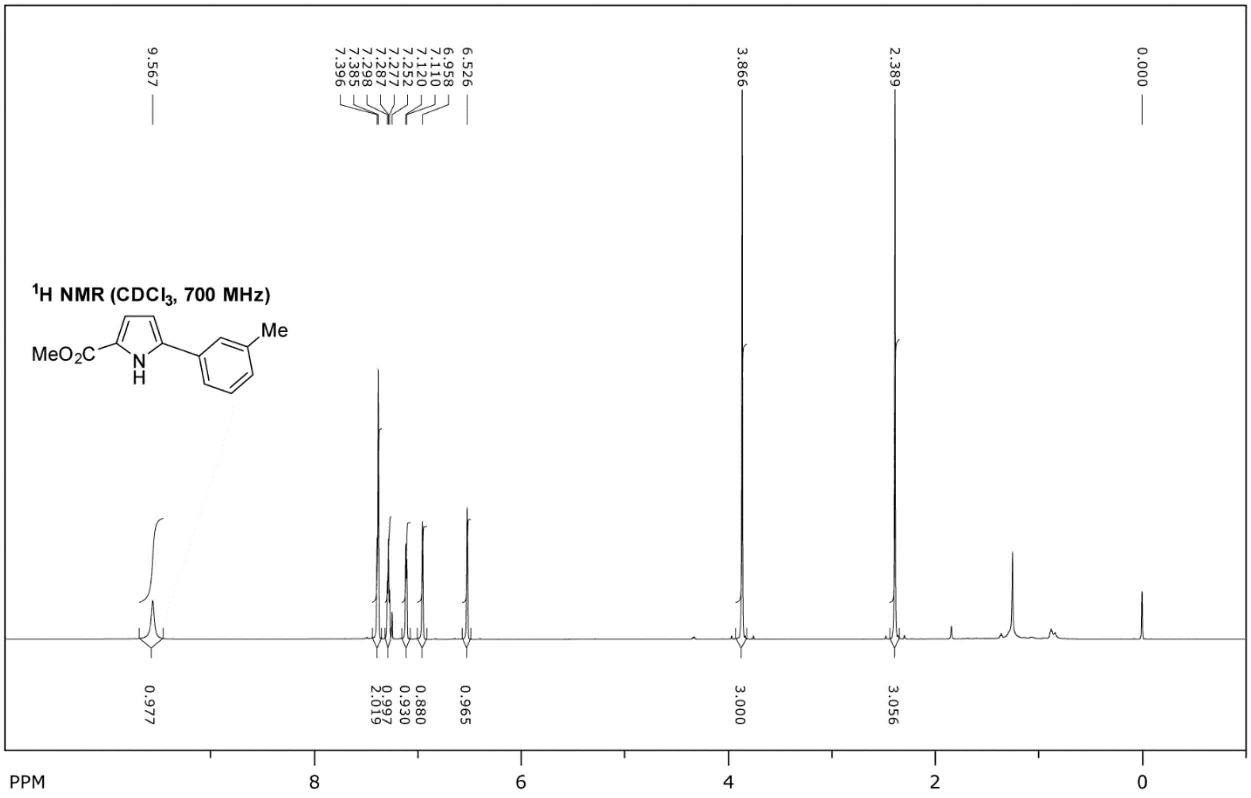

Compound 2j: ^{1}H NMR spectrum of methyl 5-(m-tolyl)-1*H*-pyrrole-2-carboxylate

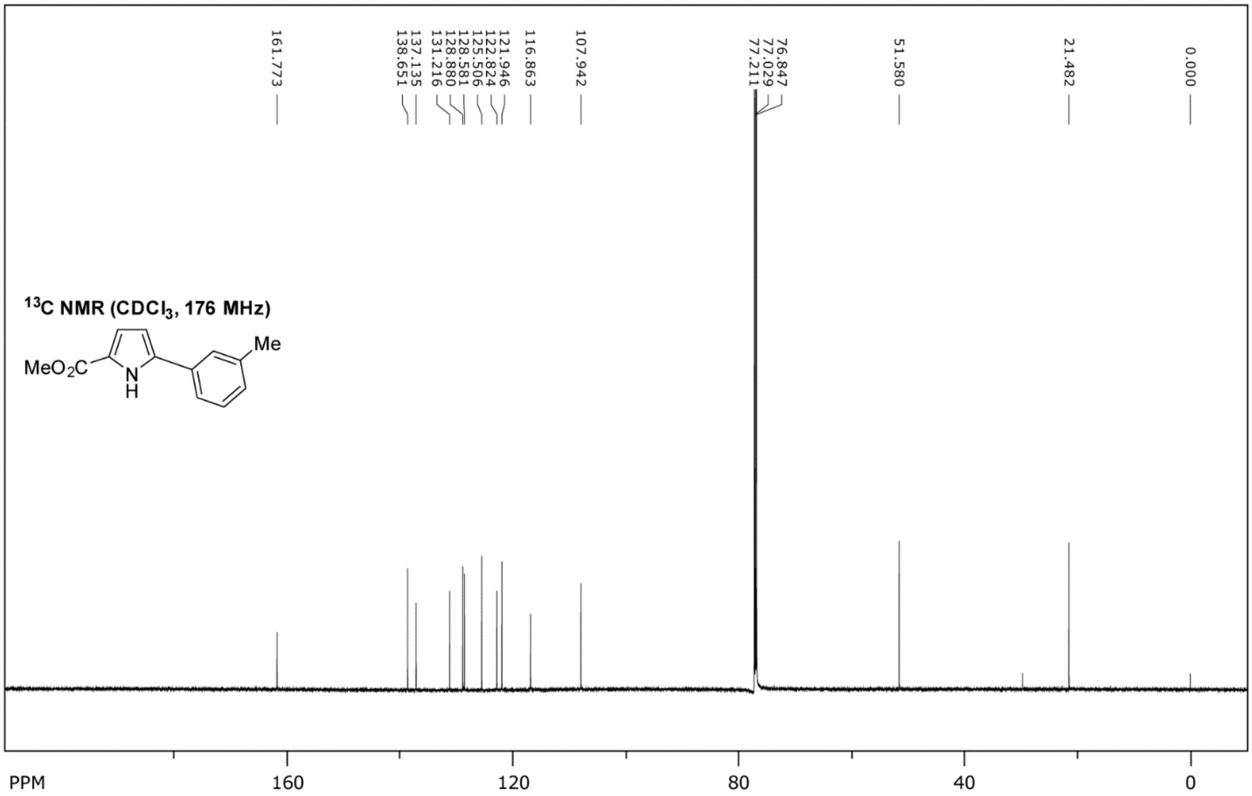

Compound 2j: ^{13}C NMR spectrum of methyl 5-(m-tolyl)-1*H*-pyrrole-2-carboxylate

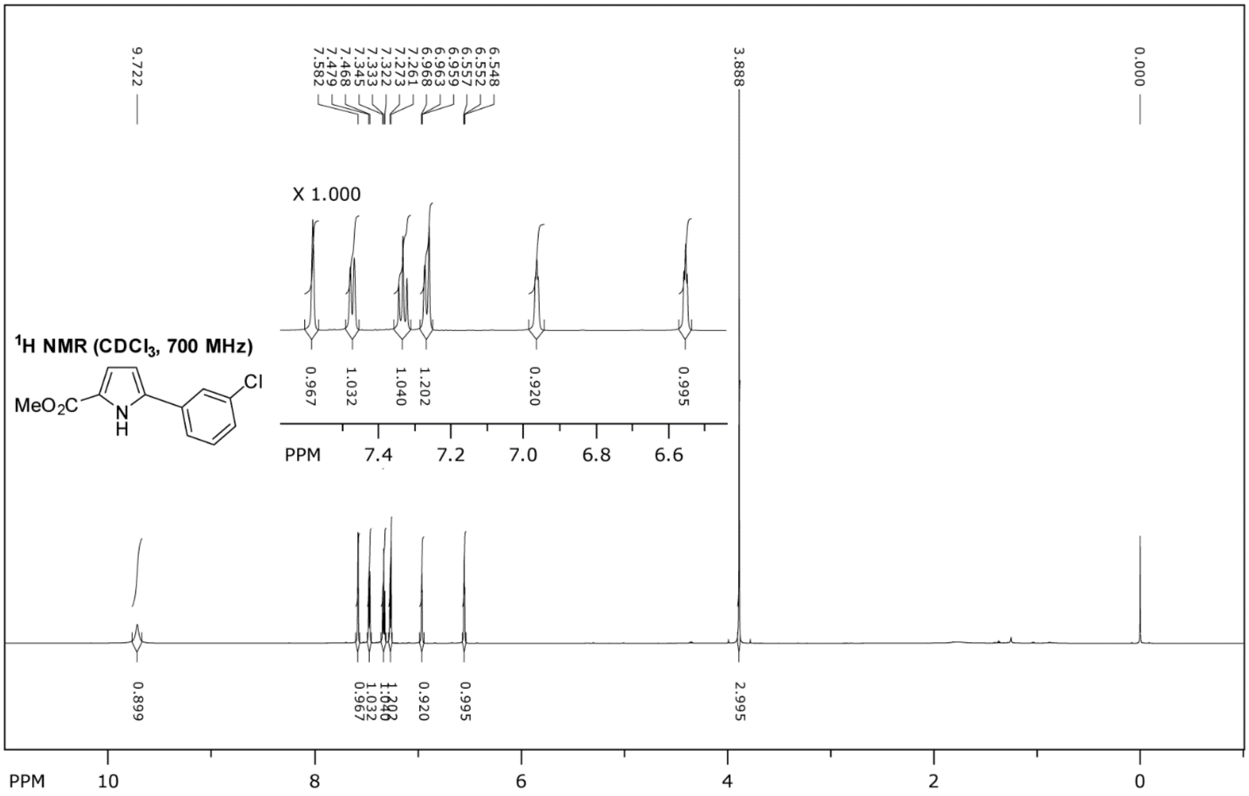

Compound 2k: ^{1}H NMR spectrum of methyl 5-(3-chlorophenyl)-1*H*-pyrrole-2-carboxylate

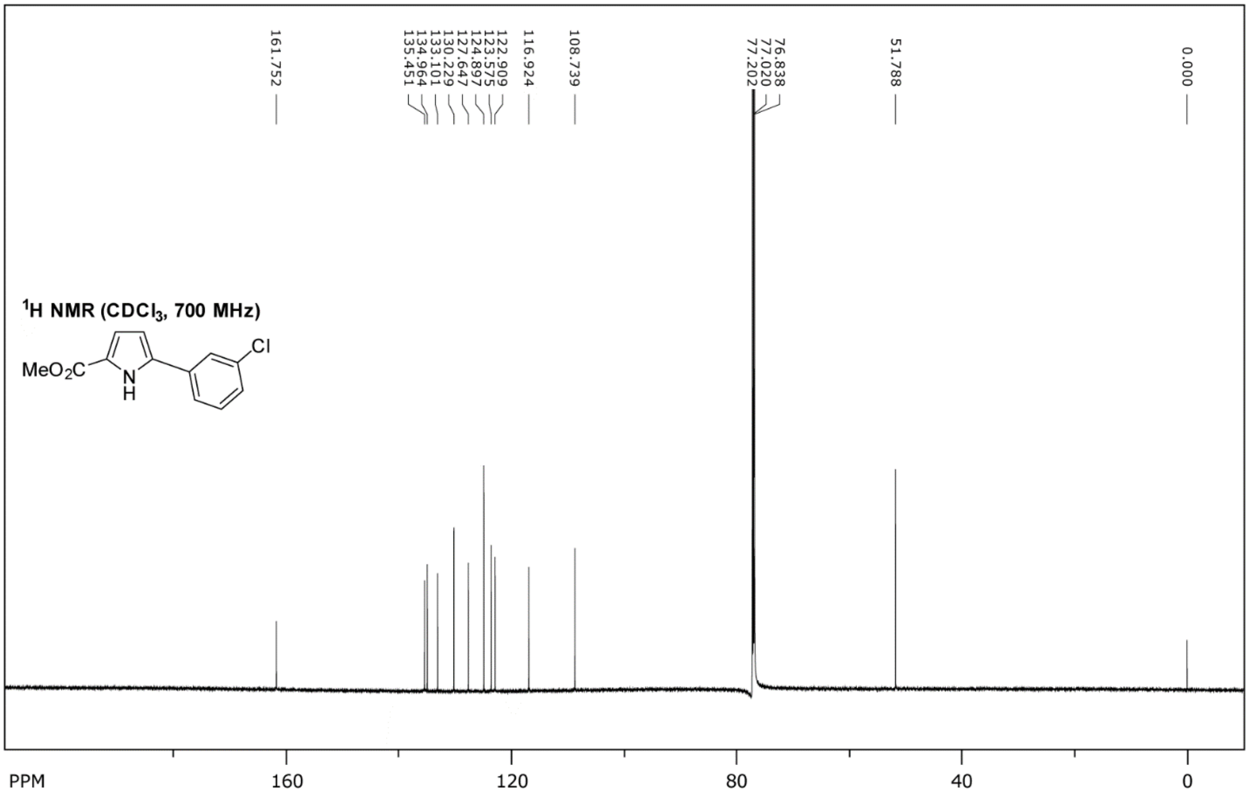

Compound 2k: ^{13}C NMR spectrum of methyl 5-(3-chlorophenyl)-1*H*-pyrrole-2-carboxylate

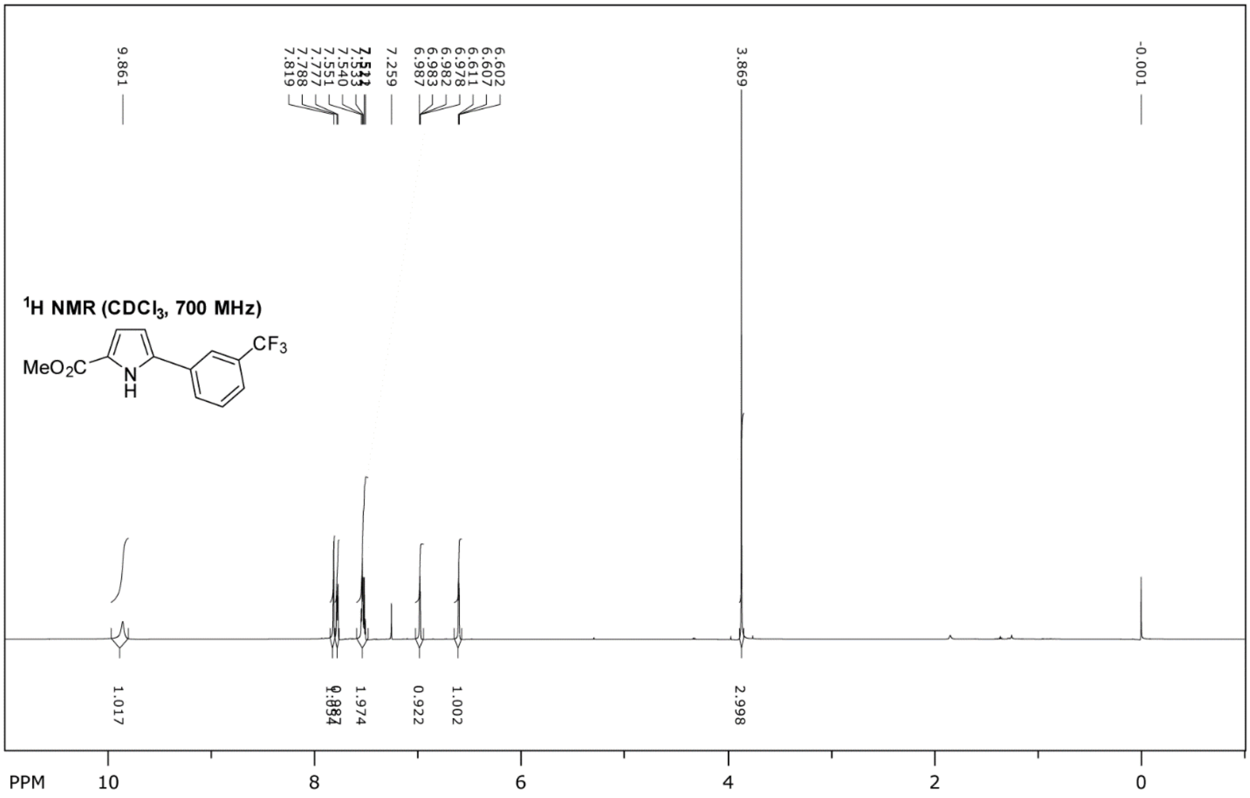

Compound 2l: ^{1}H NMR spectrum of methyl 5-(3-(trifluoromethyl)phenyl)-1*H*-pyrrole-2-carboxylate

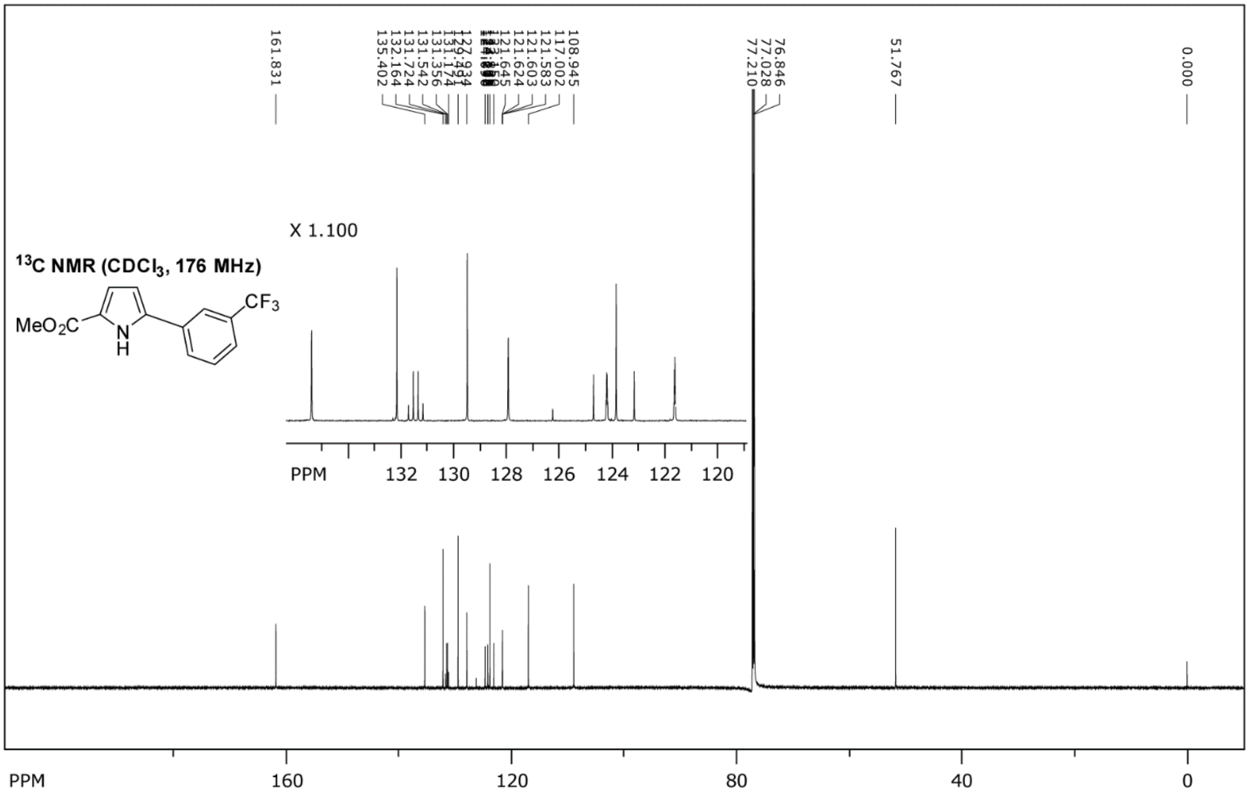

Compound 2l: ^{13}C NMR spectrum of methyl 5-(3-(trifluoromethyl)phenyl)-1*H*-pyrrole-2-carboxylate

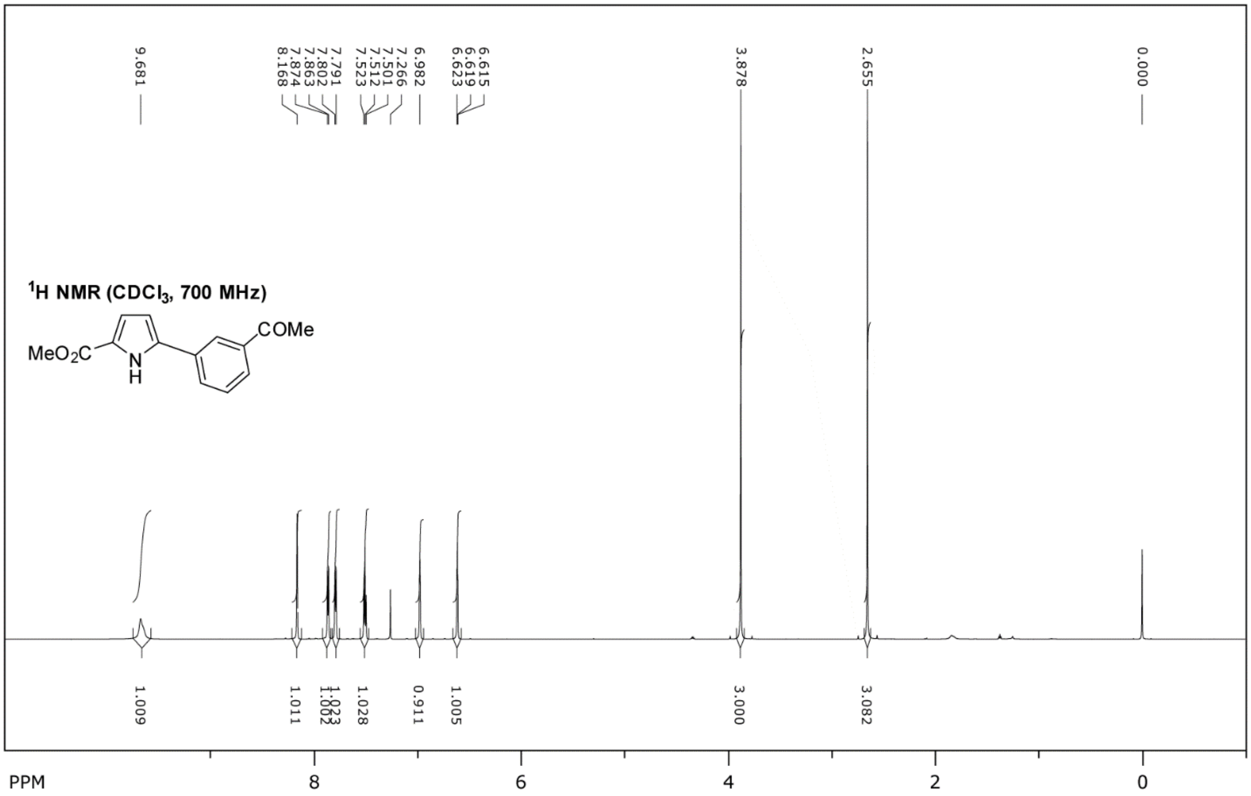

Compound 2m: ^{1}H NMR spectrum of methyl 5-(3-acetylphenyl)-1*H*-pyrrole-2-carboxylate

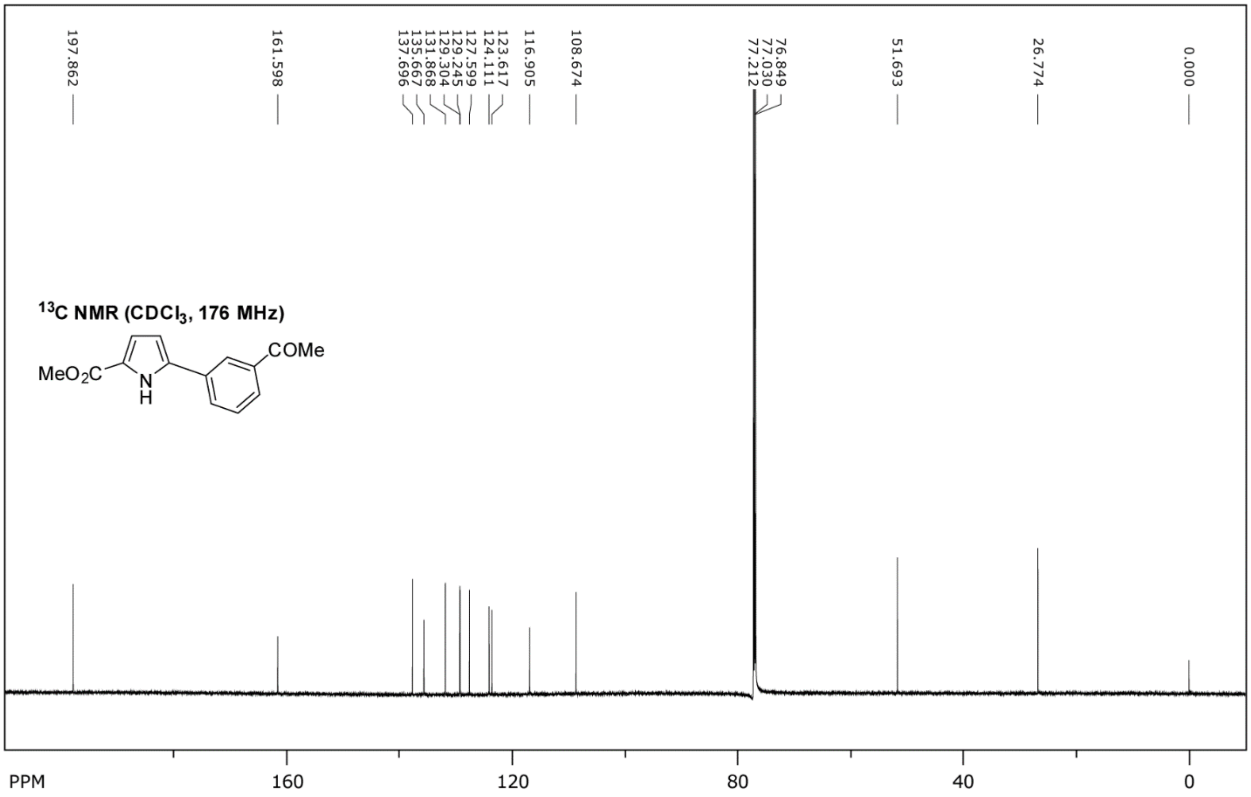

Compound 2m: ^{13}C NMR spectrum of methyl 5-(3-acetylphenyl)-1*H*-pyrrole-2-carboxylate

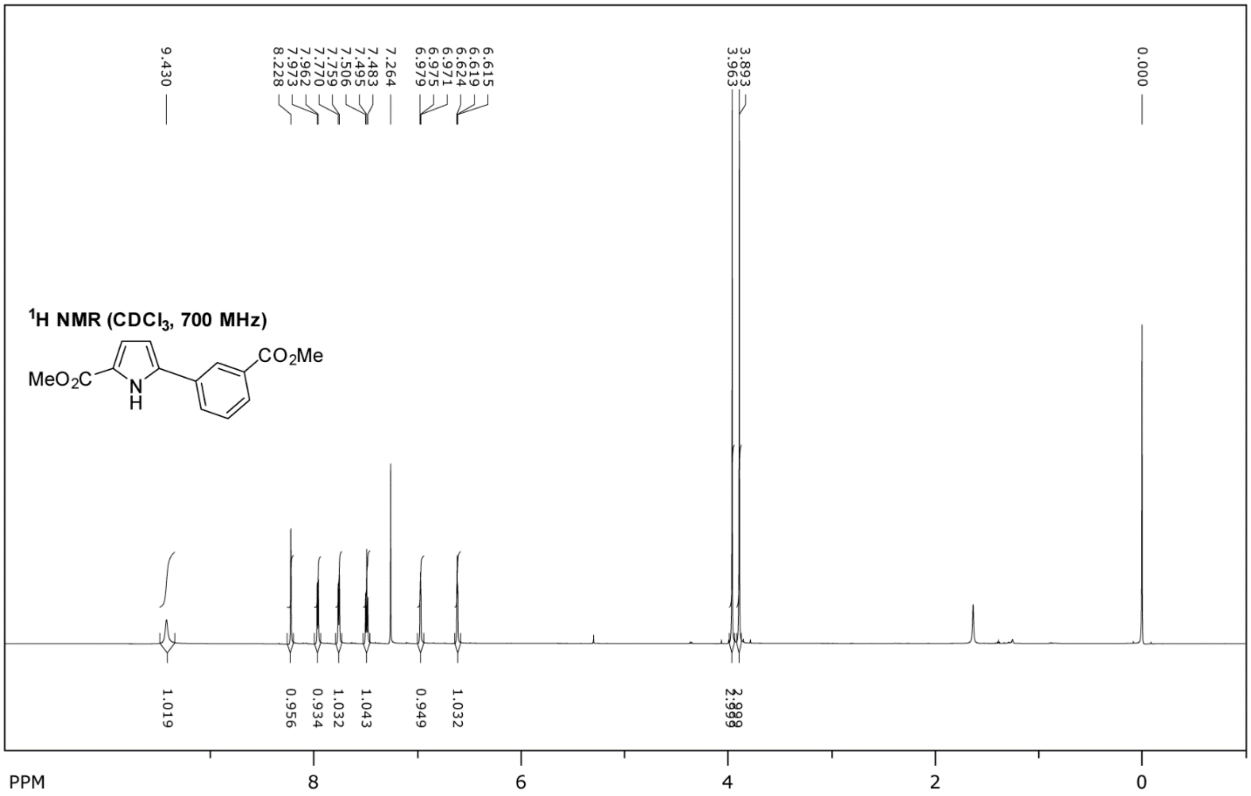

Compound 2n: ¹H NMR spectrum of methyl 5-(3-(methoxycarbonyl)phenyl)-1*H*-pyrrole-2-carboxylate

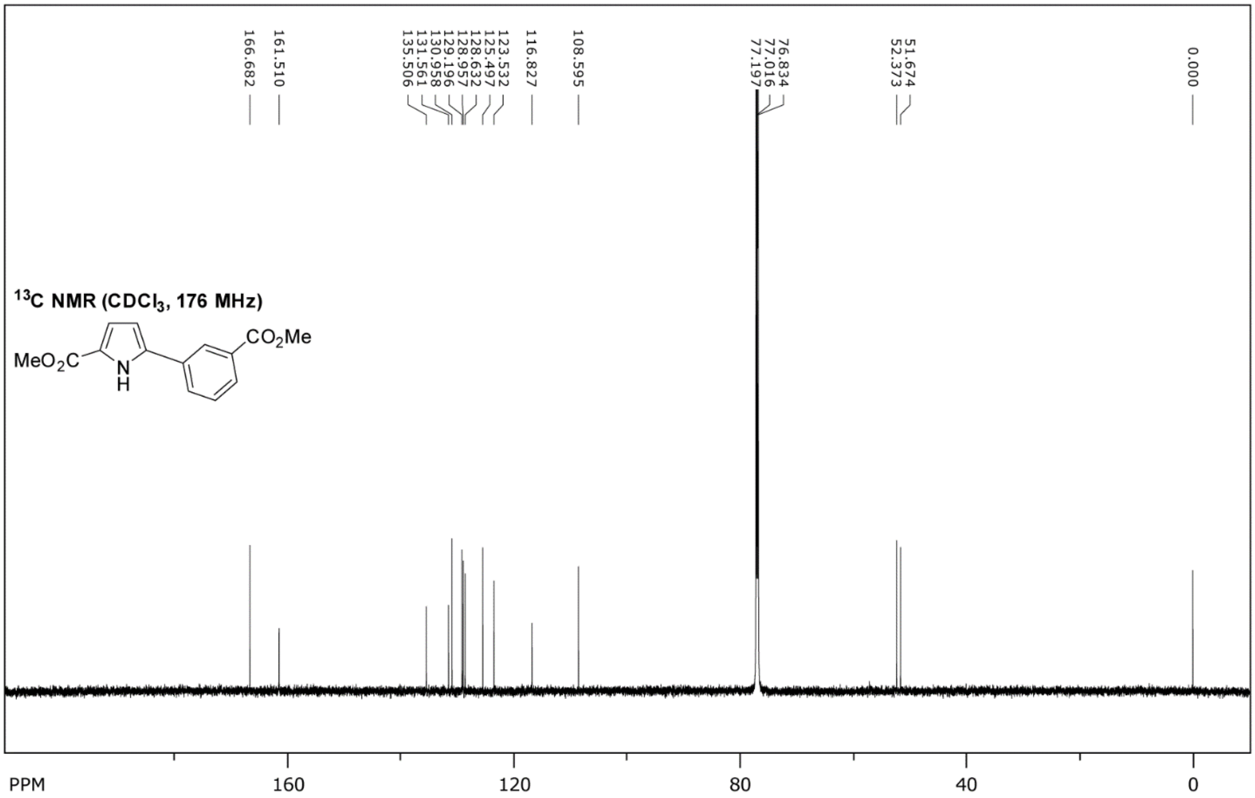

Compound 2n: ^{13}C NMR spectrum of methyl 5-(3-(methoxycarbonyl)phenyl)-1*H*-pyrrole-2-carboxylate

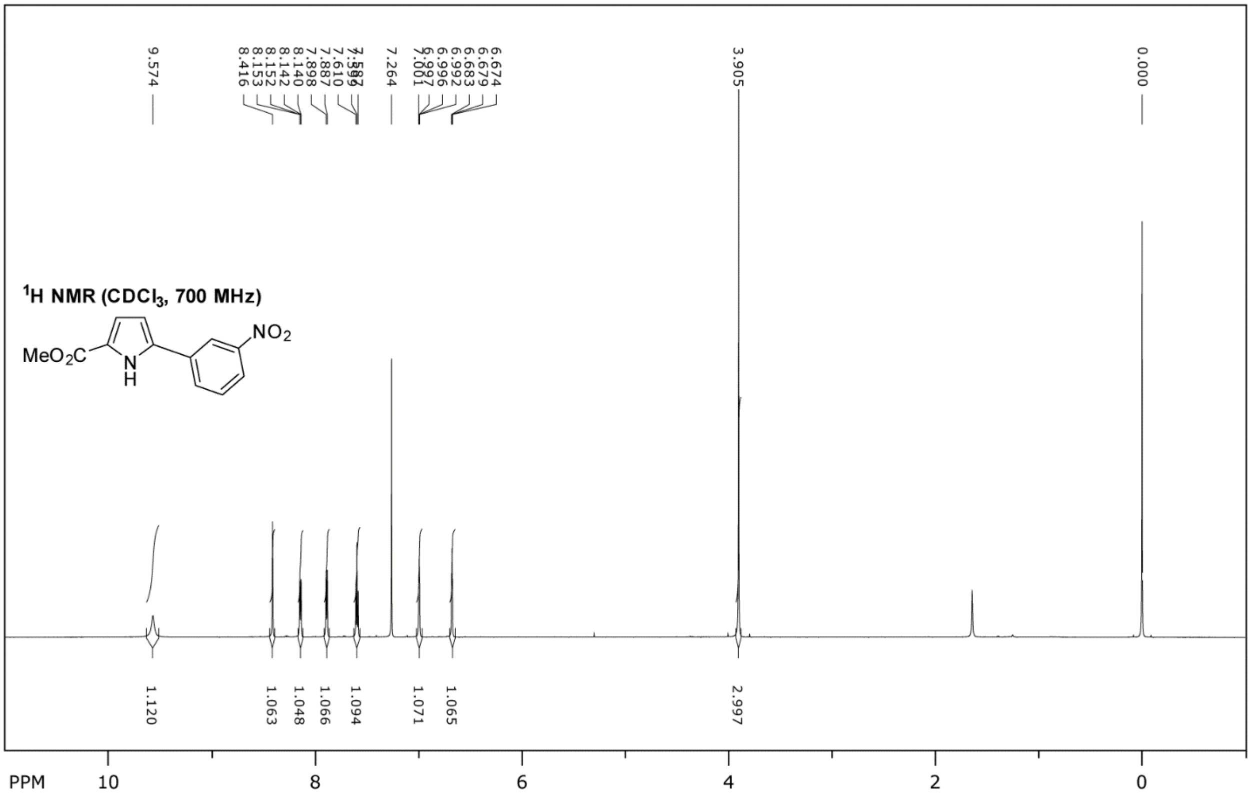

Compound 2o: ^{1}H NMR spectrum of methyl 5-(3-nitrophenyl)-1*H*-pyrrole-2-carboxylate

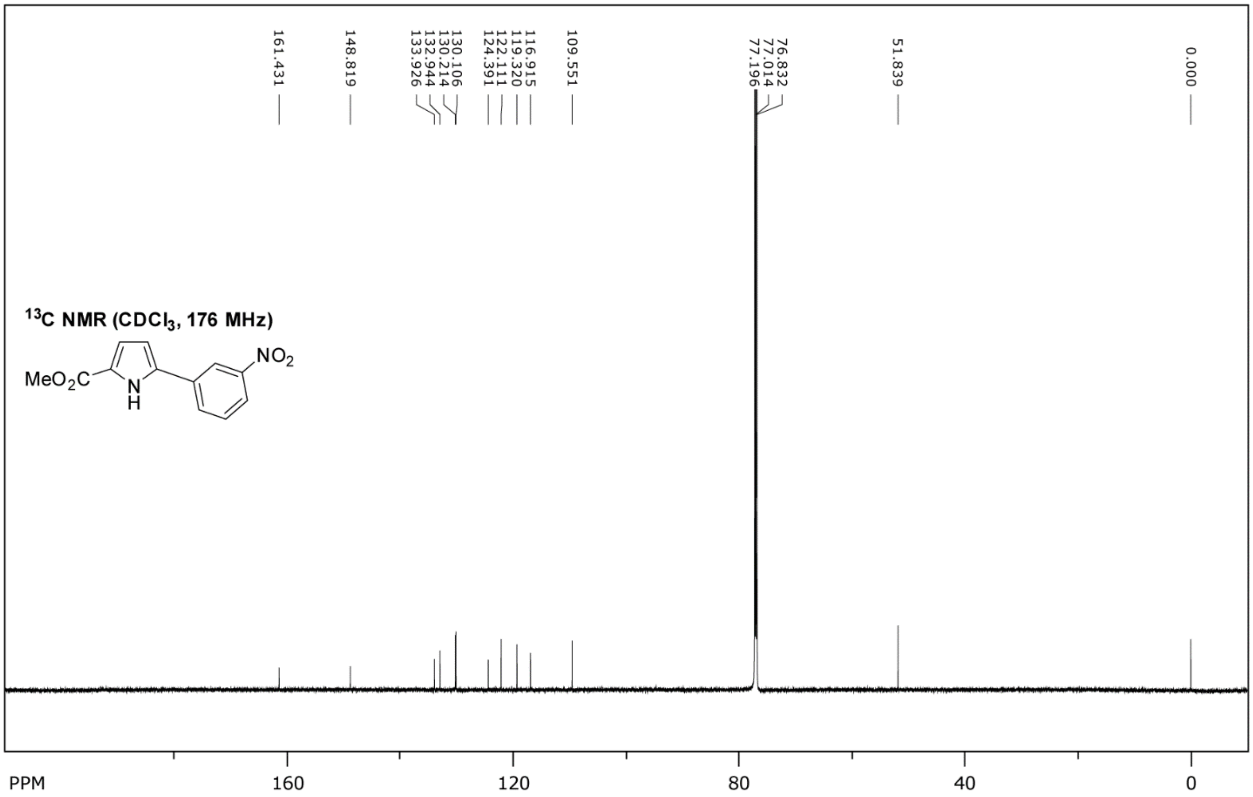

Compound 2o: ^{13}C NMR spectrum of methyl 5-(3-nitrophenyl)-1*H*-pyrrole-2-carboxylate

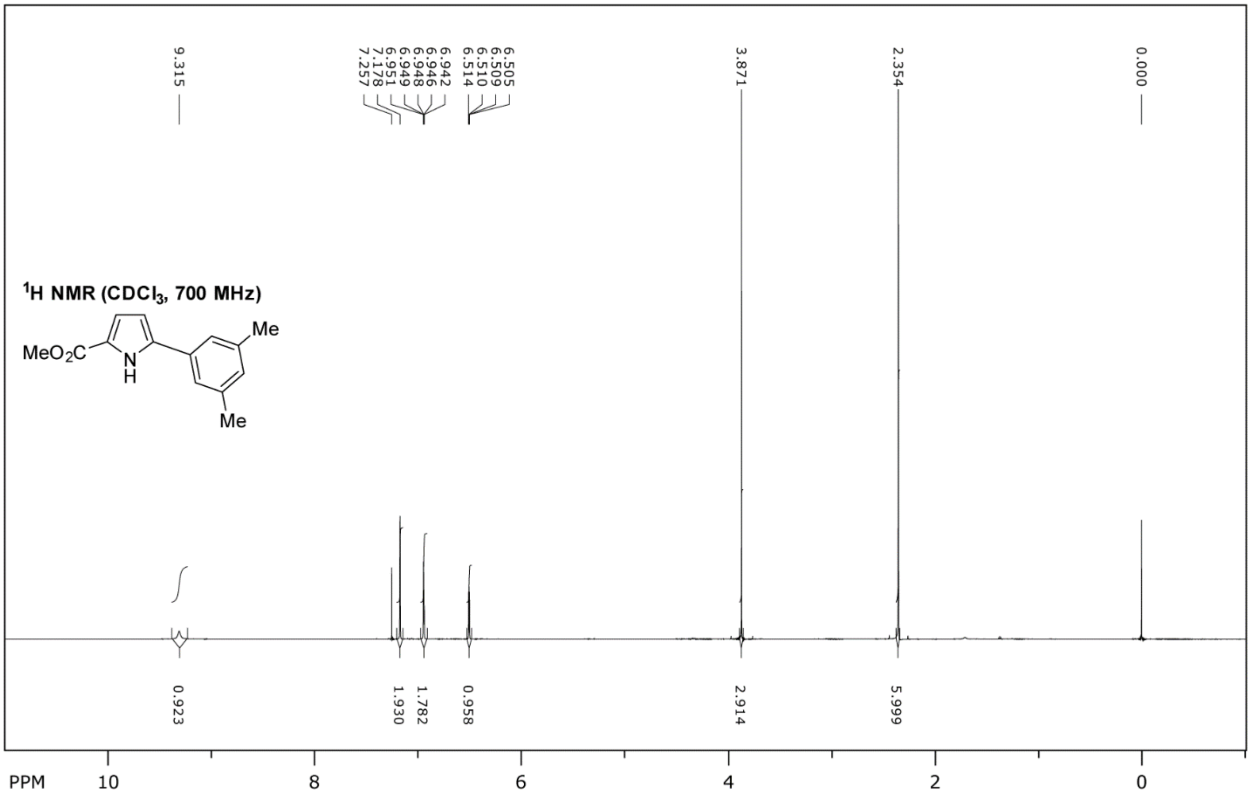

Compound 2p: ^{1}H NMR spectrum of methyl 5-(3,5-dimethylphenyl)-1*H*-pyrrole-2-carboxylate

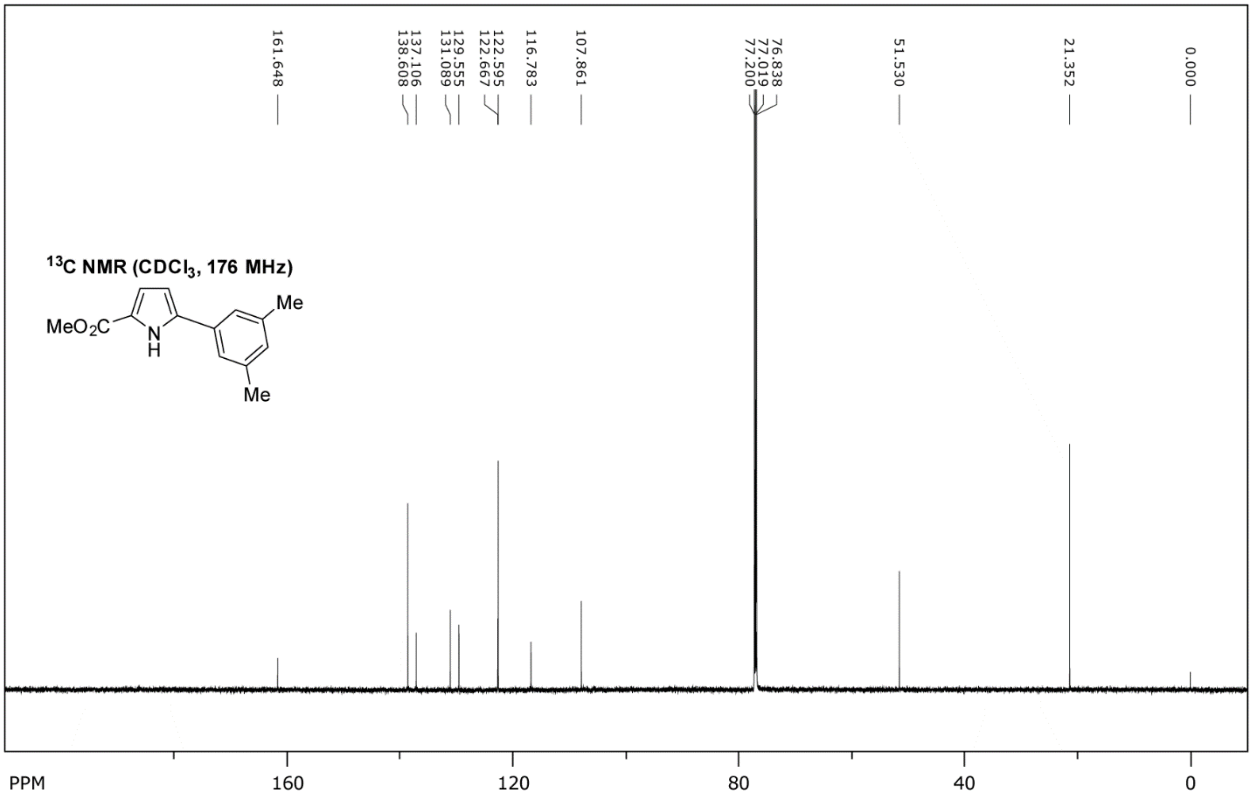

Compound 2p: ^{13}C NMR spectrum of methyl 5-(3,5-dimethylphenyl)-1*H*-pyrrole-2-carboxylate

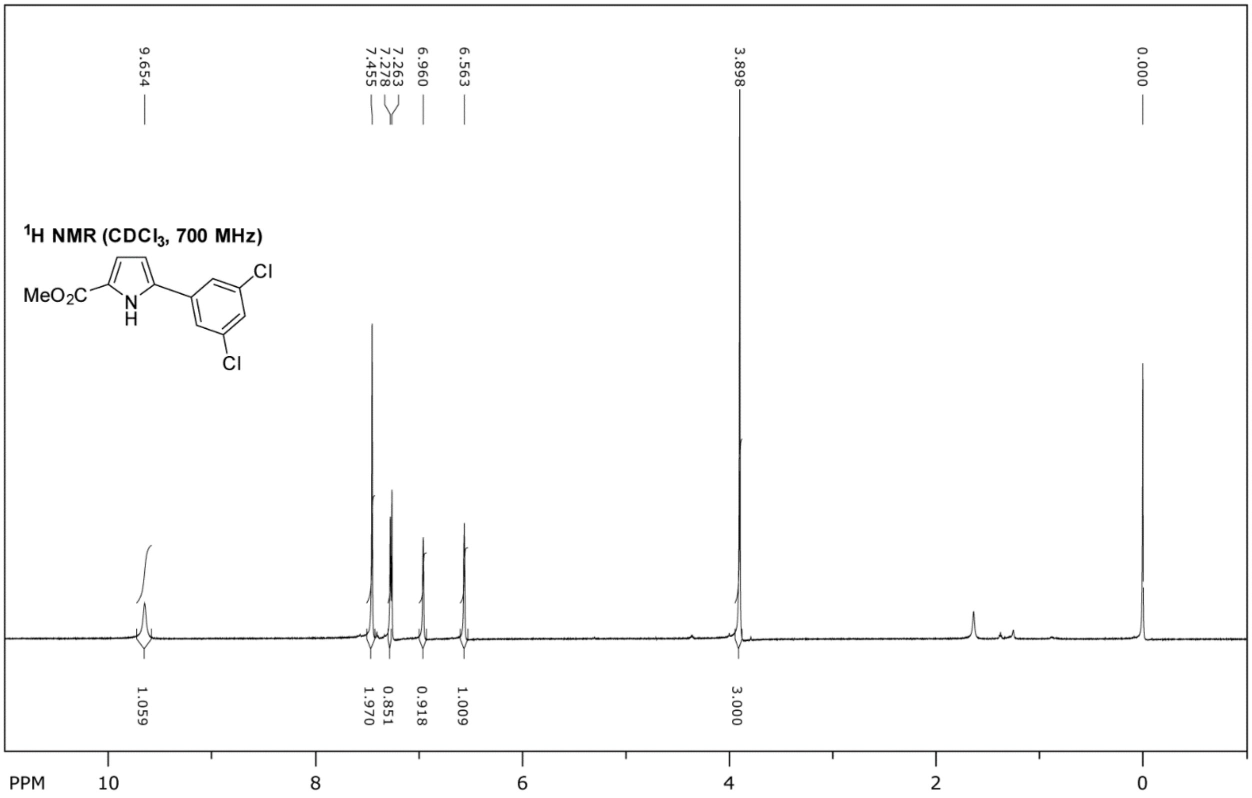

Compound 2q: ^{1}H NMR spectrum of methyl 5-(3,5-dichlorophenyl)-1*H*-pyrrole-2-carboxylate

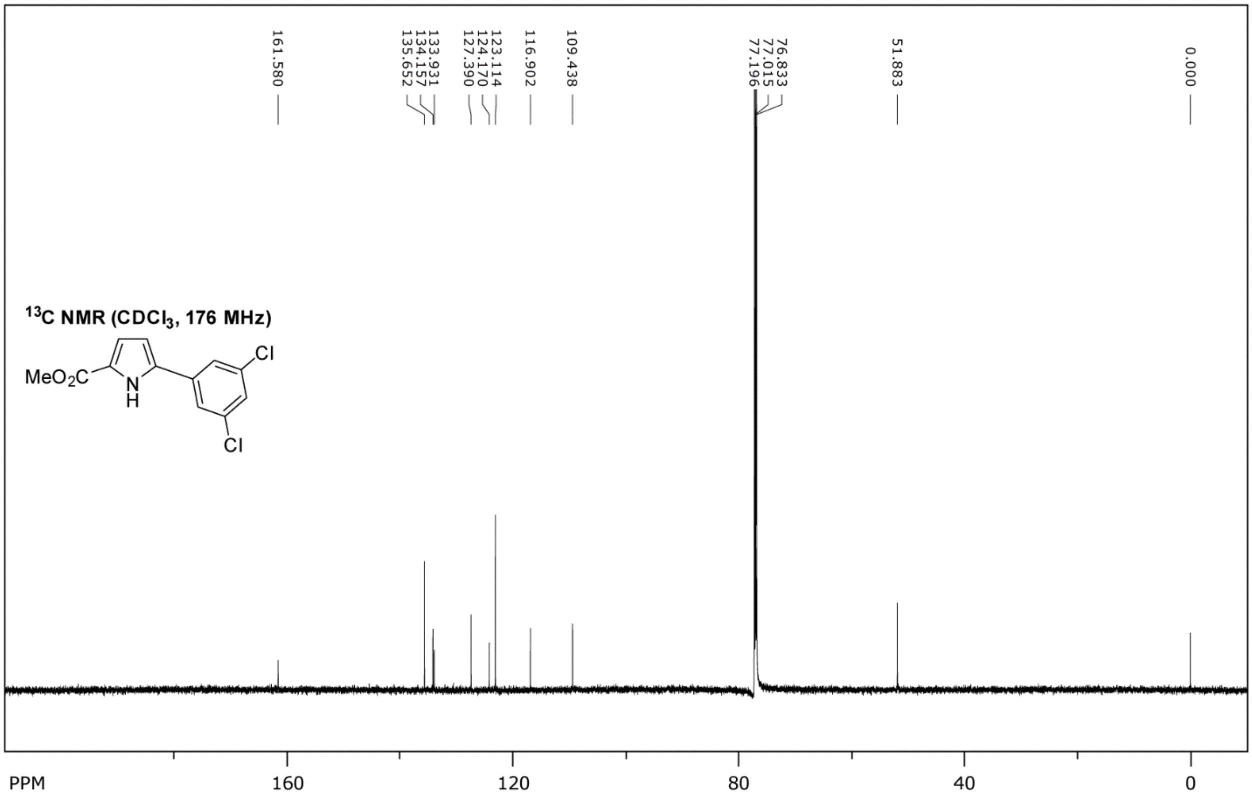

Compound 2q: ^{13}C NMR spectrum of methyl 5-(3,5-dichlorophenyl)-1*H*-pyrrole-2-carboxylate

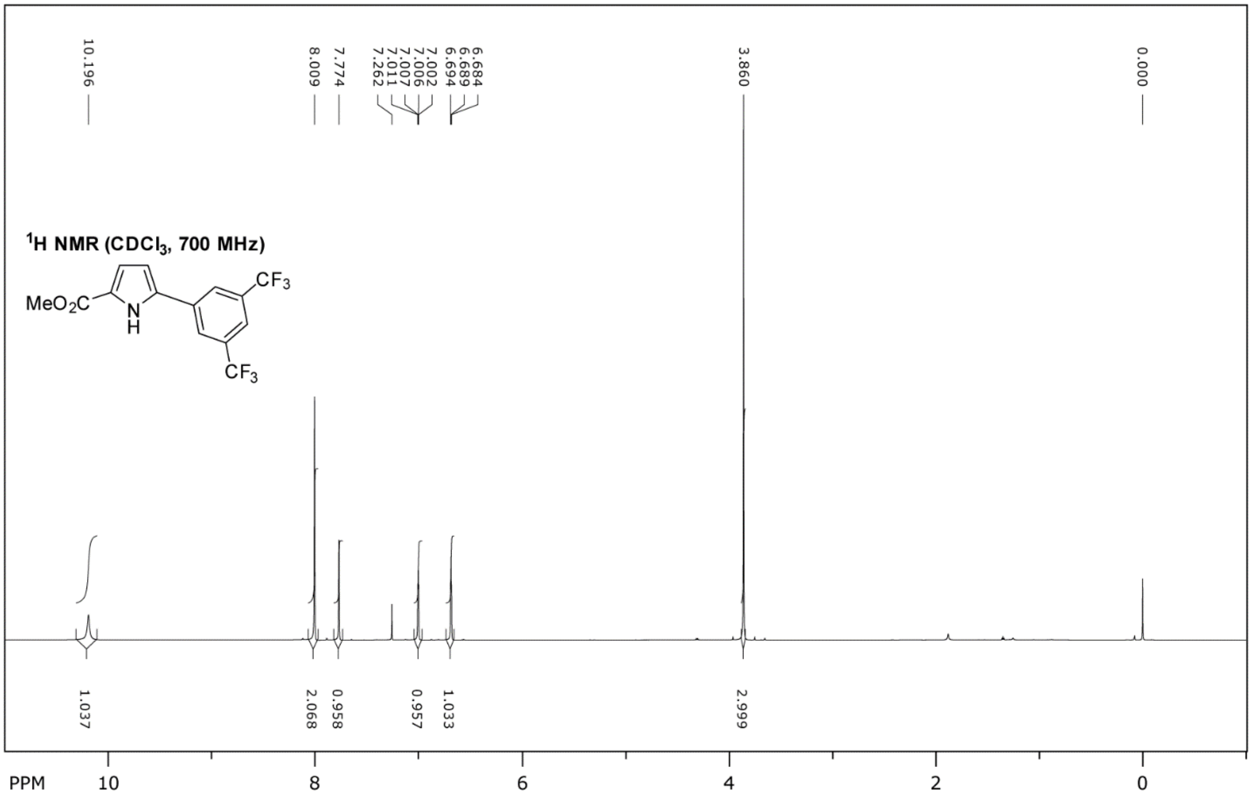

Compound 2r: ^{1}H NMR spectrum of methyl 5-(3,5-bis(trifluoromethyl)phenyl)-1*H*-pyrrole-2-carboxylate

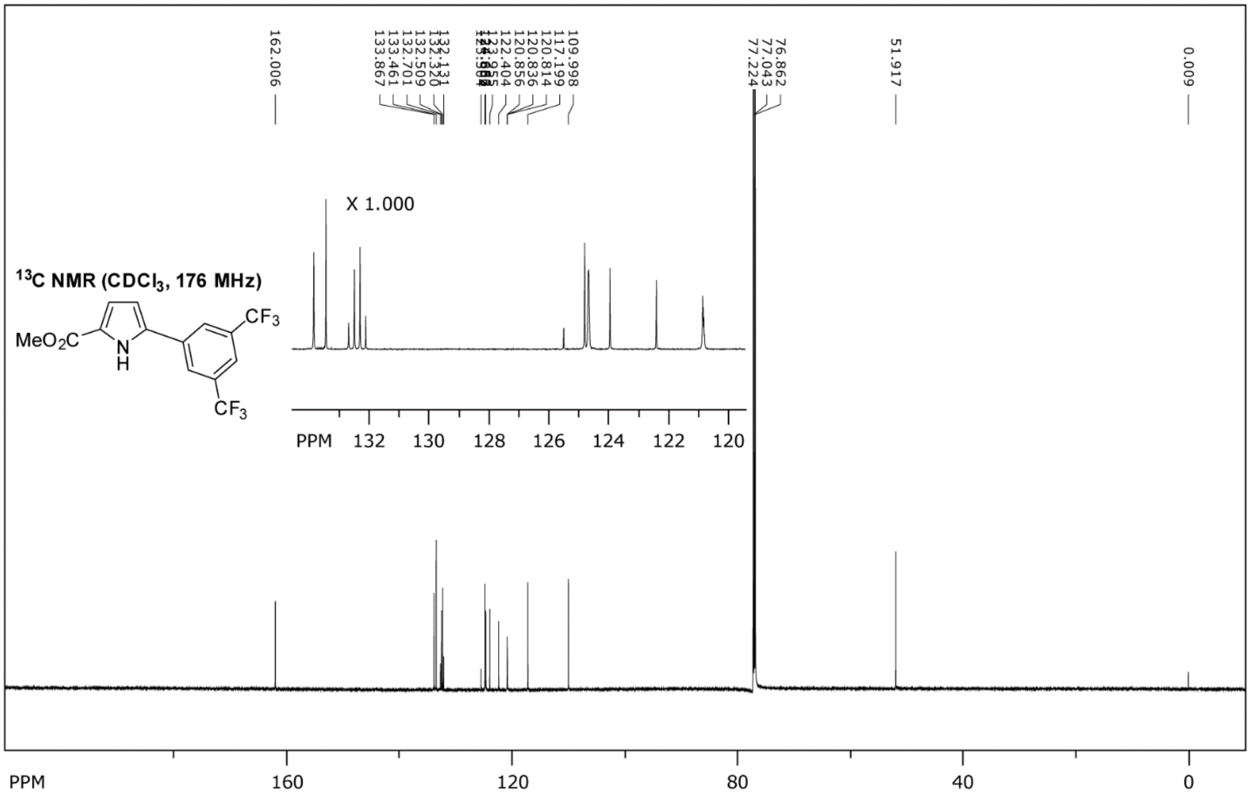

Compound 2r: ^{13}C NMR spectrum of methyl 5-(3,5-bis(trifluoromethyl)phenyl)-1*H*-pyrrole-2-carboxylate

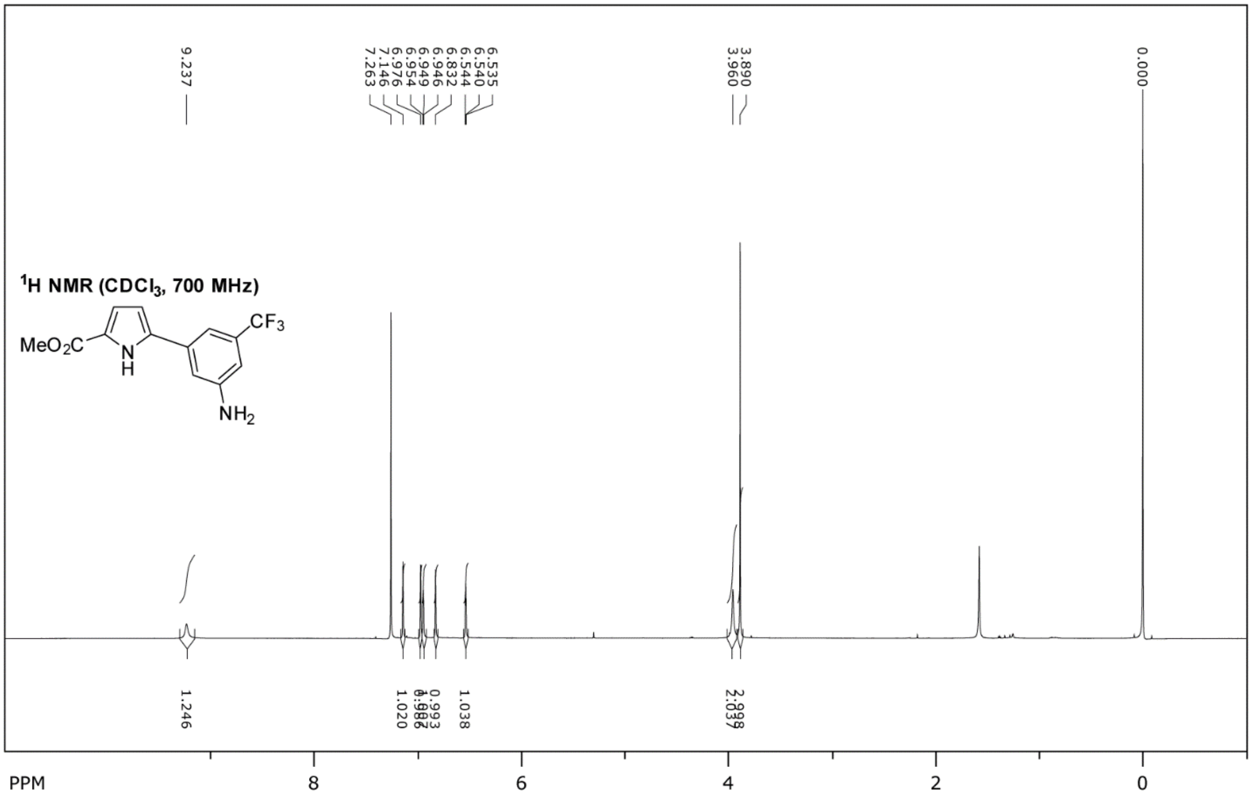

Compound 2s: ^{1}H NMR spectrum of methyl 5-(3-amino-5-(trifluoromethyl)phenyl)-1*H*-pyrrole-2-carboxylate

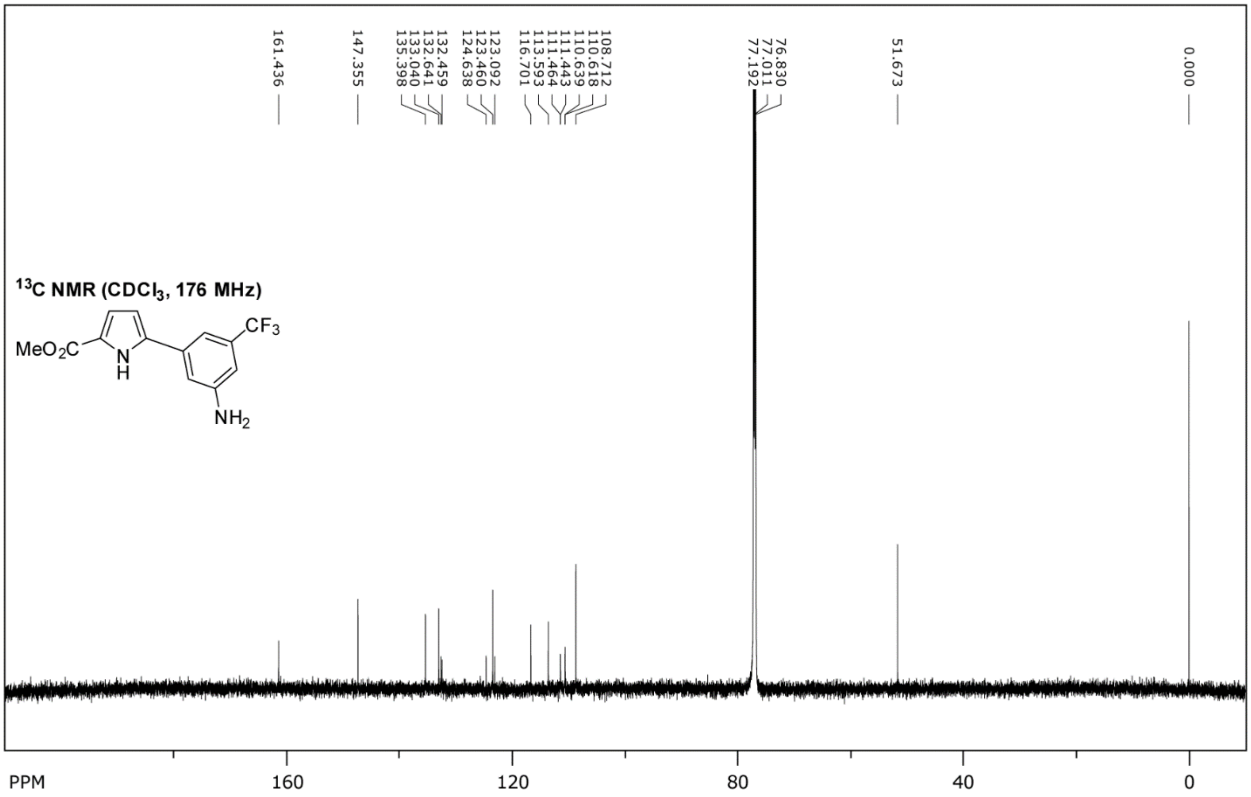

Compound 2s: ^{13}C NMR spectrum of methyl 5-(3-amino-5-(trifluoromethyl)phenyl)-1*H*-pyrrole-2-carboxylate

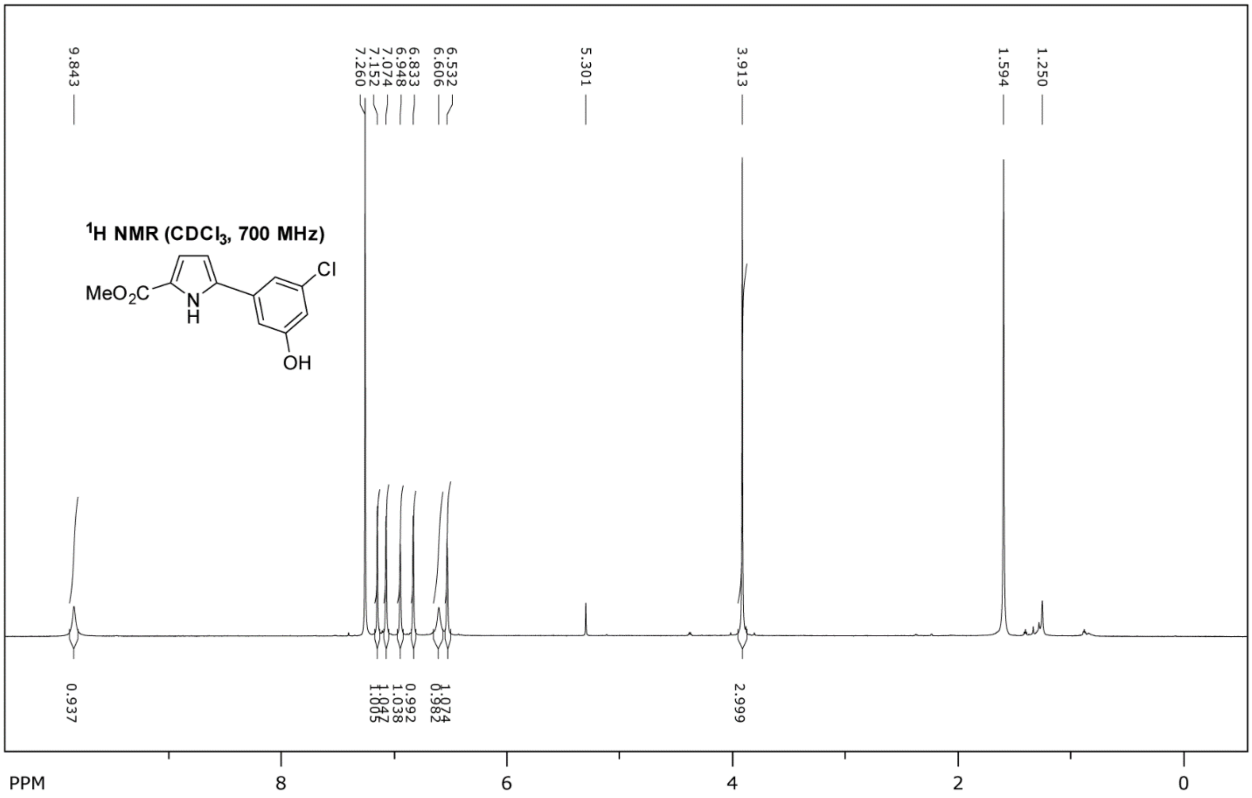

Compound 2t: ^{1}H NMR spectrum of methyl 5-(3-chloro-5-hydroxyphenyl)-1*H*-pyrrole-2-carboxylate

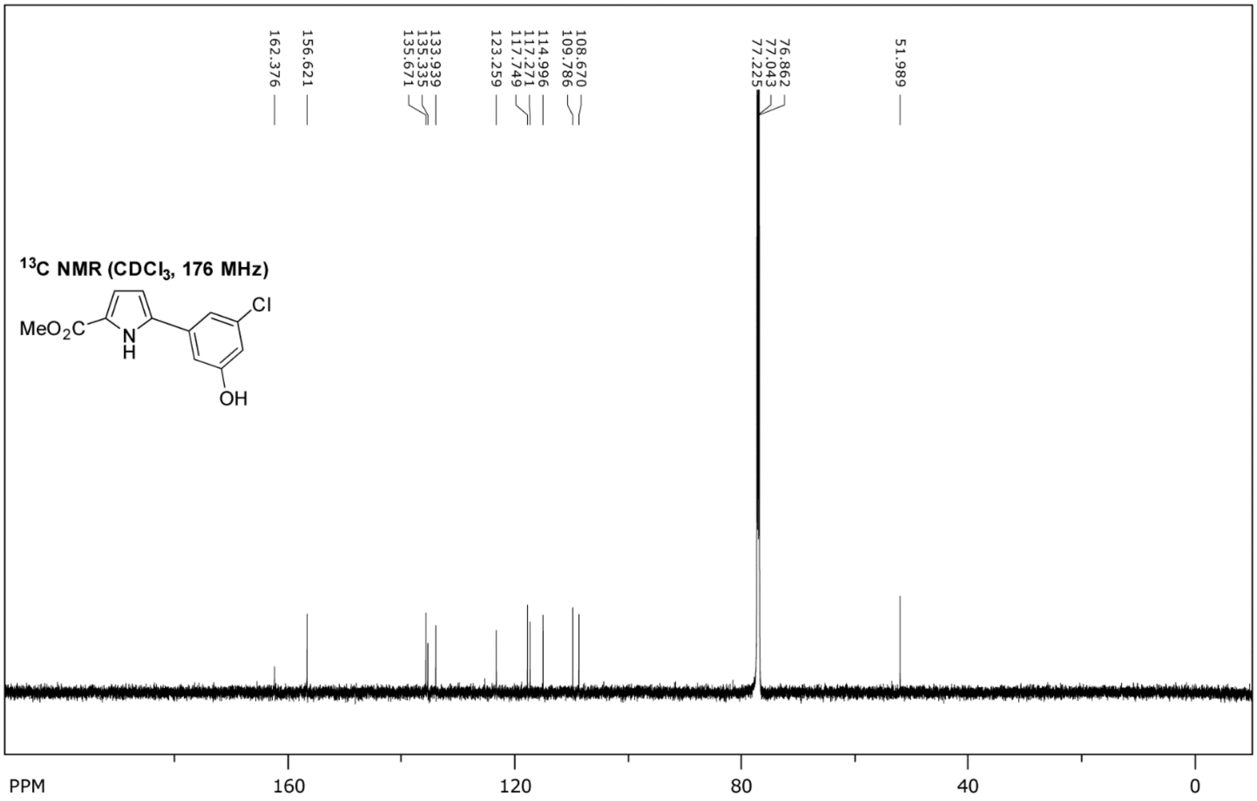

Compound 2t: ^{13}C NMR spectrum of methyl 5-(3-chloro-5-hydroxyphenyl)-1*H*-pyrrole-2-carboxylate

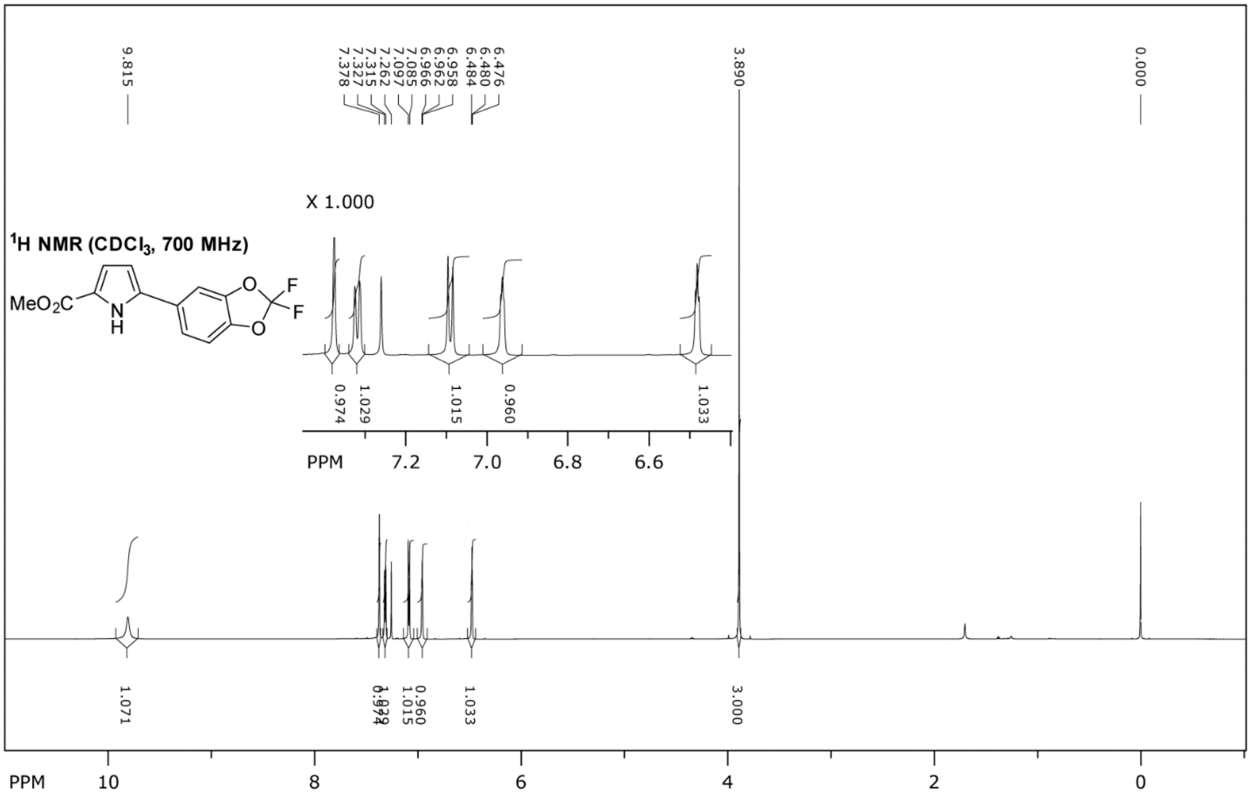

Compound 2u: ^{1}H NMR spectrum of methyl 5-(2,2-difluorobenzo[d][1,3]dioxol-5-yl)-1*H*-pyrrole-2-carboxylate

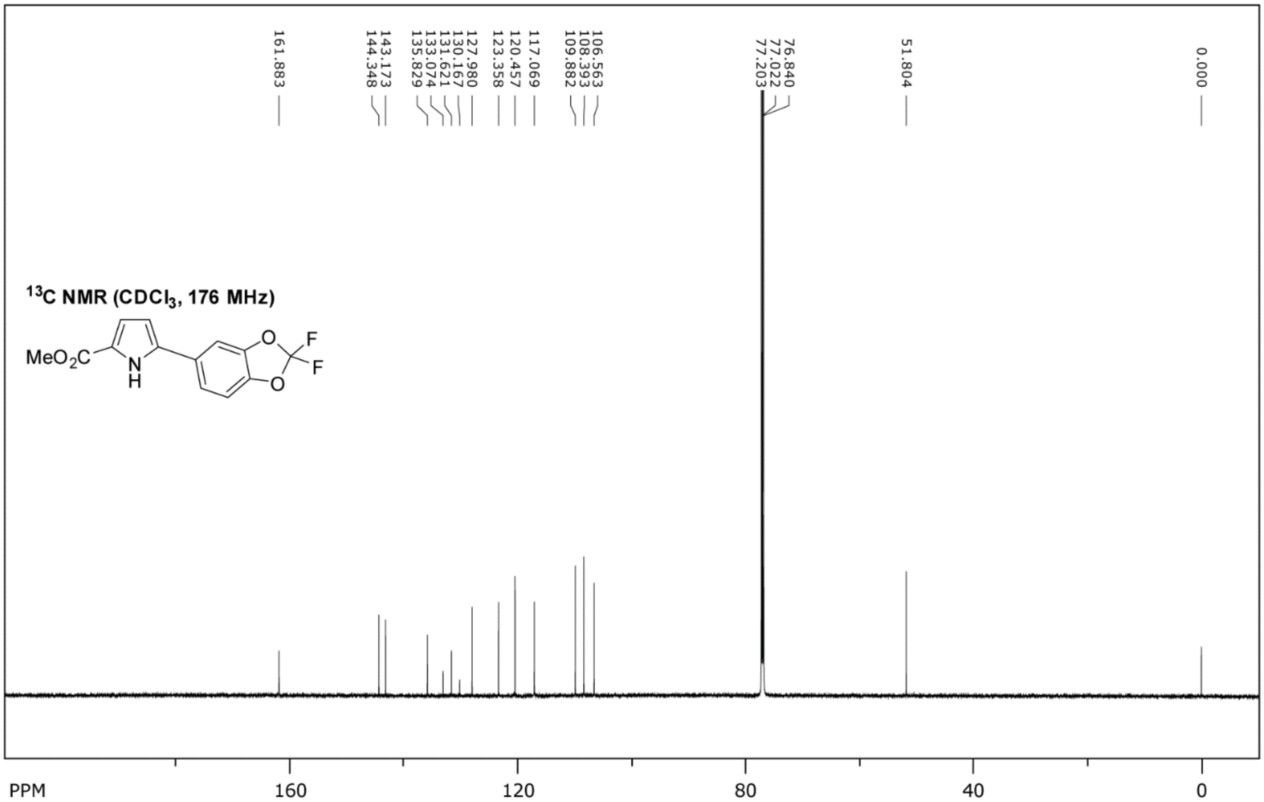

Compound 2u: ^{13}C NMR spectrum of methyl 5-(2,2-difluorobenzo[d][1,3]dioxol-5-yl)-1*H*-pyrrole-2-carboxylate

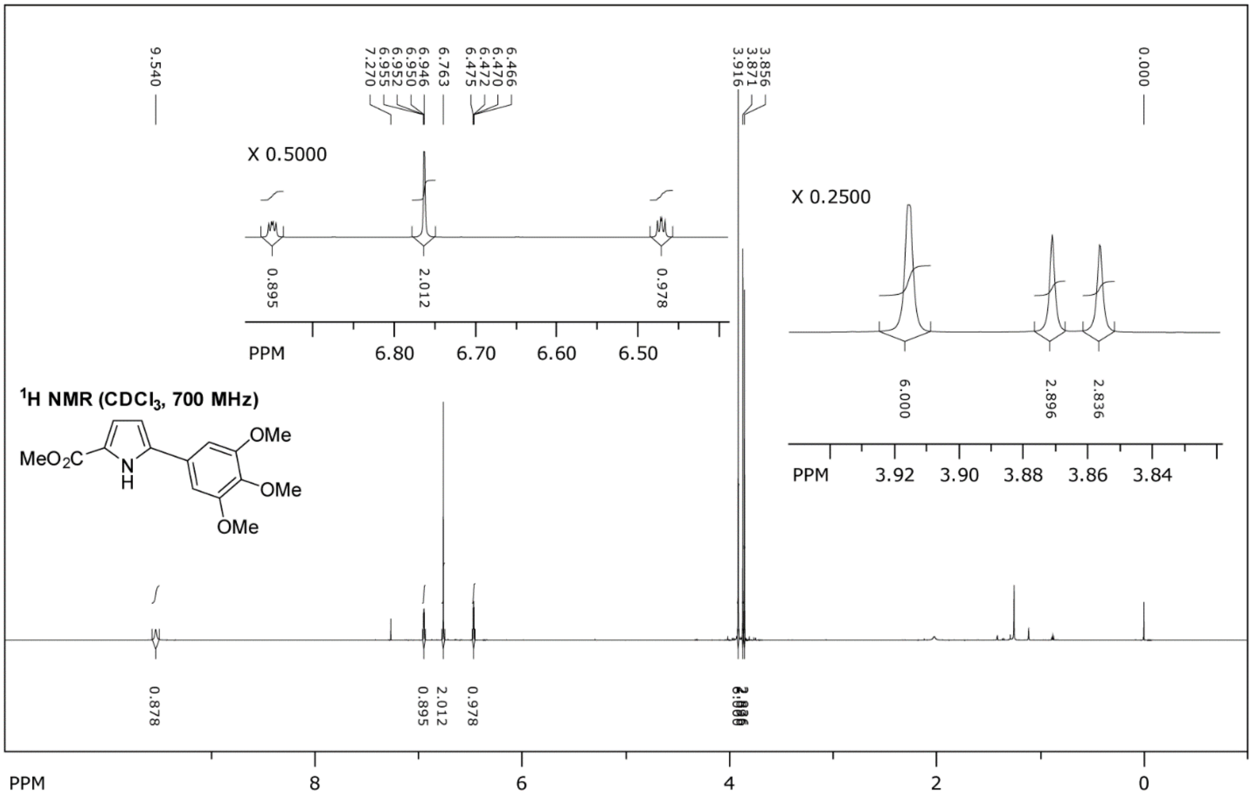

Compound 2v: ^{1}H NMR spectrum of methyl 5-(3,4,5-trimethoxyphenyl)-1*H*-pyrrole-2-carboxylate

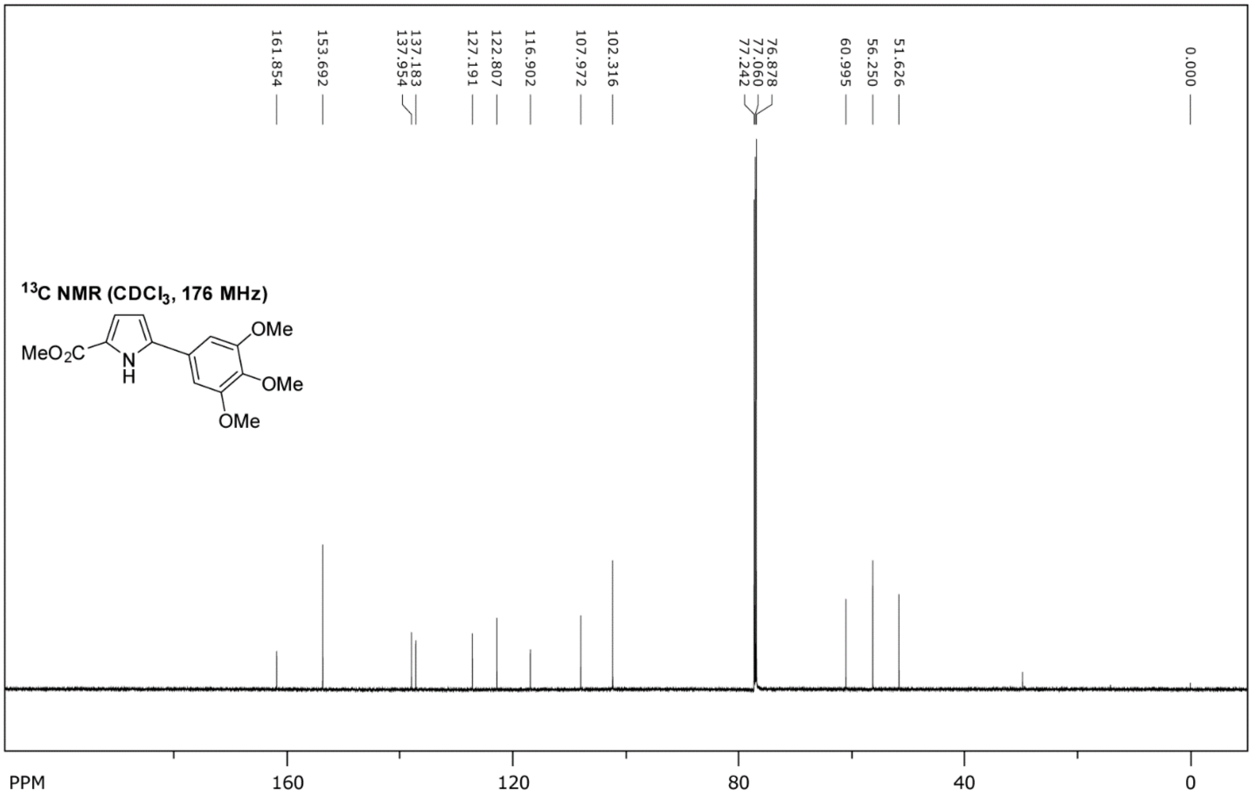

Compound 2v: ^{13}C NMR spectrum of methyl 5-(3,4,5-trimethoxyphenyl)-1*H*-pyrrole-2-carboxylate

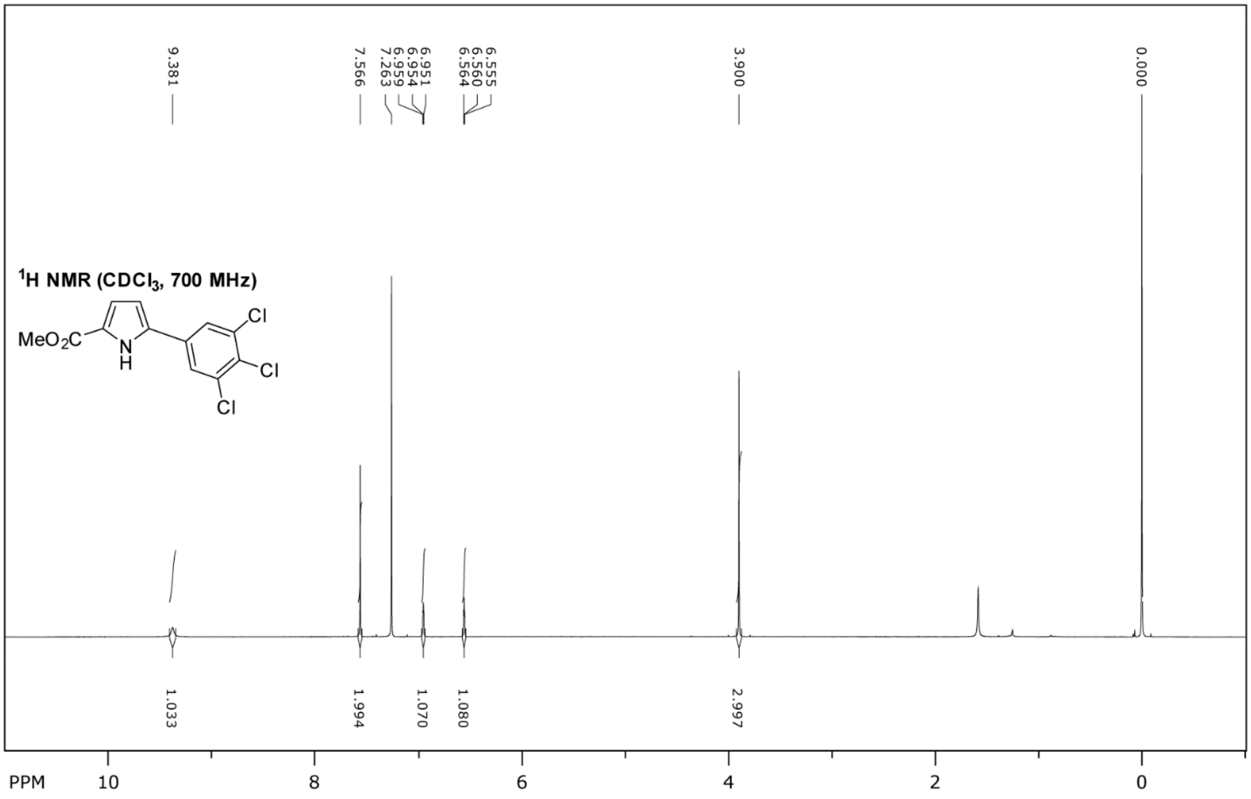

Compound 2w: ^{1}H NMR spectrum of methyl 5-(3,4,5-trichlorophenyl)-1*H*-pyrrole-2-carboxylate

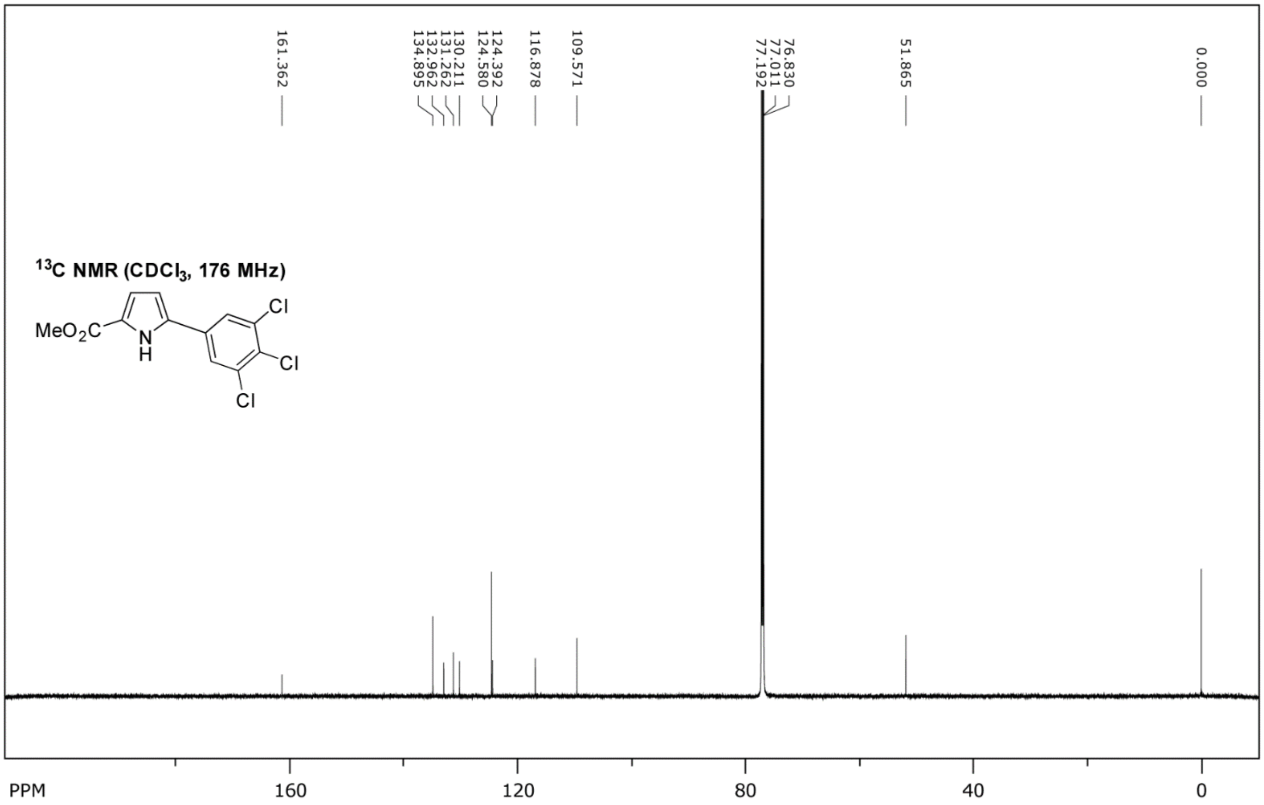

Compound 2w: ^{13}C NMR spectrum of methyl 5-(3,4,5-trichlorophenyl)-1*H*-pyrrole-2-carboxylate

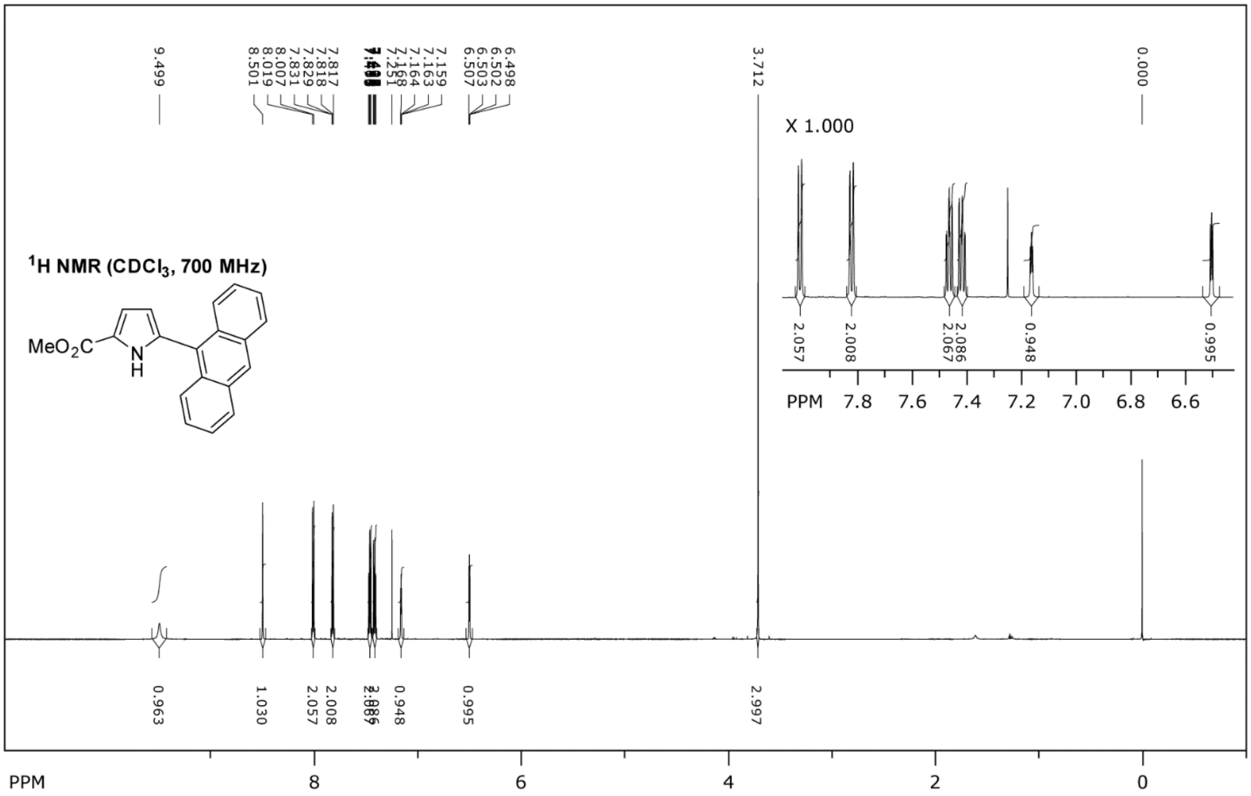

Compound 2x: ^{1}H NMR spectrum of methyl 5-(anthracen-9-yl)-1*H*-pyrrole-2-carboxylate

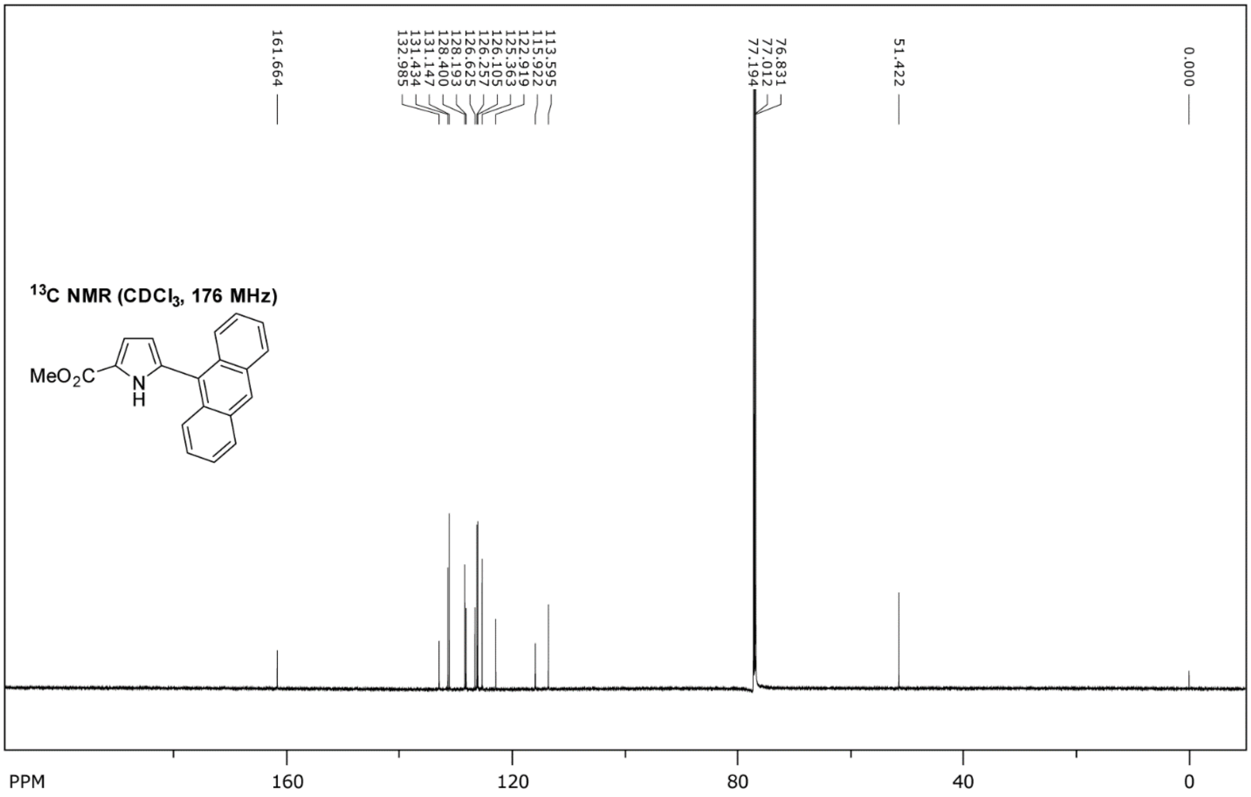

Compound 2x: ^{13}C NMR spectrum of methyl 5-(anthracen-9-yl)-1*H*-pyrrole-2-carboxylate

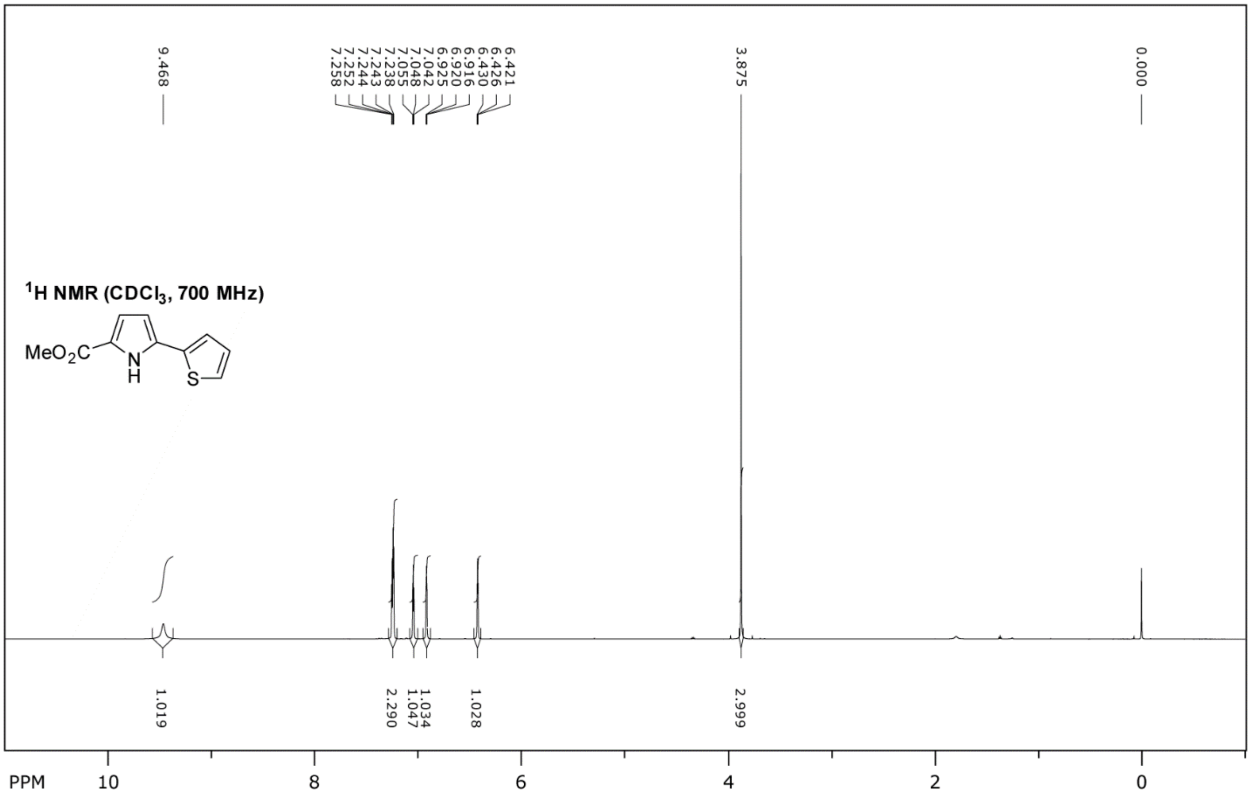

Compound 3a: ^{1}H NMR spectrum of methyl 5-(thiophen-2-yl)-1*H*-pyrrole-2-carboxylate

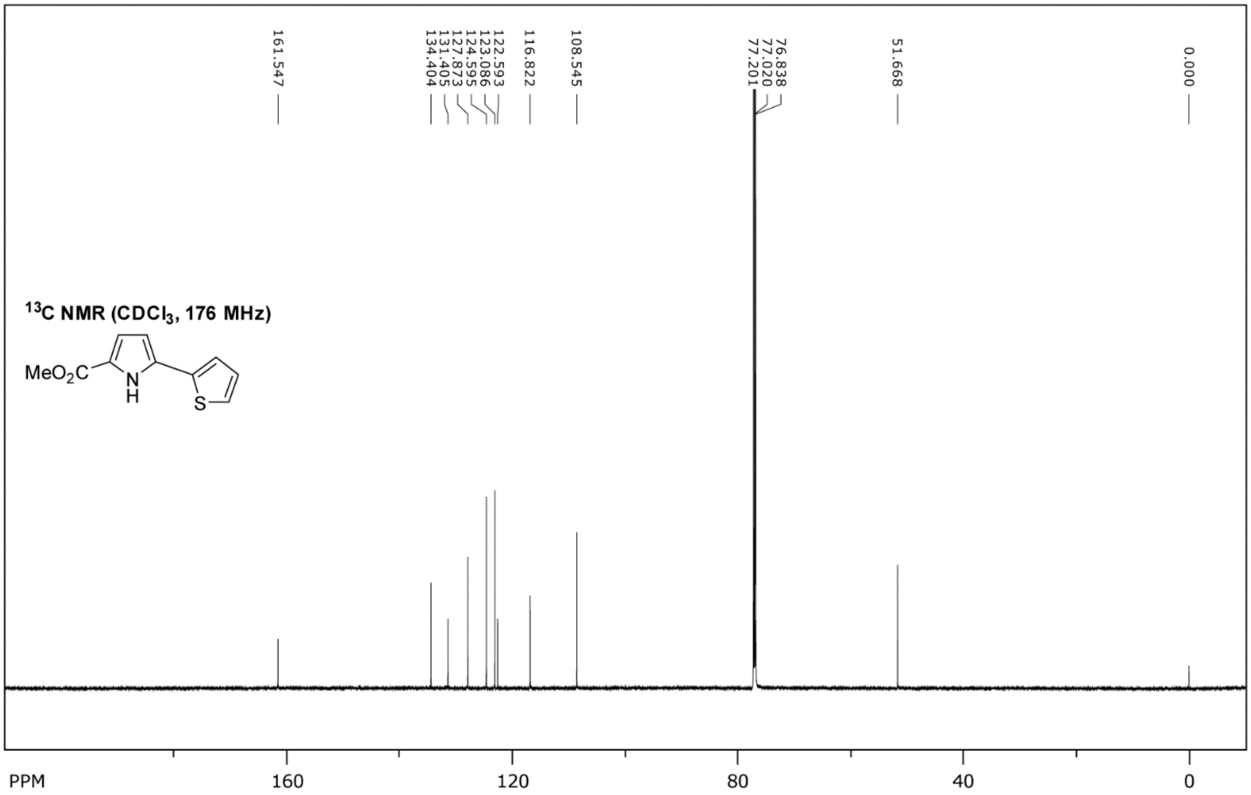

Compound 3a: ^{13}C NMR spectrum of methyl 5-(thiophen-2-yl)-1*H*-pyrrole-2-carboxylate

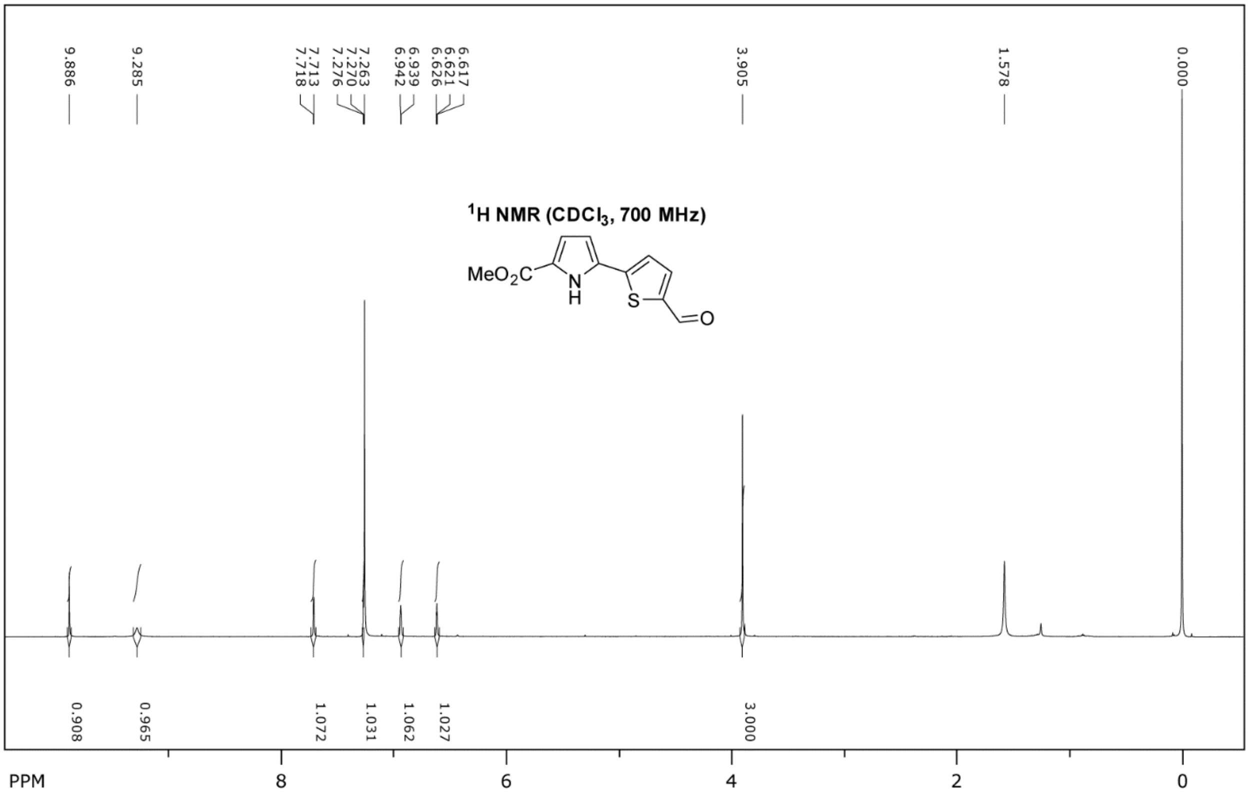

Compound 3b: ^{1}H NMR spectrum of methyl 5-(5-formylthiophen-2-yl)-1*H*-pyrrole-2-carboxylate

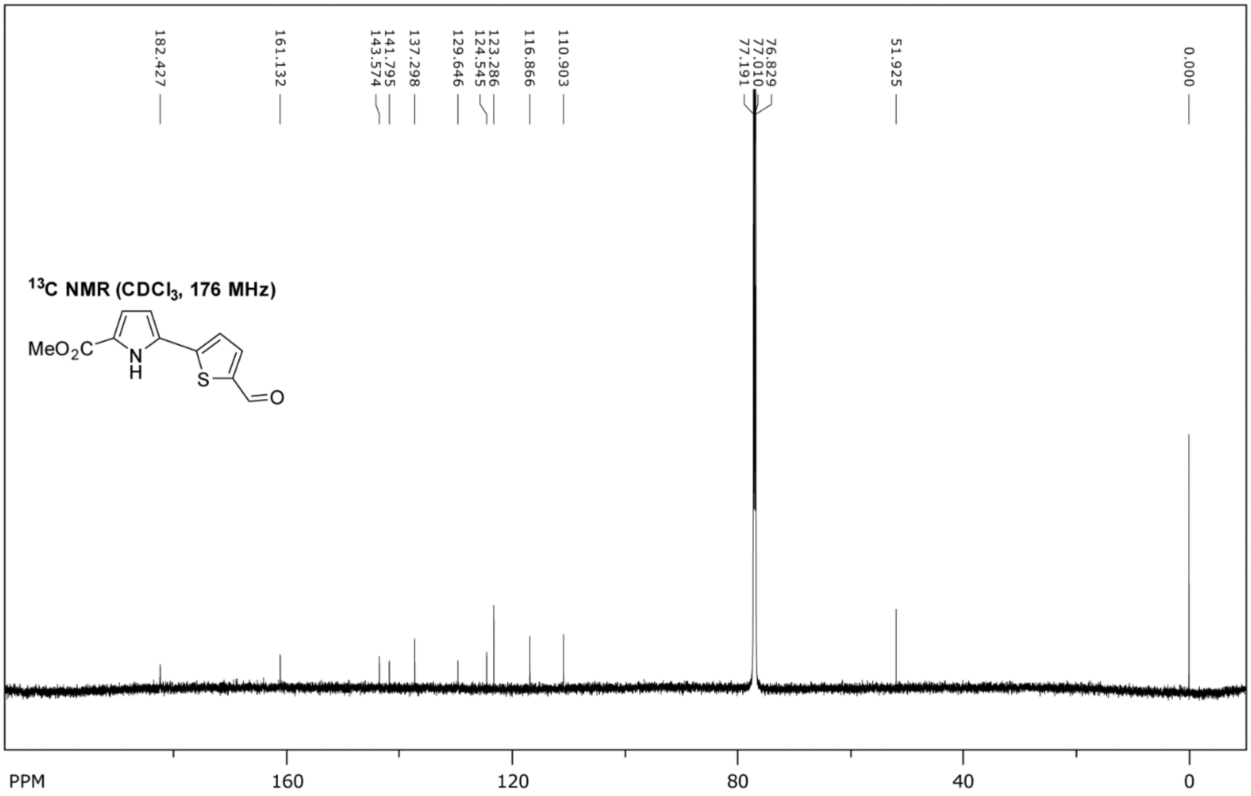

Compound 3b: ^{13}C NMR spectrum of methyl 5-(5-formylthiophen-2-yl)-1*H*-pyrrole-2-carboxylate

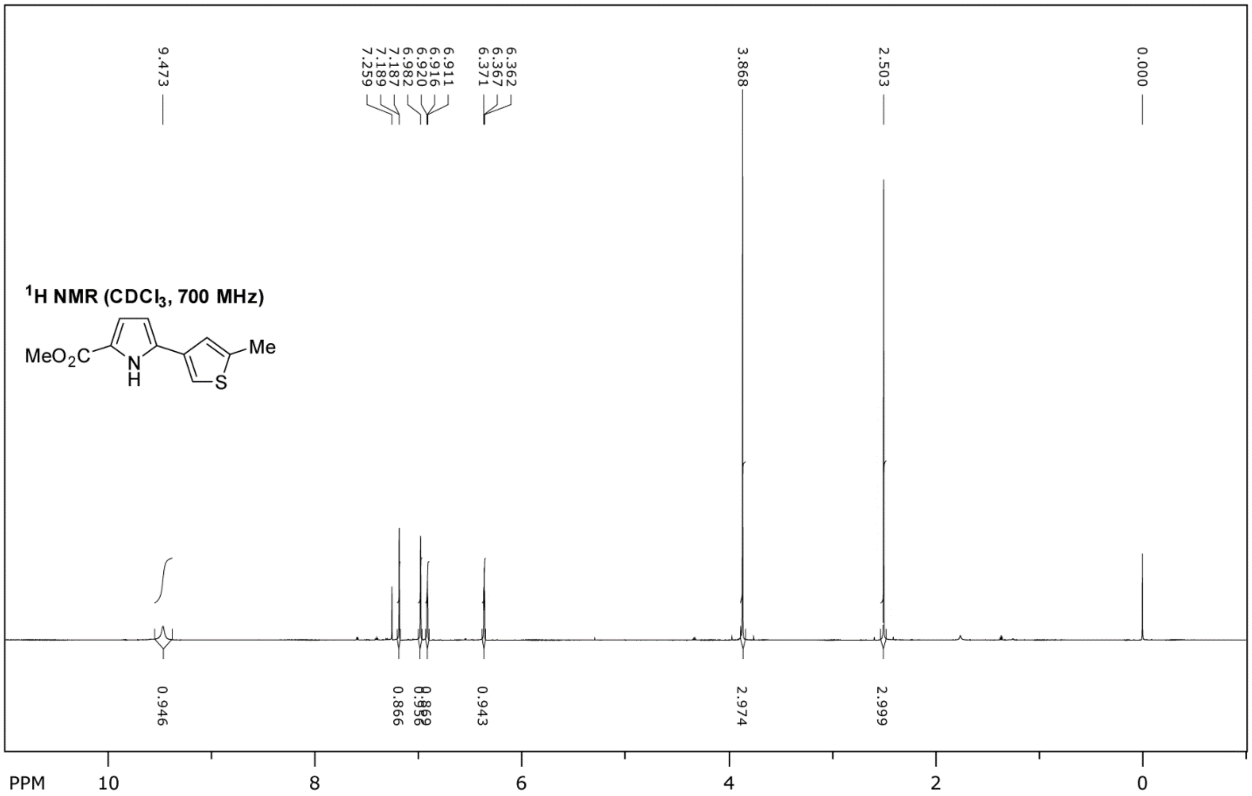

Compound 3c: ¹H NMR spectrum of methyl 5-(5-methylthiophen-3-yl)-1*H*-pyrrole-2-carboxylate

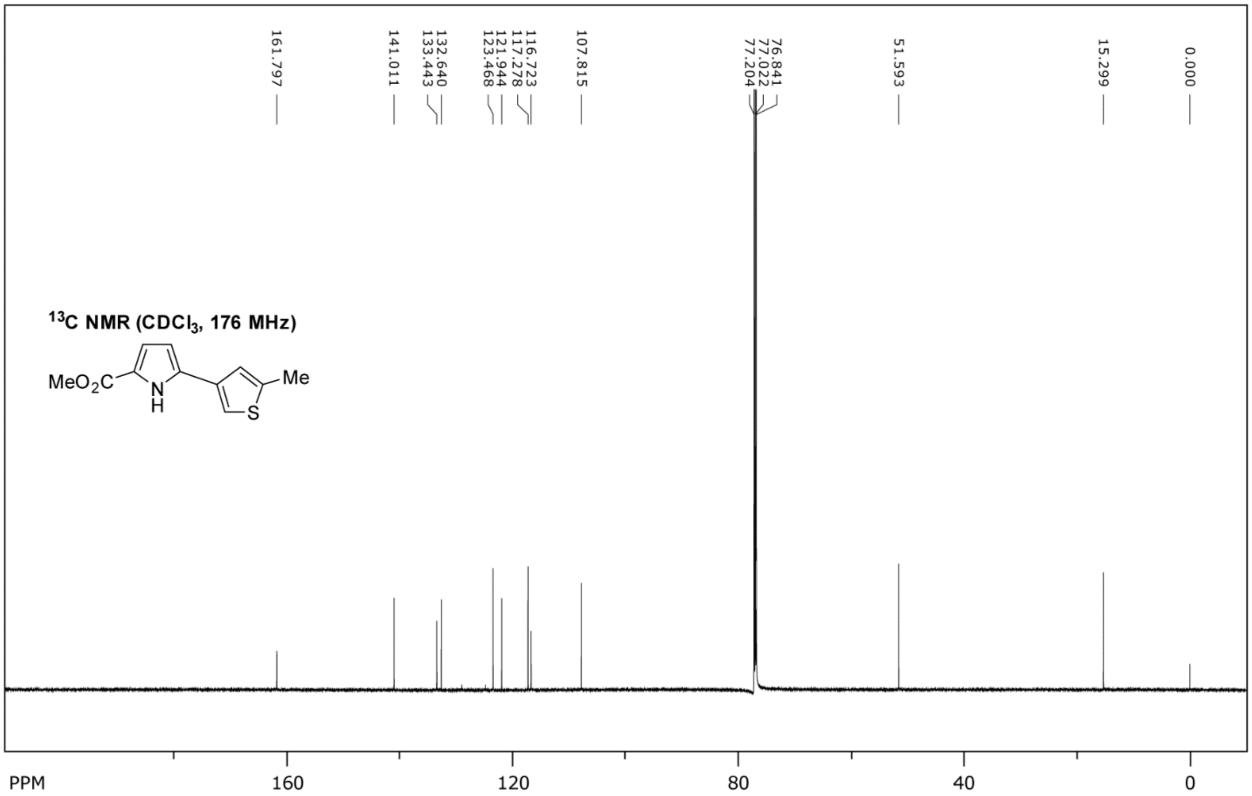

Compound 3c: ¹³C NMR spectrum of methyl 5-(5-methylthiophen-3-yl)-1*H*-pyrrole-2-carboxylate

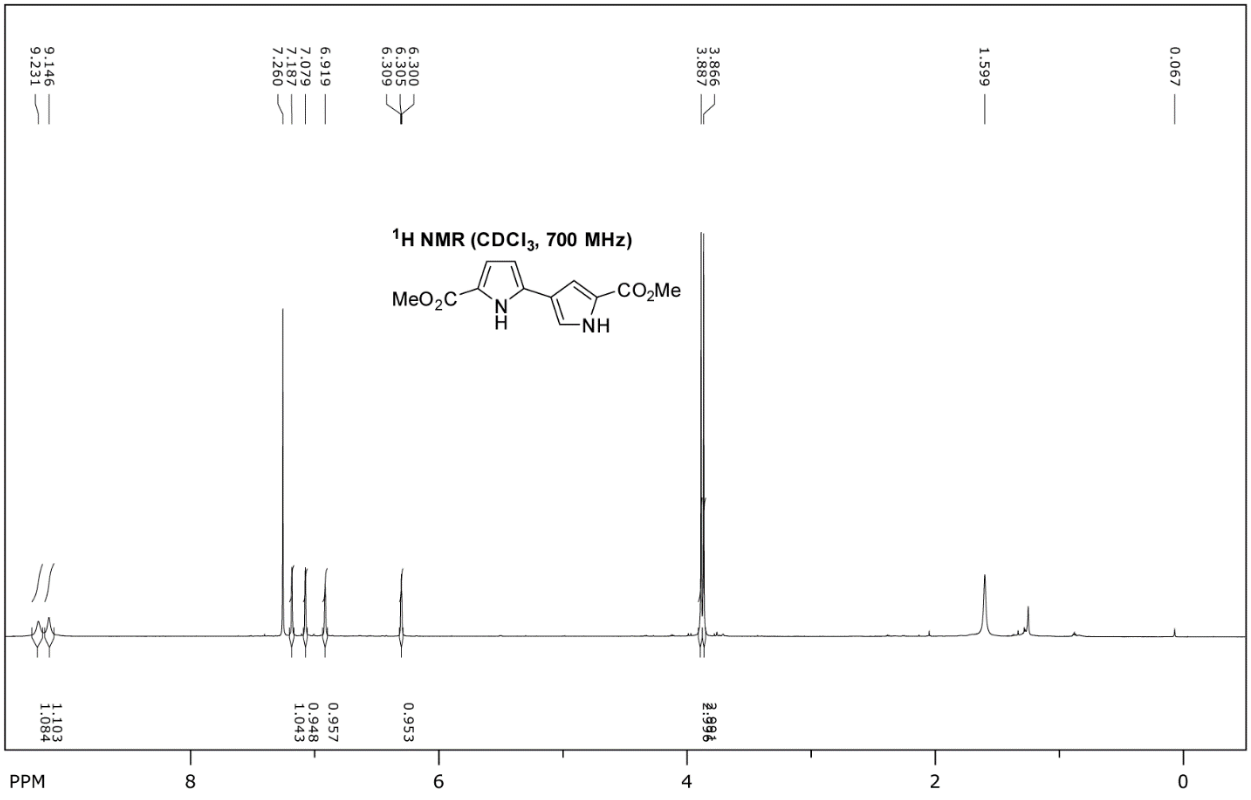

Compound 3d: ^{1}H NMR spectrum of dimethyl 1*H*,1'*H*-[2,3'-bipyrrole]-5,5'-dicarboxylate

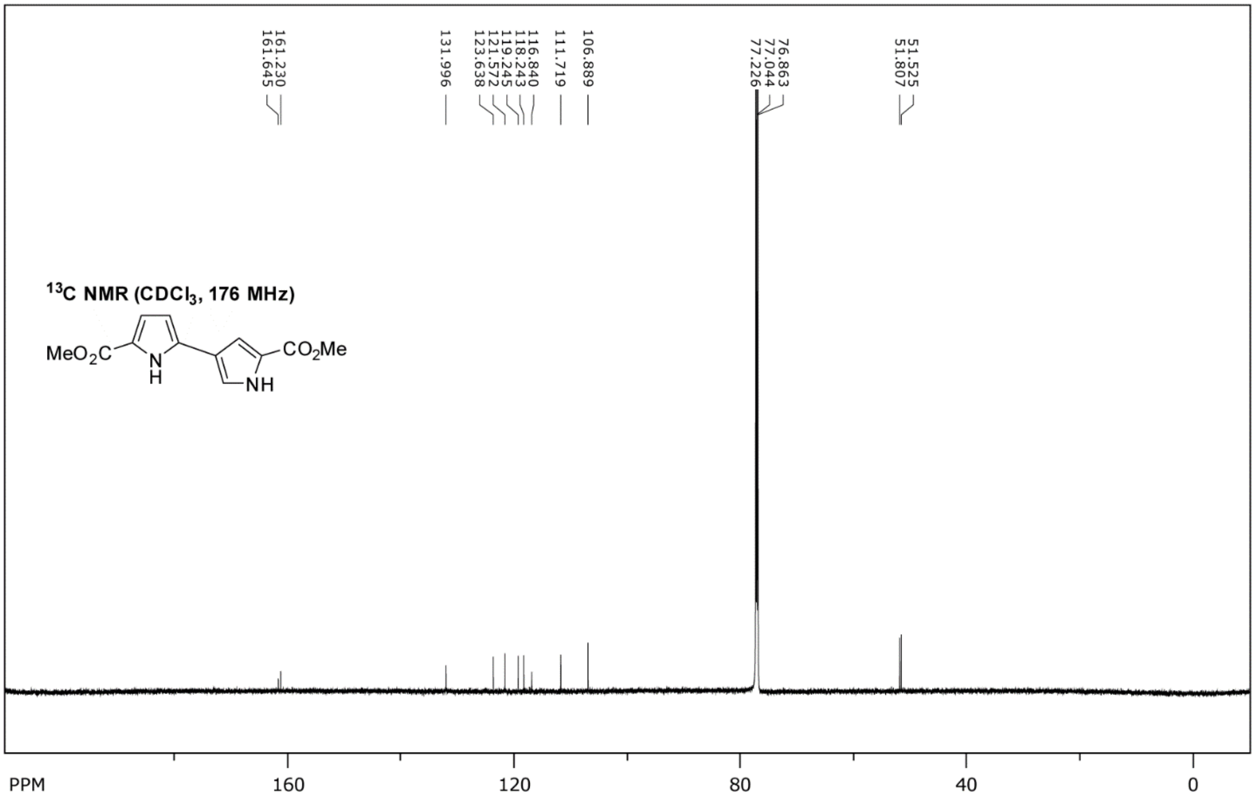

Compound 3d: ^{13}C NMR spectrum of dimethyl 1*H*,1'*H*-[2,3'-bipyrrole]-5,5'-dicarboxylate

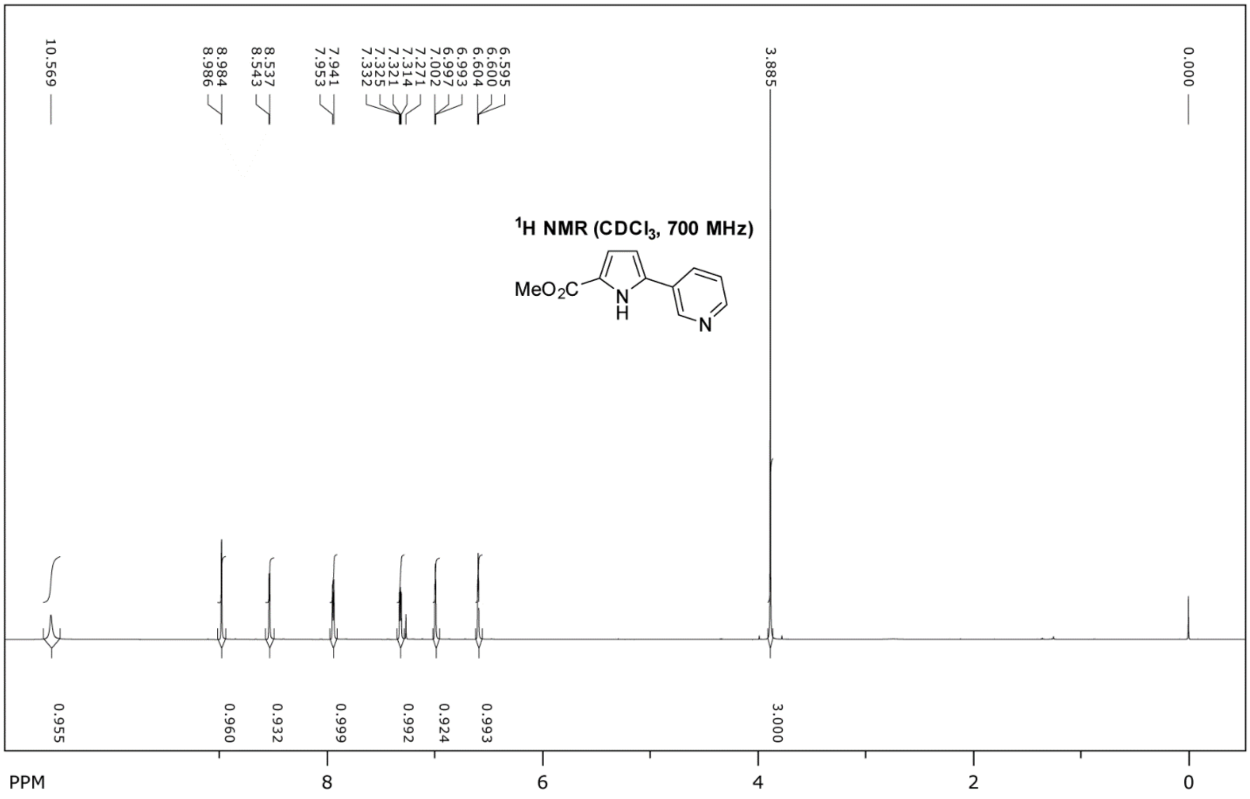

Compound 3e: ^{1}H NMR spectrum of methyl 5-(pyridin-3-yl)-1*H*-pyrrole-2-carboxylate

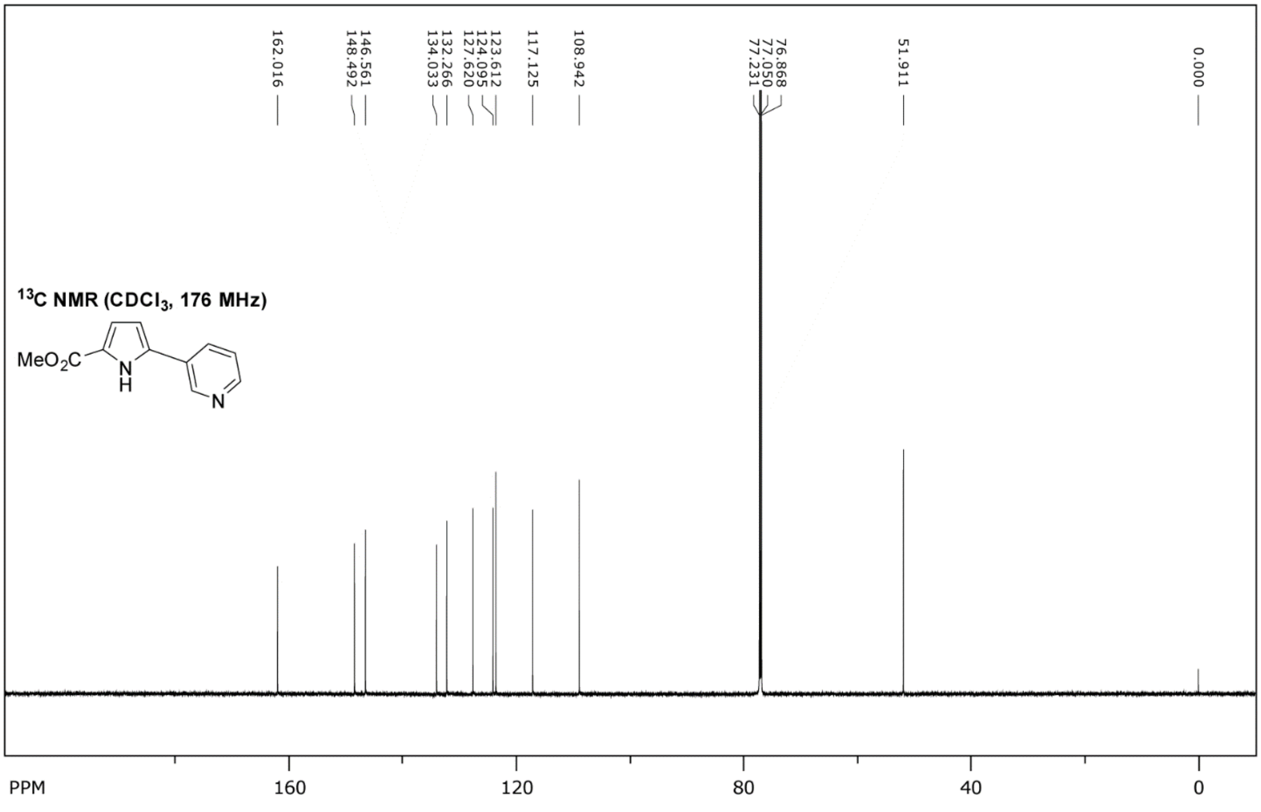

Compound 3e: ^{13}C NMR spectrum of methyl 5-(pyridin-3-yl)-1*H*-pyrrole-2-carboxylate
